# Supplementary material for: A compact GAD67 promoter enables inhibitory neuron-targeted AAV gene therapy for seizure suppression
Source: Mol Ther. 2026 Jun 25;34(9):5510–26. doi: 10.1016/j.ymthe.2026.06.007 (PMC13555566; doi:10.1016/j.ymthe.2026.06.007)
Supplement: Supplementary file 1 [file mmc1.pdf]

YMTHE, Volume *34*

Supplemental Information

A compact GAD67 promoter enables inhibitory neuron-targeted AAV gene therapy for seizure suppression

Yuuki Fukai, Ayumu Konno, Nobutake Hosoi, Karin Miyakawa, Ryosuke Kaneko, and Hirokazu Hirai

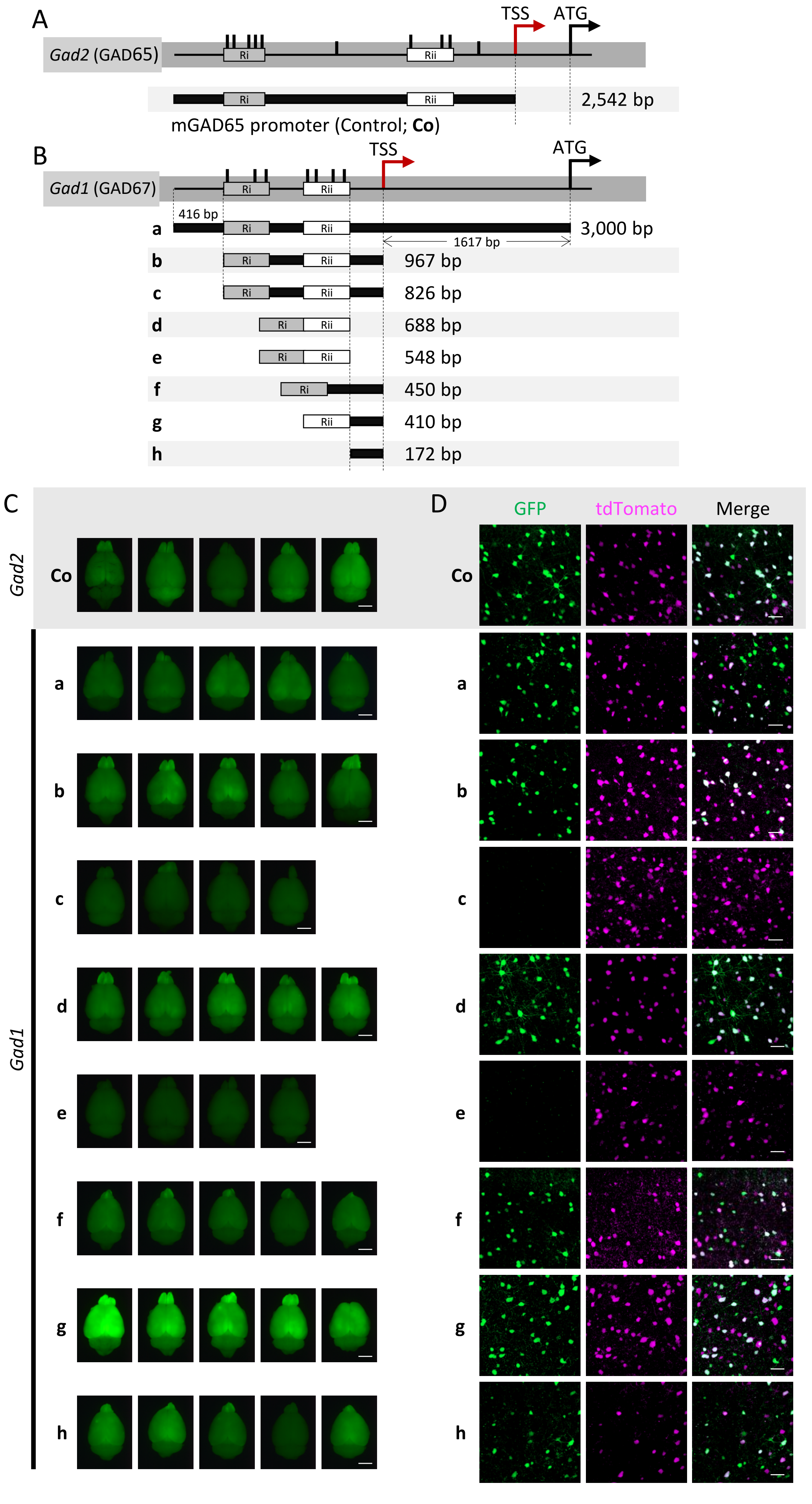

A
TSS
ATG
Gad2 (GAD65)
Ri
Rii
2,542 bp
mGAD65 promoter (Control; Co)
B
TSS
ATG
Gad1 (GAD67)
Ri
Rii
416 bp
a
3,000 bp
1617 bp
b
967 bp
c
826 bp
d
688 bp
e
548 bp
f
450 bp
g
410 bp
h
172 bp
C
Gad2
Co
Gad1
a
b
c
d
e
f
g
h
D
GFP
tdTomato
Merge
Co
a
b
c
d
e
f
g
h

Figure S1. Strategy for identifying a minimal promoter region specific to GABAergic neurons.

(A) Schematic representation of the *Gad2* locus. Vertical bars indicate binding motifs for the inhibitory neuron–specific transcription factor Dlx. Gray (Region i, Ri) and white (Region ii, Rii) boxes represent regions enriched in Dlx binding motifs. The 2,542 bp region upstream of the transcription start site (TSS) corresponds to the previously reported inhibitory neuron–specific mGAD65(delE1) promoter, which was used as a control (Co). (B) Schematic representation of the *Gad1* locus and promoter candidates. Promoter candidates were initially cloned from ~3 kb upstream of the ATG start codon and subsequently truncated based on the TSS, retaining either or both Ri and Rii regions. (C) Whole-brain GFP fluorescence images from mice intravenously injected with PHP.eB vectors (100 µL, 5.0×10^{11} vg/mouse) expressing GFP under the control of the control mGAD65(delE1) promoter (Co) or mGAD67 promoter candidates (a–h). Scale bars, 5 mm. (D) Representative native fluorescence images of GFP and tdTomato in the motor cortex, along with merged images. Scale bars, 50 µm.

Sequence of cmGAD67 promoter

Rii: -410 to -142
Putative core promoter: -143 to -1

ACAAAATTCTTCGTAGGAATTATATTTTCCCTTGCCCTCACCCAACATC
GCCTATCTCAAAAATAATTTAAAAACAAAAAACAAACAAACAAACAA
AAAACAGAGCGTGCTGAGTGCATTCTGGATTACTCATAGGACTTTGT
CACACACACCCTCCTTTCTGGTCGCAAACCCGTGAGCTGGATTTATA
ATCGCCCTACAAAGCTCCAGAGGCAGTCAGACACCTGCAAAGGAGC
CCCAGCGCTCCGCGGACGAGCTGCCCCCGCGAGCAACGGCCTCGT
GATTCCCCCCGCCGAGCGGGTCCCCGCCTCCCCACTCCGCCCCCGCC
TCCCCCAAGCCCAGCGGCCGCCTCTCCGGATCTCTCCCTTCTTCAGG
CTCTCCCGTGCCGGACCAGGGATCGTGCAAGCAAG

Underlined sequences indicate TAAT/ATTA motifs, which are putative DLX-binding sites.
*+1 = Transcription start site (TSS)

Figure S2. Nucleotide sequence and structural features of the cmGAD67 promoter.
The 410-bp cmGAD67 promoter sequence is shown with key regulatory elements annotated. The transcription start site (TSS) is defined as +1, and nucleotide positions are shown relative to the TSS. The core promoter region (light blue background) and the Rii enhancer region (yellow background) are highlighted. Putative Dlx binding motifs (TAAT/ATTA) within the Rii region are indicated by underlining. This sequence corresponds to the cmGAD67 promoter construct shown in Fig. 1C (g).

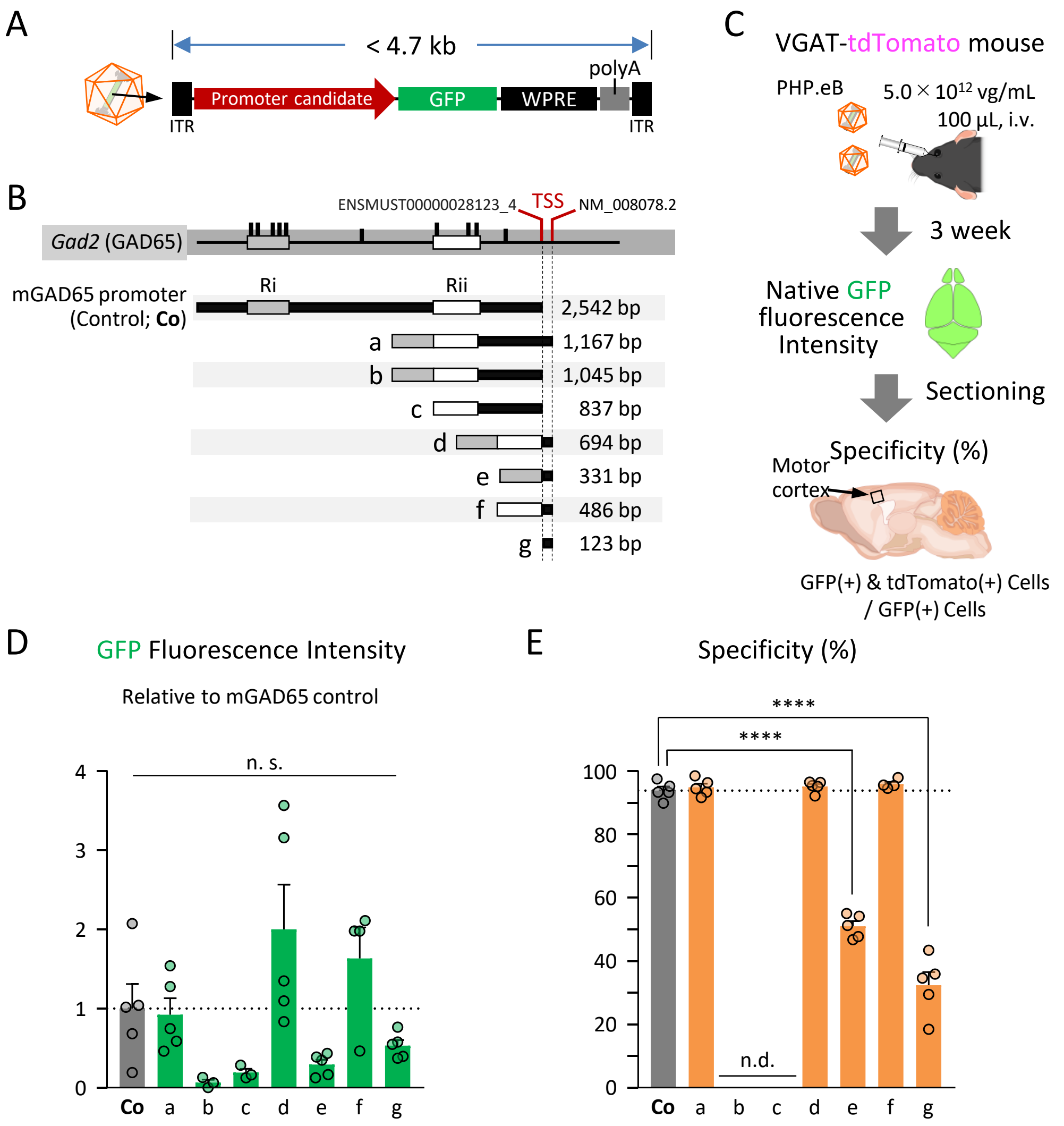

Figure S3. Characterization of the compact GAD65 promoter (cmGAD65).
(A) Schematic diagram of the AAV genome used in this study. When GFP, WPRE, and SV40 polyA are included between the ITRs, the promoter sequence must be shorter than 3 kb to remain within the AAV packaging limit (~4.7 kb). (B) Schematic representation of the *Gad2* locus and promoter candidates. Vertical bars indicate binding motifs for the inhibitory neuron–specific transcription factor Dlx. Gray (Region i, Ri) and white (Region ii, Rii) boxes represent regions enriched in Dlx binding motifs. The 2,542 bp region upstream of the transcription start site (TSS) (from genome database ID: ENSMUST00000028123_4) corresponds to the previously reported inhibitory neuron–specific mGAD65(delE1) promoter, which was used as a control (Co). In the present study, we used the NM_008078.2 TSS as a reference, and promoter candidates were produced based on the TSS, retaining either or both Ri and Rii regions (a – g). (C) Experimental design. AAV-PHP.eB vectors expressing GFP under the control of *Gad2*-derived promoter candidates (100 µL, 5.0×10^{11} vg/mouse) were intravenously administered via the retro-orbital sinus into VGAT-tdTomato mice, in which inhibitory neurons express the red fluorescent protein tdTomato. Three weeks after injection, mice were perfused, and GFP fluorescence on the dorsal surface of the whole brain was imaged to assess promoter activity. Sagittal sections were then prepared, and inhibitory neuron specificity of GFP expression was quantified in the M1/M2 motor cortex. (D) Quantification of dorsal whole-brain GFP fluorescence intensity. Fluorescence intensity in the mGAD65(delE1) control group was normalized to 1. (E) Quantification of the proportion of GFP-positive cells that were also tdTomato-positive (indicative of inhibitory neuron specificity). Each dot represents an individual mouse. ****$p < 0.0001$, n.s., not significant by one-way ANOVA with Dunnett's post hoc test. n.d., not determined.

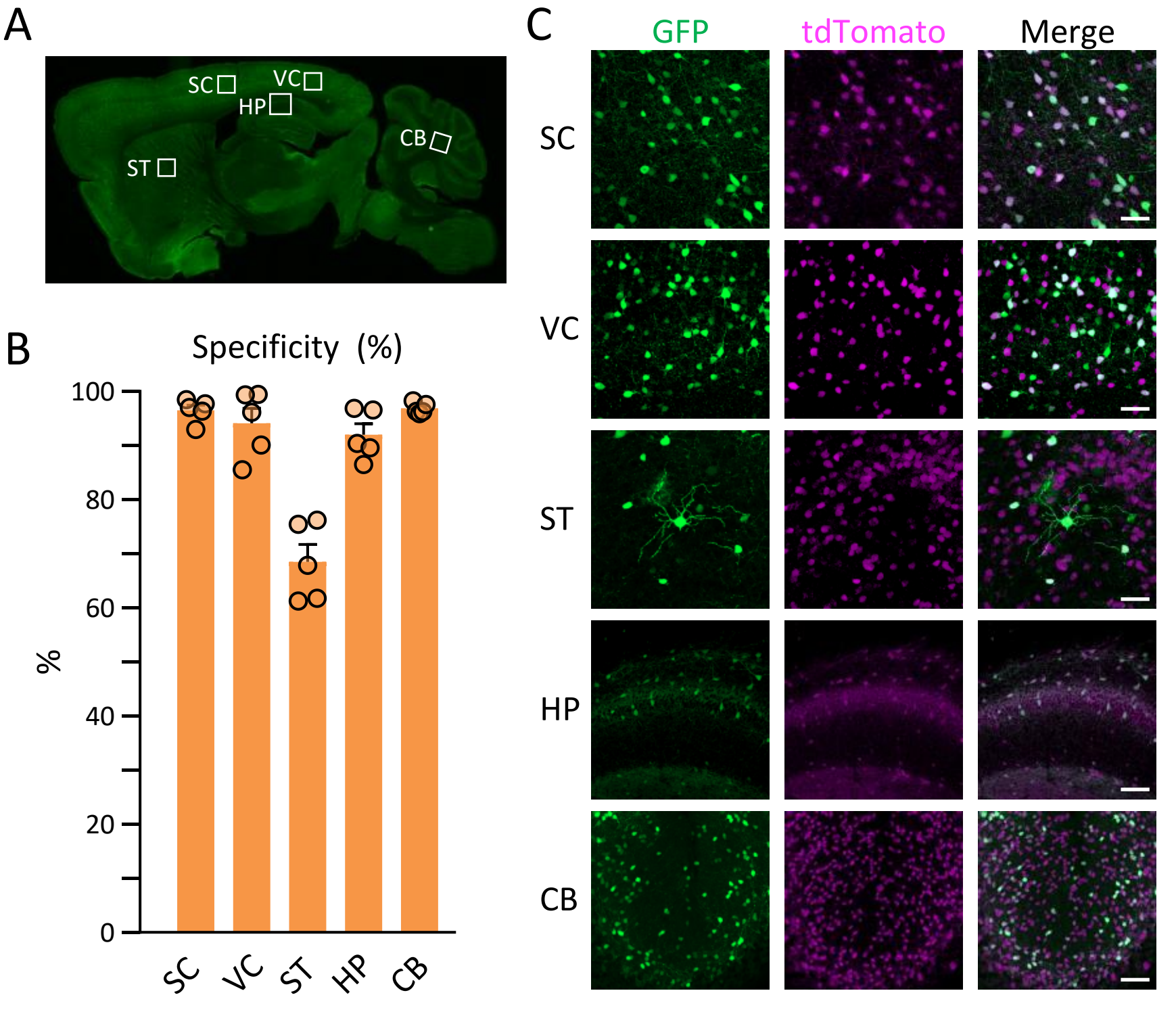

Figure S4. Inhibitory neuron specificity of the cmGAD67 promoter across multiple brain regions in mice

Inhibitory neuron specificity of the cmGAD67 promoter was evaluated across multiple brain regions beyond the motor cortex. VGAT-tdTomato mice were intravenously injected with AAV-PHP.eB-cmGAD67-GFP (100 µL, 5.0 × 10^{11} vg/mouse). Three weeks after injection, brains were perfusion-fixed, and sagittal sections were prepared for analysis. (A) Representative sagittal brain section showing GFP fluorescence. Major regions are indicated: SC, sensory cortex; VC, visual cortex; ST, striatum; HP, hippocampus; CB, cerebellum. (B) Quantification of inhibitory neuron specificity in each brain region, defined as the percentage of tdTomato-positive cells among GFP-expressing cells within a defined area (542.5 µm × 722.4 µm). Each dot represents one mouse (n = 5), and bars indicate mean ± SEM. High co-localization indicates robust inhibitory neuron specificity across all examined regions. (C) Higher-magnification images of the boxed regions in (A), showing GFP (green), tdTomato (magenta), and merged signals. Scale bars, 50 µm.

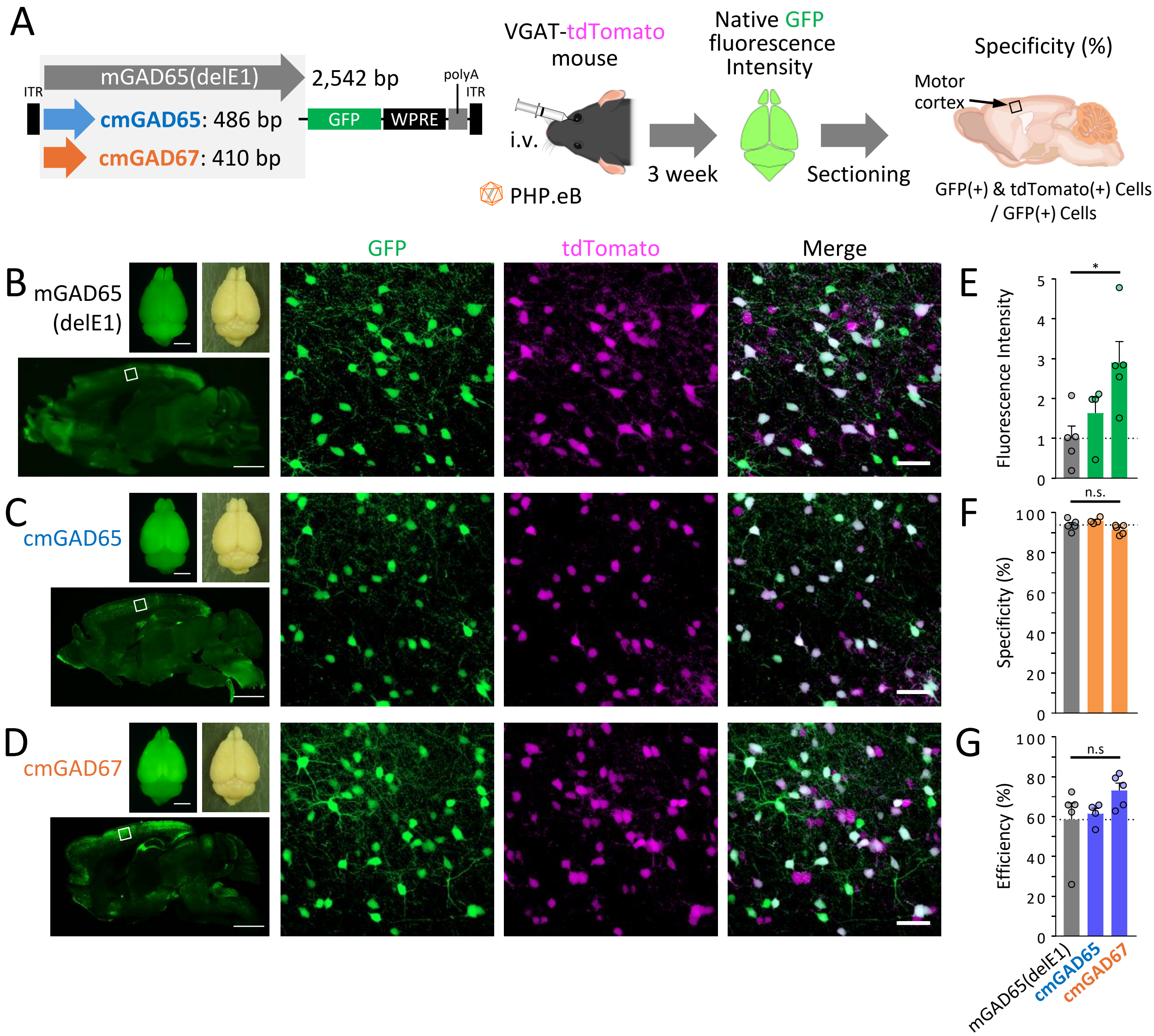

Figure S5. Comparative analysis of expression profile of mGAD(delE1) and cmGAD promoters after intravenous administration of AAV-PHP.be

(A) Schematic representation of the experimental procedure. AAV-PHP.eB expressing GFP under the control of the conventional mGAD65(delE1) promoter, cmGAD65 promoter, or cmGAD67 promoter was intravenously administered to VGAT-tdTomato mice. Three weeks post-injection, whole-brain GFP fluorescence intensity was measured. Sagittal brain sections were then prepared to assess the specificity of GFP expression in inhibitory neurons within the primary motor cortex. (B-D) Representative images of GFP fluorescence and bright-field views of the whole brain (top left paired images) and sagittal section GFP fluorescence images (bottom left) for the mGAD65(delE1) (B), cmGAD65 (C), and cmGAD67 (D) promoters. The three rightmost columns show magnified fluorescence images of the primary motor cortex (corresponding to the boxed region in the bottom left images). Scale bars, 5 mm (whole brains), 2 mm (sagittal sections) and 50 µm (right panels). (E) Quantification of whole-brain GFP fluorescence intensity except for the cerebellar area. Relative intensities are shown with the mGAD65(delE1) promoter set as 1. (F) Graph showing the specificity of GFP expression in inhibitory neurons. (G) Quantification of the proportion (efficiency) of inhibitory neurons in the primary motor cortex expressing GFP. Dots in the graphs represent individual mouse data points. *$p < 0.05$, n.s., not significant by one-way ANOVA with Dunnett's post hoc test.

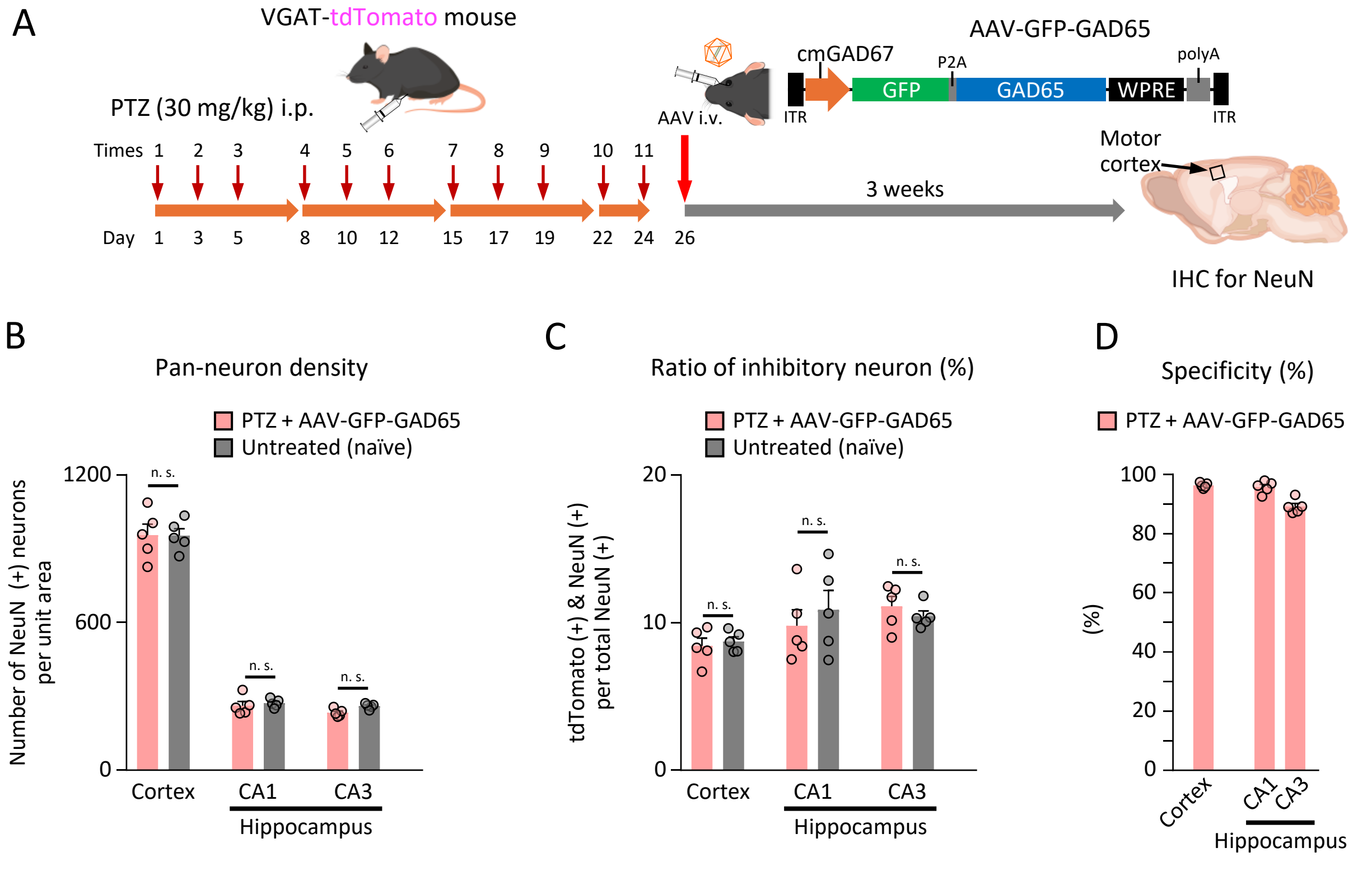

Figure S6. Inhibitory neuron proportion and AAV-cmGAD67-GAD65 expression specificity are preserved after PTZ treatment.

(A) Experimental design. VGAT-tdTomato mice, in which inhibitory neurons are labeled with the red fluorescent protein tdTomato, received 11 intraperitoneal injections of PTZ (30 mg/kg). Two days after the final injection, AAV-PHP.eB-cmGAD67-GFP-P2A-GAD65 was administered intravenously. Three weeks later, brains were perfusion-fixed, and sagittal sections were prepared for immunohistochemical analysis using an anti-NeuN antibody. Analyses were performed in the motor cortex (M1/M2) and hippocampal CA1 and CA3 regions. Untreated VGAT-tdTomato mice (no PTZ or AAV) were used as controls. (B) No significant difference in the number of NeuN-positive neurons was observed between PTZ-treated (pink bars) and untreated (gray bars) groups (n = 5 mice per group). Quantification was performed within images of a defined area (542.5 μm × 722.4 μm). (C) The proportion of tdTomato-positive inhibitory neurons among NeuN-positive neurons did not differ significantly between PTZ-treated and untreated groups (n = 5 mice per group), based on quantification within the same defined area (542.5 μm × 722.4 μm). (D) In mice that received 11 PTZ injections, inhibitory neuron specificity of AAV-cmGAD67-GAD65 expression remained well preserved (n = 5 mice). n.s., not significant by unpaired two-tailed Student's t-test.

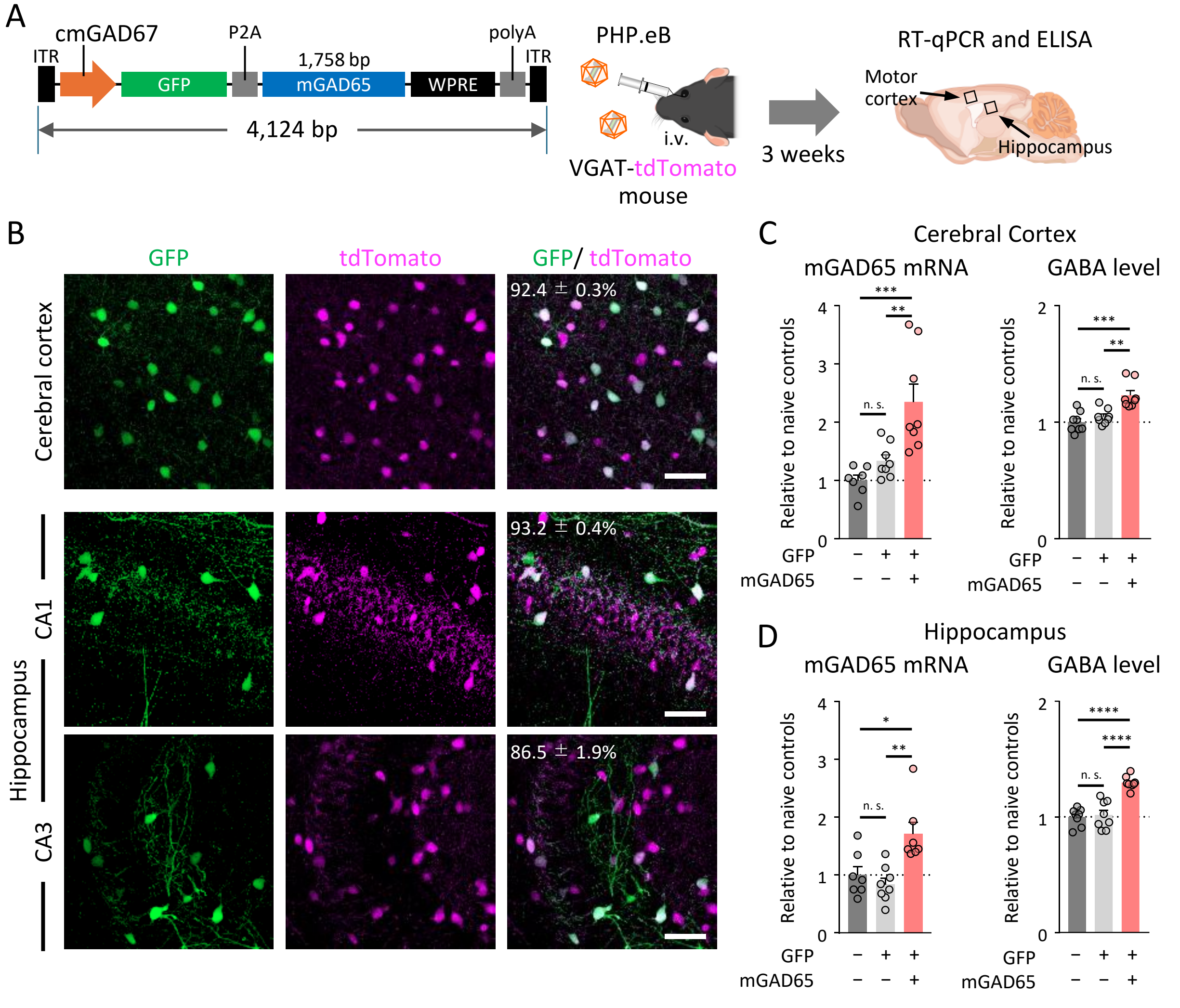

Figure S7. AAV-cmGAD67-GAD65 elevates cortical and hippocampal GABA levels.
(A) Schematic representation of the experimental procedure. AAV-PHP.eB expressing GFP and *mGAD65* under the control of the cmGAD67 promoter was intravenously administered to VGAT-tdTomato mice. Three weeks post-injection, tissue samples from the primary motor cortex and hippocampus were collected for quantification of *mGAD65* mRNA using RT-qPCR and measurement of GABA levels via ELISA. (B) Fluorescence images of native GFP and native tdTomato in the primary motor cortex and hippocampal CA1 and CA3 regions. The numbers in the upper left corner of the rightmost panel indicate the percentage of tdTomato-positive (inhibitory) neurons among GFP (*mGAD65*)-expressing cells (Average ± SEM., $n = 5$ mice). Scale bars, 50 μm. (C,D) Quantification of *mGAD65* mRNA (left) and GABA levels (right) in the primary motor cortex (C) and hippocampus (D). The values were normalized to those obtained from untreated mice (leftmost bar). Relative expression levels are shown for mice injected with AAV-PHP.eB expressing GFP alone and those expressing both GFP and *mGAD65*. Dots in the graphs represent individual mouse data points. $*p < 0.05$, $**p < 0.01$, $***p < 0.001$, $****p < 0.0001$, n.s., not significant; one-way ANOVA with Tukey's post hoc test.

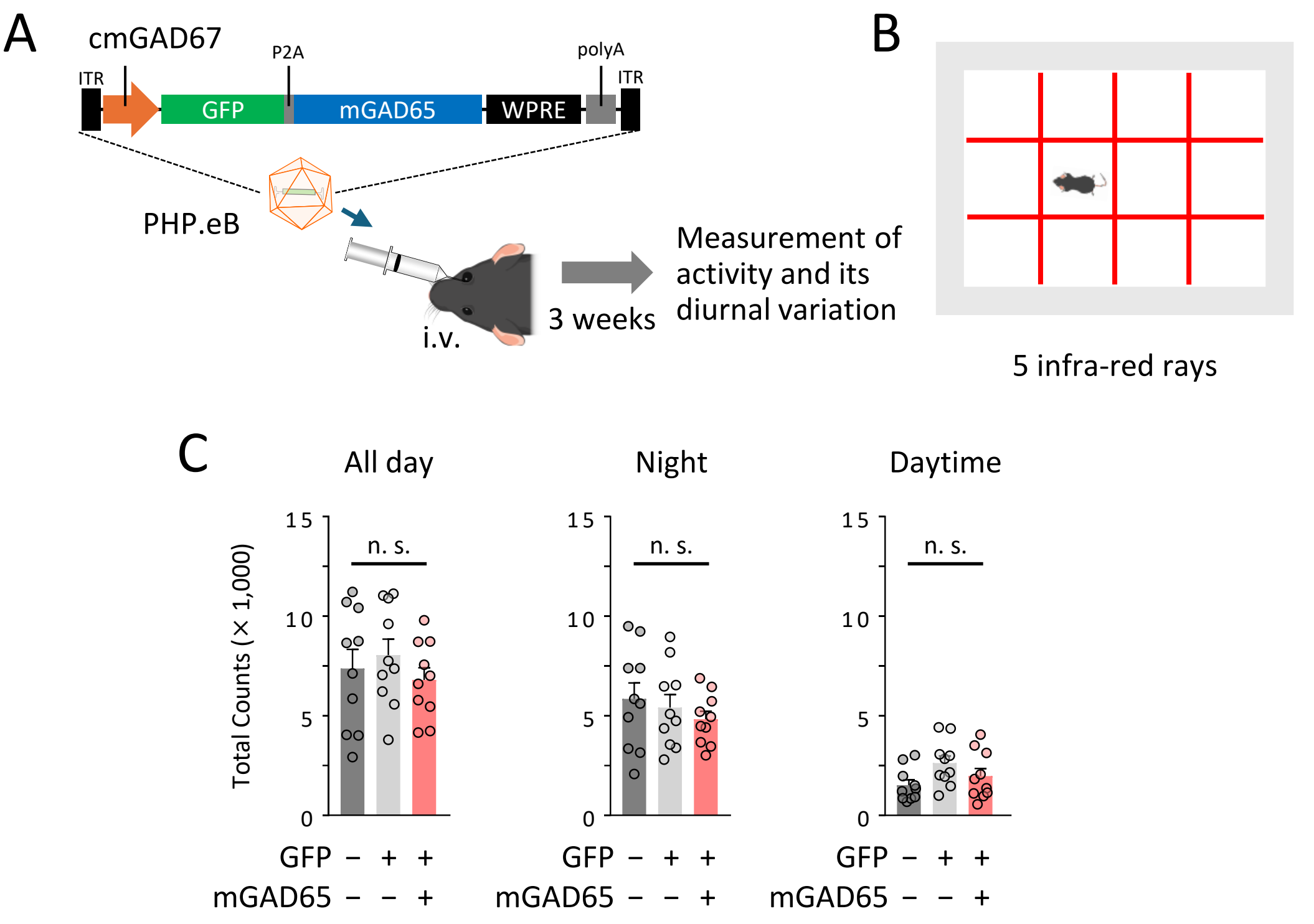

Figure S8. Safety assessment: AAV-cmGAD67-GAD65 does not alter circadian activity.

(A) Schematic representation of the experimental procedure. AAV-PHP.eB expressing GFP and GAD65 under the control of the cmGAD67 promoter was intravenously administered. Three weeks post-injection, locomotor activity and its circadian variations were measured. (B) Illustration of the activity monitoring apparatus. Mice were placed in a cage segmented by five horizontal and five vertical infrared beams, and locomotor activity was quantified by counting the number of infrared beam crossings. (C) Quantification of the number of infrared beam crossings over the entire 24-hour period, during the nocturnal phase, and during the diurnal phase. Dots in the graph represent individual mouse data points. n.s., not significant; one-way ANOVA with Tukey's post hoc test.

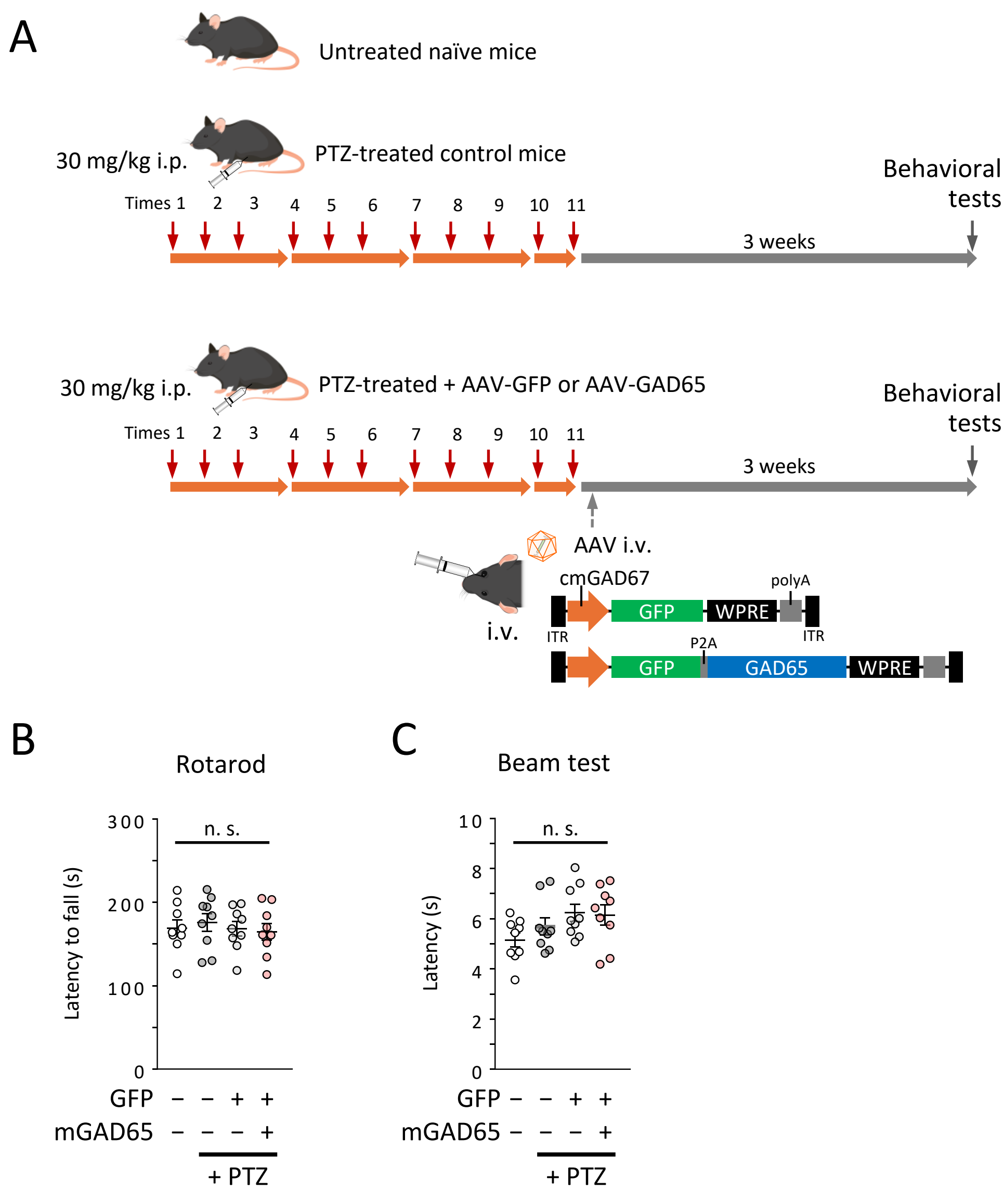

Figure S9. Safety assessment: AAV-cmGAD67-GAD65 does not impair motor coordination.
(A) Schematic representation of the experimental procedure. Naïve mice were used as controls. The rest of three groups received pentylenetetrazole (PTZ, 30 mg/kg) intraperitoneally three times per week (every other day) for a total of 11 sessions. One group were treated only with PTZ. Two other groups received intravenous injection of AAV-PHP.eB expressing GFP-P2A-GAD65 or GFP alone under the control of the cmGAD67 promoter. Behavioral experiments were conducted three weeks later. (B) Results of the rotarod test. The latency to fall from the rotating rod was measured. (C) Results of the beam test. Mice were placed at one end of a narrow metal beam, and the time taken to traverse the beam and enter a shelter at the opposite end was recorded. Dots in the graphs represent individual mouse data points. n.s., not significant; ANOVA with Tukey's post hoc test.

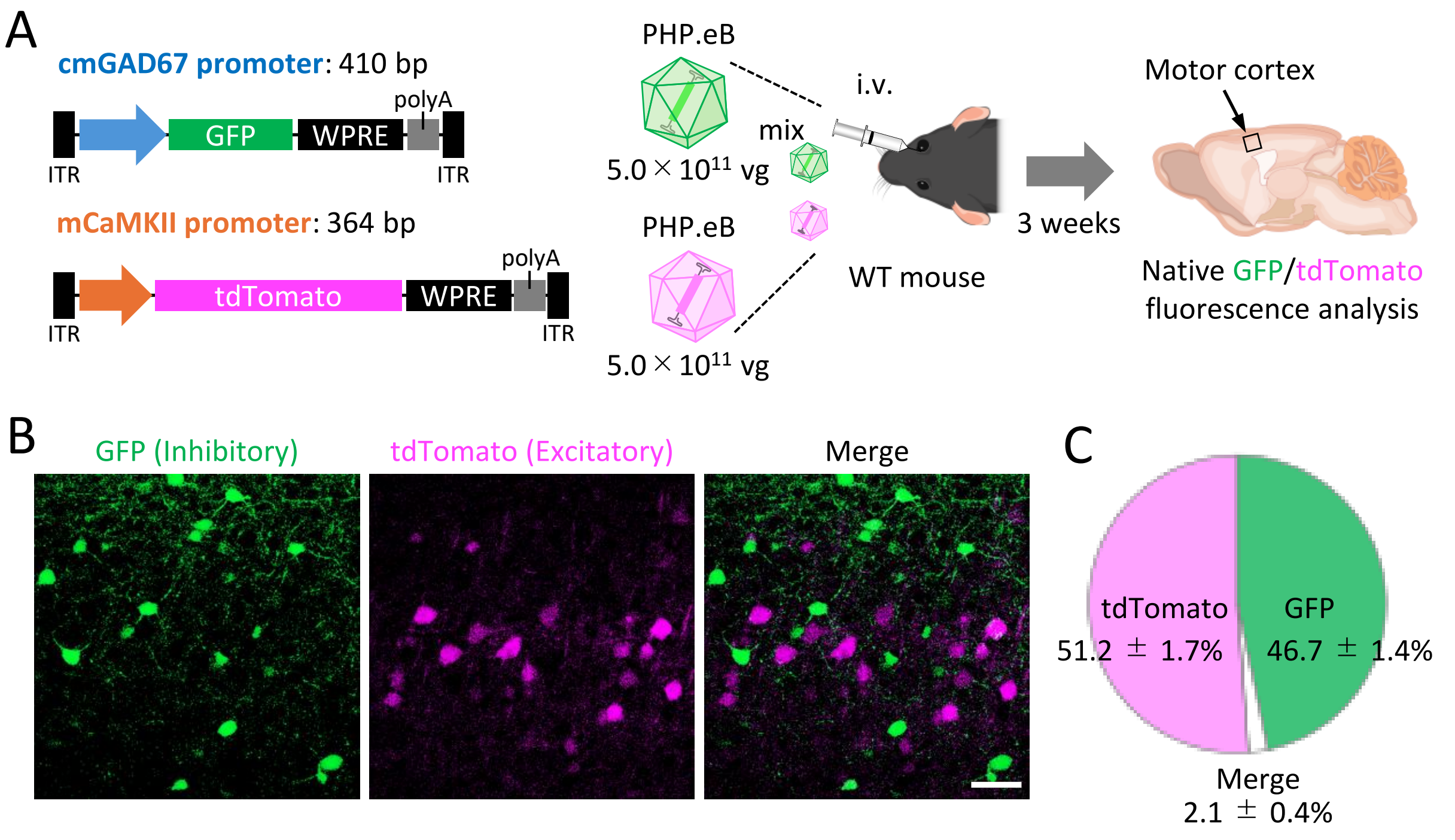

Figure S10. Complementary targeting of inhibitory and excitatory neurons using cmGAD67 and mCaMKII promoters.

(A) Schematic representation of the experimental procedure. AAV-PHP.eB expressing GFP under the control of the cmGAD67 promoter and AAV-PHP.eB expressing tdTomato under the control of the mCaMKII promoter were mixed in equal volumes (50 µL each, 1.0 × 10^{13} vg/mL) and administered via the orbital venous sinus. Three weeks post-injection, sagittal brain sections were prepared, and fluorescence microscopy was used to observe the primary motor cortex. (B) Fluorescence images of the motor cortex. Scale bar, 50 µm. (C) Pie chart showing the proportion of cells labeled with GFP alone, tdTomato alone, or both. Labeled cells were counted in the M1 region (542.5 µm × 722.4 µm) (*n* = 5 mice, 1 section/mouse). A total of 2,349 fluorescently labeled cells were counted, among which 1,094 were GFP-positive only, 1,207 were tdTomato-positive only, and 48 were co-labeled with both GFP and tdTomato.

Supplemental Tables

Statistics for Main Figures

Figure 1	F, H: one-way ANOVA Dunnett's post hoc test
Figure 3	B: one-way ANOVA Turkey's post hoc test
Figure 4	D: Mann-Whitney tests<br>G: Paired t-test
Figure 5	B: Pearson correlation<br>C (b, c), D (b,c), E (b,c): Paired t-test<br>F, G: one-way ANOVA Turkey's post hoc test<br>H: Multisample E-statistic (Energy) Test of Equal Distributions
Figure 6	B, C: one-way ANOVA Turkey's post hoc test
Figure 7	B, E: two-way ANOVA repeated measures (Group effect)<br>C, F: Gehan-Breslow-Wilcoxon test
Figure 8	B, F, G: one-way ANOVA Turkey's post hoc test<br>H: Multisample E-statistic (Energy) Test of Equal Distributions

Statistics for Supplemental Figures

Figure S3	D, E: one-way ANOVA Dunnett's post hoc test
Figure S5	E, F, G: one-way ANOVA Dunnett's post hoc test
Figure S6	B, C: Unpaired t-test
Figure S7	C, D: one-way ANOVA Turkey's post hoc test
Figure S8	C: one-way ANOVA Turkey's post hoc test
Figure S9	B, C: one-way ANOVA Turkey's post hoc test

Supplemental Video legends

Video S1. Behavior in GFP control mice after DCZ administration.
Representative behavior of a control mouse bilaterally injected into the hippocampus with AAV-PHP.eB-cmGAD67-GFP following systemic administration of DCZ. No obvious seizure-like behavior was observed.

Video S2. Seizure-like behavior induced by chemogenetic inhibition of hippocampal inhibitory neurons.
Representative behavior of a mouse bilaterally injected into the hippocampus with AAV-PHP.eB-cmGAD67-hM4D(Gi) following systemic administration of DCZ. Marked behavioral seizures were observed.

Video S3. Behavior in the DREADD-only group after DCZ administration.
Representative behavior of a mouse receiving excitatory neuron-specific Gq-DREADD in the motor cortex after administration of DCZ. Moderate seizures were induced in the absence of additional AAV treatment.

Video S4. Behavior in the GFP control group after DCZ administration.
Representative behavior of a mouse co-injected with excitatory neuron-specific Gq-DREADD and control AAV-GFP in the motor cortex after administration of DCZ. Moderate seizures comparable to those in the DREADD-only group were observed.

Video S5. Behavior in the GAD65 treatment group after DCZ administration.
Representative behavior of a mouse co-injected with excitatory neuron-specific Gq-DREADD and AAV-cmGAD67-GAD65 in the motor cortex after administration of DCZ. Seizure behavior was markedly reduced compared with the DREADD-only and GFP control groups.

Supplemental methods

Promoter construct generation

Stepwise truncations and deletions of the *Gad1* locus were performed to identify regulatory regions including Ri, Rii, and putative core promoter. Constructs were cloned into AAV-PHP.eB backbones driving GFP expression for in vivo analysis (Supplemental figure S1, S3).

RT-qPCR quantification

The mRNA measurements in supplemental figure S7C and S7D related to figure 3 were done by quantitative RT-PCR. Cortical or hippocampal samples were homogenized in TRIzol Reagent (Thermo Fisher Scientific, MA, USA) and frozen in liquid nitrogen. The total RNA was extracted by RNeasy Lipid Tissue Mini Kit (Qiagen, Hilden, Germany) and cDNA was prepared by ReverTra Ace reverse transcriptase (Toyobo, Osaka, Japan). The amount of mGAD65 mRNA was determined using THUNDERBIRD SYBR qPCR Mix (Toyobo) with real-time PCR TP970 (TaKaRa Bio, Shiga, Japan). Relative expression levels were normalized to β-actin.

The primers are as follows.

mGAD65: 5'-ACT ACT GGG TTT GAG GCA CA-3' (Forward) and 5'-TGC GCA AAC TAG GAG GTA CA-3' (Reverse)

β-actin: 5'-ACC AAC TGG GAC GAT ATG GAG AAG A-3' (Forward) and 5'-TAC GAC CAG AGG CAT ACA GGG ACA A-3' (Reverse)

Immunohistochemistry

The immunostaining shown in supplemental figure S6 were performed using the same procedures as those described for Figure 3.

The following primary and secondary antibodies were used:

・Mouse anti-NeuN (1:1,000; MAB377, Millipore, MA, USA)

・Alexa Fluor 647-conjugated donkey anti-mouse IgG (1:2,000; A32787, Invitrogen)

GABA quantification

Three weeks after injection of AAV vector or PBS, cortical (near M1) and hippocampal tissues were collected. GABA concentrations were quantified using a Mouse GABA enzyme-linked immuno sorbent assay (ELISA) Kit (ab287793; Abcam) according to the manufacturer's instructions. Brain tissue was homogenized in PBS, and total protein concentrations were measured using the Micro BCA™ Protein Assay Kit (Thermo Fisher Scientific). Samples were diluted to equal protein concentrations prior to ELISA. Absorbance values were measured using an Infinite M Nano microplate reader (Tecan, Männedorf, Switzerland). Values were normalized to protein concentration in each sample.

Behavioral paradigms

Locomotor Activity Monitoring:

Infrared beam breaks were recorded over a 24-hour period using the Locomotor Activity Measuring System (LS-5; MELQUEST, Tokyo, Japan) to assess circadian activity. Mouse activity was monitored continuously throughout the day, starting at 12:00 noon, using infrared light emitted from the device.

Rotarod and Beam Tests:

Motor coordination was evaluated using the rotarod latency to fall and beam traversal time based on previous report [2]. Three weeks after AAV or PBS administration, rotarod performance was assessed using a treadmill device (MK-610A/RKZ; Muromachi Kikai).) Each mouse was tested three times at 30-minute intervals. The rotational speed was gradually increased from 4 rpm to 50 rpm over 300 seconds, and the time to fall from the rod was recorded. For the beam test, mice were placed at one end of a narrow bar (800 mm long, 11 mm in diameter and allowed to walk toward a goal box located at the opposite end. This task was repeated three times, and the average time required to traverse the beam was recorded as a measure of motor coordination.

Supplemental References

1. Hoshino, C., Konno, A., Hosoi, N., Kaneko, R., Mukai, R., Nakai, J., and Hirai, H. (2021). GABAergic neuron-specific whole-brain transduction by AAV-PHP.B incorporated with a new GAD65 promoter. Mol Brain *14*, 33. 10.1186/s13041-021-00746-1.
2. Okada, Y., Hosoi, N., Matsuzaki, Y., Fukai, Y., Hiraga, A., Nakai, J., Nitta, K., Shinohara, Y., Konno, A., and Hirai, H. (2022). Development of microglia-targeting adeno-associated viral vectors as tools to study microglial behavior in vivo. Commun Biol *5*, 1224. 10.1038/s42003-022-04200-3.
