# Supplementary material for: A compact GAD67 promoter enables inhibitory neuron-targeted AAV gene therapy for seizure suppression
Source: Mol Ther. 2026 Jun 25;34(9):5510–26. doi: 10.1016/j.ymthe.2026.06.007 (PMC13555566; doi:10.1016/j.ymthe.2026.06.007)
Supplement: Supplementary file 2 [file mmc7.pdf]

Molecular Therapy
Original Article

A compact GAD67 promoter enables inhibitory neuron-targeted AAV gene therapy for seizure suppression

Yuuki Fukai,[1,2] Ayumu Konno,[1,2] Nobutake Hosoi,[2] Karin Miyakawa,[2] Ryosuke Kaneko,[3] and Hirokazu Hirai[1,2]

[1]Viral Vector Core, Gunma University, Initiative for Advanced Research, Maebashi, Gunma 371-8511, Japan; [2]Department of Neurophysiology & Neural Repair, Gunma University Graduate School of Medicine, Maebashi, Gunma 371-8511, Japan; [3]Medical Genetics Research Center, Nara Medical University, 840 Shijo-Cho, Kashihara, Nara 634-8521, Japan

Epilepsy arises from disruption of excitation-inhibition (E/I) balance, typically due to excessive excitatory activity. Despite available therapies, a substantial proportion of patients remain treatment resistant. Enhancing inhibitory neuron activity via gene therapy can restore E/I balance and may therefore provide a therapeutic strategy for treatment-resistant epilepsy. Here, we developed a compact 410-bp glutamic acid decarboxylase 67 promoter (cmGAD67) that enables strong, selective transgene expression in inhibitory neurons while preserving adeno-associated virus (AAV) packaging capacity. Systemic delivery of AAV vectors carrying cmGAD67 preferentially targeted parvalbumin interneurons and enabled efficient circuit modulation. To evaluate therapeutic potential, we expressed glutamic acid decarboxylase 65 (GAD65) under the control of cmGAD67 (AAV-GAD65). AAV-GAD65 suppressed abnormal delta oscillations, reduced seizure-like activity, normalized anxiety-like behavior, and improved survival in seizure models. Biochemical analyses confirmed increased GABA levels in the cortex and hippocampus, linking functional improvements to enhanced inhibitory neurotransmitter synthesis. Together, these findings establish the cmGAD67 promoter as a versatile platform for inhibitory neuron-targeted AAV gene delivery and identify AAV-GAD65 as a promising strategy for seizure control and disorders associated with E/I imbalance.

INTRODUCTION

The maintenance of excitation-inhibition (E/I) balance is essential for higher brain functions such as cognition, behavior, and learning. In the cerebral cortex, the activity of excitatory pyramidal neurons is tightly regulated by inhibitory interneurons. Impairment of inhibitory neuron function shifts the E/I balance toward excitation, a pathological state implicated in the development of diverse neuropsychiatric disorders, including epilepsy, anxiety disorders, autism spectrum disorders, and schizophrenia.[1–3] Thus, strategies that specifically modulate inhibitory neuron function hold promise for the treatment of these conditions.

GABAergic interneurons in the cortex and hippocampus release γ-aminobutyric acid (GABA), the principal inhibitory neurotransmitter, which suppresses excitatory signaling through activation of postsynaptic GABA receptors. GABA is synthesized from glutamate by glutamic acid decarboxylase (GAD), an enzyme expressed selectively in inhibitory neurons. Two isoforms exist, GAD65 (encoded by *Gad2*) and GAD67 (encoded by *Gad1*).[4] GAD65 is localized mainly to synaptic vesicles and contributes to activity-dependent GABA synthesis, whereas GAD67 is distributed throughout the cytoplasm and is thought to maintain basal GABA levels in neurons.[4–6] Consistent with these roles, GAD65-deficient mice are viable, whereas GAD67-deficient mice die shortly after birth,[7,8] underscoring the essential role of GAD67 in maintaining basal GABA levels required for normal neuronal and circuit function.

Previously, we identified a 2.5-kb fragment upstream of exon 1 of the *Gad2* gene that functions as an inhibitory neuron-specific promoter, designated mGAD65(delE1),[9] which has been distributed to multiple laboratories (Addgene #177316). Although this promoter can be incorporated into adeno-associated virus (AAV) vectors, its large size occupies more than half of the limited AAV packaging capacity (∼4.7 kb), and its weak activity limits the expression of therapeutic genes, particularly in settings where vector copy number per cell is low, such as after systemic delivery.

To overcome these limitations, we conducted a systematic analysis of the *Gad1* locus and identified a compact 410-bp sequence, termed the compact mouse GAD67 (cmGAD67) promoter. Despite its short length, the cmGAD67 promoter exhibits strong activity and high inhibitory neuron specificity when packaged into AAV vectors. In this study, we used cmGAD67-driven AAV vectors to manipulate

Received 19 November 2025; accepted 2 June 2026;
https://doi.org/10.1016/j.ymthe.2026.06.007.

Correspondence: Ayumu Konno, Viral Vector Core, Gunma University, Initiative for Advanced Research, Maebashi, Gunma 371-8511, Japan.
E-mail: konnoa@gunma-u.ac.jp
Correspondence: Hirokazu Hirai, Viral Vector Core, Gunma University, Initiative for Advanced Research, Maebashi, Gunma 371-8511, Japan .
E-mail: hirai@gunma-u.ac.jp

 © 2026 The Author(s).
Published by Elsevier Inc. on behalf of The American Society of Gene and Cell Therapy.
This is an open access article under the CC BY license (http://creativecommons.org/licenses/by/4.0/).

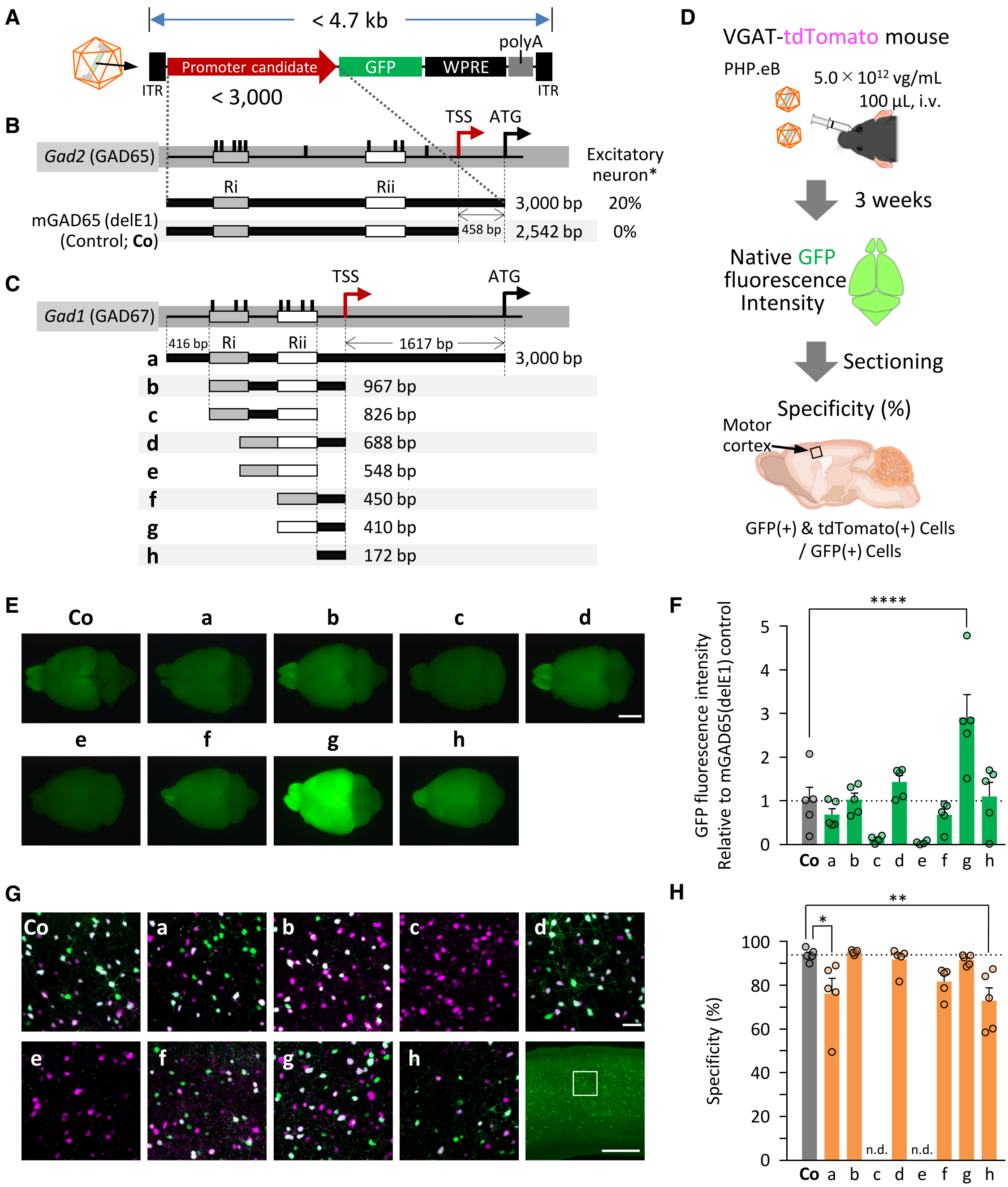

Figure 1. Characterization of cmGAD67

(A) Schematic diagram of the AAV genome used in this study. When GFP, WPRE, and SV40 poly(A) are included between the ITRs, the promoter sequence must be shorter than 3 kb to remain within the AAV packaging limit (∼4.7 kb). (B and C) Schematic representation of the *Gad2* (B) and *Gad1* (C) loci and promoter candidates. Vertical bars indicate binding motifs for the inhibitory neuron-specific transcription factor Dlx. Gray (region i [Ri]) and white (region ii [Rii]) boxes represent regions enriched in Dlx-binding motifs. Promoter candidates were initially cloned from ∼3 kb upstream of the ATG start codon and subsequently truncated based on the transcription start site (TSS), retaining either or both Ri and Rii regions. The 2,542-bp region upstream of the TSS in (B) corresponds to the previously reported inhibitory neuron-specific mGAD65(delE1) promoter, which was used as a control (Co). Promoter candidates derived from the *Gad1* locus (C, a–d) were evaluated. (D) Experimental design. AAV-PHP.eB vectors expressing GFP under the control of *Gad1*-derived promoter candidates (100 μL, 5.0×10^{11} vg/mouse) were intravenously administered via the retro-orbital sinus into VGAT-tdTomato mice, in which inhibitory neurons express the red fluorescent protein tdTomato.[12] Three weeks after injection, mice were perfused, and GFP fluorescence on the dorsal surface of the whole brain was imaged to assess promoter activity. Sagittal sections were then prepared, and inhibitory neuron specificity of GFP expression was quantified in the M1/M2 motor cortex. (E) Representative fluorescence images of whole brains from VGAT-tdTomato mice injected with AAV-PHP.eB expressing GFP under

(legend continued on next page)

inhibitory neuron function and evaluate their effects on brain activity, behavior, and seizure outcomes.

Importantly, the clinical feasibility of GAD65-based therapy has been demonstrated in humans. A first-in-human clinical trial reported that AAV-mediated GAD65 delivery to the subthalamic nucleus was safe and showed therapeutic potential in patients with Parkinson's disease.[10] Although this approach targeted a different brain region and disease context, it supports the translational potential of GAD65-based strategies. Building on this precedent, we highlight the potential of cmGAD67-driven AAV-GAD65 as a promising gene-therapy approach for epilepsy.

RESULTS

Development of compact GAD67 and compact GAD65 promoters

The transcription factor Dlx directly binds to the *Gad1* and *Gad2* loci and regulates GAD gene expression.[11] Within the *Gad1* and *Gad2* loci, two regions enriched in Dlx-binding motifs (TAAT/ATTA), termed region i (Ri) and region ii (Rii), have been identified.[11] These regions are thought to function as enhancers in inhibitory neurons and play important roles in both GABAergic neuron specificity and promoter activity.

In a previous study, we identified a 2,542-bp inhibitory neuron-specific promoter (mGAD65(delE1)) within the upstream regulatory region of *Gad2* containing the Ri and Rii elements.[9] Notably, inclusion of the 458-bp region between the transcription start site (TSS) and ATG resulted in increased expression in excitatory neurons and reduced specificity to approximately 80% (Figure 1B). Because Ri and Rii are located within 1 kb upstream of the TSS in the *Gad1* locus (Figure 1C), we hypothesized that a shorter inhibitory neuron-specific promoter could be derived from this region. We have previously identified cell-type-specific promoters for Purkinje cells[13] and microglia,[14,15] both derived from regions 1–2 kb upstream of the ATG. Based on this strategy, we initiated a systematic truncation analysis of the *Gad1* locus, starting from approximately 3 kb upstream of the ATG—corresponding to the upper size limit compatible with AAV vectors carrying GFP and woodchuck hepatitis virus post-transcriptional regulatory element (WPRE)—and progressively shortened the sequence to identify the minimal region that retains both high specificity for inhibitory neurons and strong promoter activity.

For promoter truncation, we used the structure of the mGAD65(delE1) promoter as a reference and defined the TSS as the anchor point. Regions lacking dense Dlx-binding motifs outside Ri and Rii were first removed, followed by selective deletion of Ri, Rii, or both, in order to determine the regions critical for inhibitory neuron specificity and promoter activity (Figure 1C).

AAV-PHP.eB vectors[16] expressing GFP under the control of mGAD65(delE1) (control [Co]) or candidate *Gad1*-derived promoter fragments (a–h) were generated and intravenously administered via the retro-orbital sinus into VGAT-tdTomato mice,[12] in which inhibitory neurons are labeled with tdTomato (100 μL, 5.0×10^{11} vector genomes (vg)/mouse) (Figure 1D). Three weeks after injection, mice were perfused, and GFP fluorescence across the dorsal surface of the whole brain was imaged using a stereomicroscope. Sagittal sections were then prepared, and inhibitory neuron specificity of GFP expression was evaluated in the M1/M2 motor cortex. Whole-brain GFP fluorescence was significantly higher in construct (g) compared with the control (****$p < 0.0001$) (Figures 1E, 1F, and S1). The inhibitory neuron specificity of AAV carrying construct (g) was 92.2% ± 1.3% (n = 5), comparable to that of the control (Figures 1G and 1H).

Deletion of the 172-bp region between the 3′ end of Rii and the TSS (constructs c and e) abolished promoter activity, whereas the 172-bp fragment alone (construct h) retained promoter activity but exhibited significantly reduced inhibitory neuron specificity (**$p < 0.01$) (Figure 1H). These findings indicate that this 172-bp region functions as a core promoter. Furthermore, addition of Rii to this core promoter (construct g) significantly enhanced both promoter activity and inhibitory neuron specificity, indicating that Rii functions as an inhibitory neuron-specific enhancer. Together, these results indicate that a 410-bp region consisting of the core promoter and the Rii enhancer (Figure 1C(g); sequence shown in Figure S2) is necessary and sufficient to drive strong and selective expression in inhibitory neurons. We therefore designated this sequence as the compact mouse GAD67 (cmGAD67) promoter.

We next re-evaluated the *Gad2* (GAD65) locus and found that the TSS differs by 123 bp between the genome database used in our previous study (ENSMUST00000028123_4) and that used by Eisenstat et al. (NM_008078.2),[11] which identified Ri and Rii (Figure S3B). Using the NM_008078.2 TSS as a reference, we performed a similar truncation analysis of the *Gad2* promoter region (Figures S3B and S3C). As observed for *Gad1*, the 123-bp region between the 3′ end of Rii and the TSS functions as a core promoter, while Rii acts as an inhibitory neuron-specific enhancer (Figures S3D and S3E). Based on these findings, we identified a

the control of mGAD65(delE1) (Co) or *Gad1*-derived promoter candidates (a–d). Scale bar, 5 mm. (F) Quantification of dorsal whole-brain GFP fluorescence intensity. Fluorescence intensity in the mGAD65(delE1) control group was normalized to 1. (G) Representative fluorescence images of GFP and tdTomato (inhibitory neurons) in the motor cortex (M1/M2). The inset (lower right) shows a low-magnification image indicating the analyzed cortical region (boxed area). Scale bars, 50 μm (high magnification) and 500 μm (low magnification). (H) Quantification of the proportion of GFP-positive cells that were also tdTomato positive (indicative of inhibitory neuron specificity). Each dot represents an individual mouse. *$p < 0.05$, **$p < 0.01$ and ****$p < 0.0001$ by one-way ANOVA with Dunnett's *post hoc* test. n.d., not determined.

486-bp region comprising both elements (Figure S3B(f)) and designated it as the compact mouse GAD65 (cmGAD65) promoter.

Preferential targeting of PV-positive interneurons by the cmGAD67 and cmGAD65 promoters

To further characterize the neuronal subtypes transduced by the cmGAD67 and cmGAD65 promoters, we examined their expression in parvalbumin (PV)- and somatostatin (SST)-positive interneurons (PV^+ and SST^+), which represent two major classes of cortical inhibitory neurons. Quantitative analyses were performed in the primary and secondary motor cortex (M1/M2), a well-defined cortical region suitable for assessing neuronal subtype specificity. The same regions were analyzed in our previous study, in which we identified the mGAD65(delE1) promoter,[9] enabling direct comparison of promoter performance.

AAV-PHP.eB vectors expressing GFP under the control of either the cmGAD67 or cmGAD65 promoter were intravenously administered to mice (100 μL, 5.0×10^{11} vg/mouse), and, 3 weeks later, brain sections were immunostained using antibodies against PV and SST (Figures 2A–2C). Both cmGAD67 and cmGAD65 promoters showed the highest level of GFP expression in PV^+ interneurons. Approximately 85% of PV^+ neurons were GFP positive (light blue bars in Figure 2D), and more than 60% of GFP-expressing cells were PV^+ interneurons (light blue bars and pie chart segments in Figure 2E). In contrast, fewer than 50% of SST^+ neurons were GFP positive (pink bars in Figure 2D), and SST^+ neurons accounted for only 10–15% of all GFP-positive cells (pink bars and pie chart segments in Figure 2E). In addition, inhibitory neuron subtypes other than PV^+ and SST^+ (e.g., VIP^+ interneurons) comprised approximately 25% of GFP-positive cells (gray bars and pie chart segments in Figure 2E).

We next normalized GFP-positive subtypes to their relative abundance in the cortex (40% PV^+, 15% SST^+, and 45% other interneurons).[9] A value close to 1 indicates no subtype preference, whereas values greater than 1.5 for PV^+ neurons indicate preferential expression in this subtype. Both cmGAD67 and cmGAD65 promoters showed clear enrichment in PV^+ interneurons (Figure 2F). These results indicate preferential expression in PV^+ interneurons, a property that is particularly advantageous for modulating excitatory output through perisomatic inhibition.[17,18]

This inhibitory neuron specificity was consistently observed across multiple brain regions, including the cortex, striatum, hippocampus, and cerebellum (Figure S4).

The cmGAD67 promoter exhibits significantly higher transcriptional activity than cmGAD65 promoter

To compare promoter strength, AAV-PHP.eB vectors expressing GFP under the control of the previously reported mGAD65(delE1), cmGAD65, or cmGAD67 promoters were administered via the retro-orbital sinus (100 μL, 5.0×10^{11} vg/mouse) (Figure 3A). Three weeks after injection, mRNA was isolated from the M1/M2 motor cortex, and GFP expression levels were quantified by reverse transcription quantitative PCR (RT-qPCR) (Figure 3B). When normalized to AAV-mGAD65(delE1) (set to 1.0 ± 0.2, n = 6 mice), relative expression levels were 0.6 ± 0.1 for AAV-cmGAD65 (n = 6 mice) and 1.8 ± 0.1 for AAV-cmGAD67 (n = 6 mice). Compared with AAV-mGAD65(delE1), no significant difference was observed for AAV-cmGAD65 (p = 0.2134), whereas AAV-cmGAD67 exhibited significantly higher expression (p = 0.013 vs. mGAD65(delE1); p = 0.0004 vs. cmGAD65). Based on these results, cmGAD67 was selected for subsequent experiments.

Consistent with these findings, comparative whole-brain fluorescence analysis further confirmed that the cmGAD67 promoter exhibited stronger overall expression than cmGAD65 while maintaining high inhibitory neuron specificity comparable to the conventional mGAD65(delE1) promoter (Figure S5).

Application of the cmGAD67 promoter for optogenetic and chemogenetic manipulation

We next examined whether the cmGAD67 promoter could be used to bidirectionally manipulate inhibitory neuron function through optogenetics and chemogenetics. For neuronal activation, we employed GFP-fused channelrhodopsin-2 (ChR2), and, for inhibition, GFP-fused Gi-Designer Receptors Exclusively Activated by Designer Drugs (DREADD) was used (Figure 4A). AAV-PHP.eB-cmGAD67-ChR2 (100 μL, 5.0×10^{11} vg/mouse) was intravenously administered to VGAT-tdTomato mice,[12] and patch-clamp recordings were performed in cortical slices 3 weeks later. In contrast, AAV-PHP.eB-cmGAD67-hM4D(Gi) (Gi-DREADD) was injected bilaterally into the hippocampus of wild-type mice, and, 3 weeks later, behavioral seizures and electrocorticogram (ECoG) recordings from the motor cortex were assessed.

Blue-light stimulation induced no response in GFP-negative inhibitory neurons, whereas ChR2-expressing neurons showed robust photocurrents and action potentials (Figures 4B–4D). Following intravenous administration of AAV-ChR2, inhibitory neuron specificity in the cortex was 94.5% ± 1.9% (n = 5).

For chemogenetic manipulation, AAV-PHP.eB-cmGAD67-hM4D (Gi) or control AAV-PHP.eB-cmGAD67-GFP (5 μL, 1.0×10^{11} vg/mouse) was injected into the bilateral hippocampus of mice. GFP expression from AAV-PHP.eB-cmGAD67-hM4D(Gi) was predominantly observed in GABA-positive inhibitory neurons, as confirmed by immunostaining (Figures 4E and 4F, top). In control mice injected with AAV-PHP.eB-cmGAD67-GFP, systemic administration of deschloroclozapine (DCZ) produced no behavioral or electrophysiological effects (Video S1 and Figure 4E). In contrast, Gi-DREADD-expressing mice exhibited marked behavioral seizures (Video S2). Correspondingly, ECoG recordings revealed epileptiform activity characterized by abnormal spike-and-wave discharges with amplitudes exceeding 300 μV following DCZ administration in

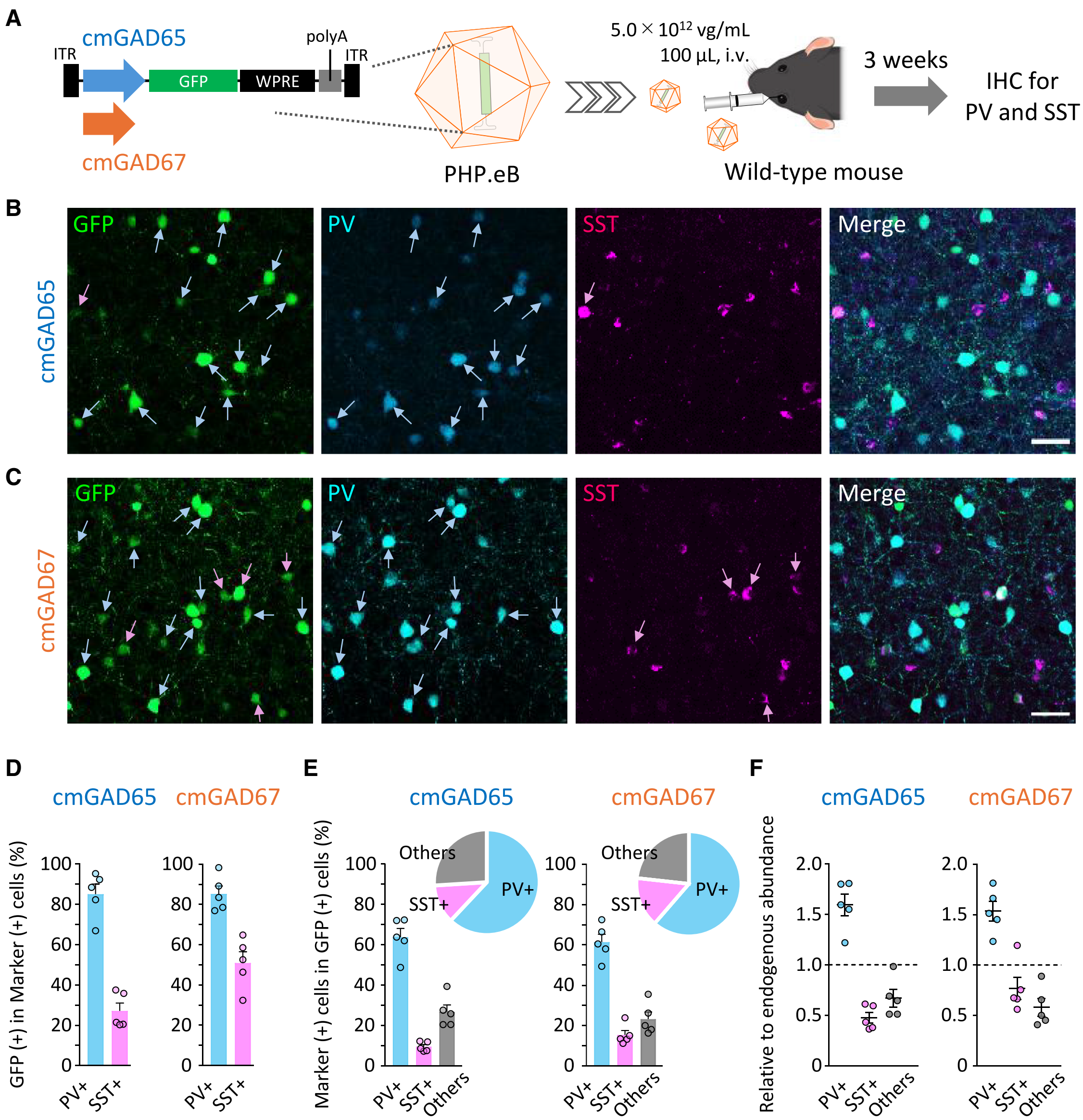

Figure 2. Subtype specificity of cmGAD65 and cmGAD67 promoters in PV-positive and SST-positive interneurons

(A) Schematic of the experimental procedure. AAV-PHP.eB vectors expressing GFP under the control of the cmGAD65 or cmGAD67 promoter (100 μL, 5.0 × 10^{11} vg/mouse) were intravenously injected into wild-type mice. Three weeks later, sagittal brain sections were prepared and subjected to immunohistochemistry (IHC). (B and C) Immunohistochemical staining of the motor cortex for parvalbumin (PV, blue channel) and somatostatin (SST, magenta channel) in mice injected with PHP.eB carrying the cmGAD65 promoter (B) or cmGAD67 promoter (C). Cyan arrows indicate PV-positive (PV^+) neurons, and magenta arrows indicate SST-positive (SST^+) neurons. Scale bars, 50 μm. (D) Quantification of the proportion of GFP-labeled cells within PV^+ and SST^+ neuronal populations. Each dot represents data from an individual mouse. (E) Bar graphs and pie charts showing the composition of PV^+, SST^+, and double-negative cells (others) among GFP-expressing cells. As both cmGAD65 and cmGAD67 promoters exhibit ~95% specificity for inhibitory neurons, the "others" category is primarily composed of inhibitory neurons other than PV^+ and SST^+ cells, such as VIP^+ neurons. (F) Relative tropism of the cmGAD promoter-driven AAV-PHP.eB vectors for different GABAergic neuron subtypes (PV^+, SST^+, or others) in the mouse cortex. In the mouse cortex, PV^+ neurons account for 40%, SST^+ neurons for 15%, and other subtypes for 45% of all GABAergic neurons.[9] The ratio of each GFP-expressing subtype (from E) was divided by its expected proportion in the cortex to calculate relative enrichment. A value near 1 indicates no subtype preference, whereas a value greater than 1.5 for PV^+ neurons indicates selective gene expression in this subtype.

Gi-DREADD mice (Figure 4F). These events were used as a measure of abnormal spike activity and were further characterized in subsequent analyses.

Delta-band (0.5–4 Hz) power was selected as a primary readout because slow-wave activity is a well-established hallmark of cortical hyperexcitability and seizure-related network dysfunction.[19,20] In

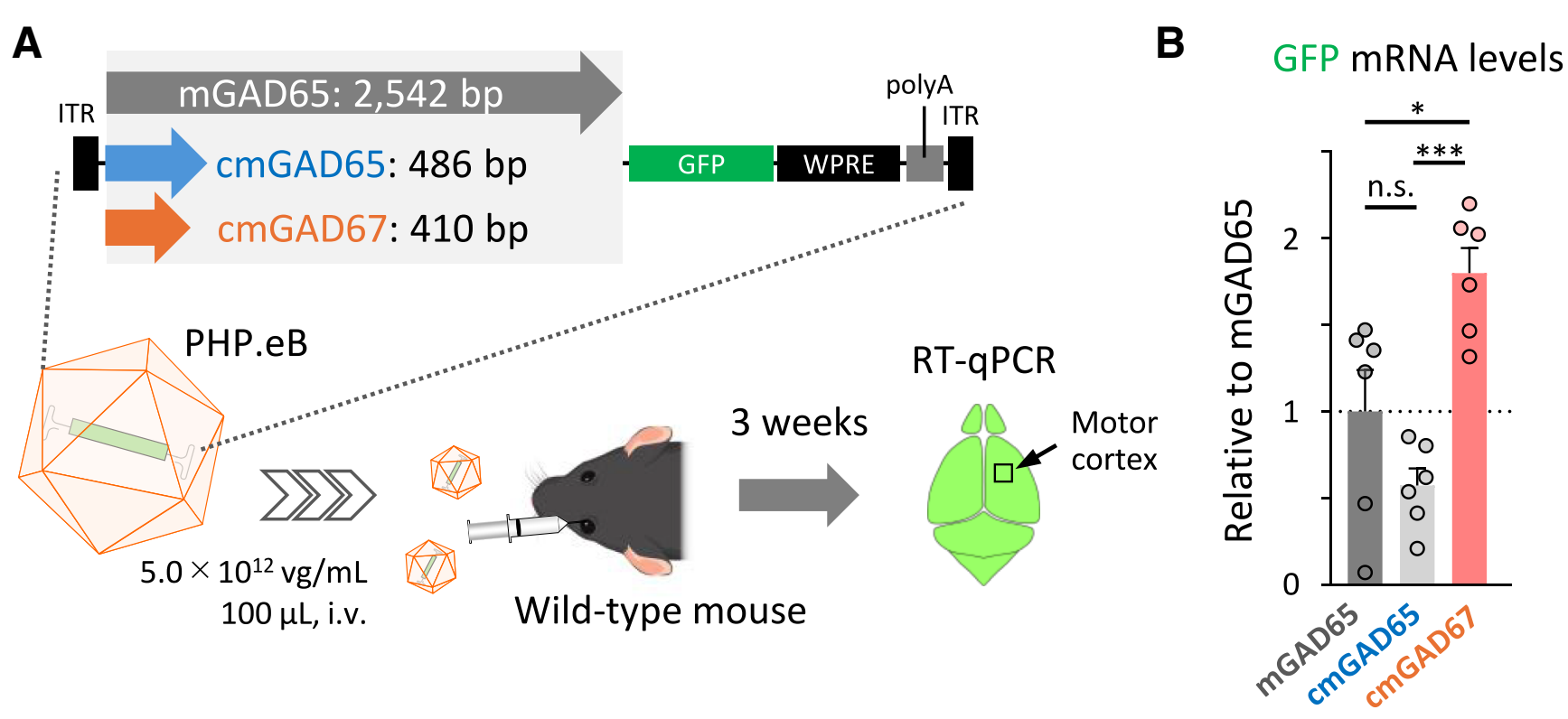

Figure 3. Comparative analysis of promoter strength reveals enhanced activity of cmGAD67 *in vivo*

(A) Schematic representation of AAV-PHP.eB vectors expressing GFP under the control of the previously reported mGAD65(delE1), cmGAD65, or cmGAD67 promoters. Vectors (100 μL, 5.0 × 10^{11} vg/mouse) were administered via the retro-orbital sinus. (B) Quantification of GFP mRNA expression levels in the M1/M2 motor cortex by RT-qPCR 3 weeks after injection. Expression levels were normalized to those of AAV-mGAD65(delE1). Data are presented as mean ± SEM (mGAD65(delE1), n = 6 mice; cmGAD65, n = 6 mice; cmGAD67, n = 6 mice). No significant difference was observed between mGAD65(delE1) and cmGAD65 (p = 0.213), whereas cmGAD67 showed significantly higher expression compared with both mGAD65(delE1) (p = 0.013) and cmGAD65 (p = 0.0004). Each dot represents an individual mouse. *p < 0.05; ***p < 0.001; n.s., not significant (one-way ANOVA with Tukey's test).

our recordings, delta power showed the most robust and consistent changes across animals and experimental conditions, whereas other frequency bands exhibited smaller or less reproducible alterations (data not shown). Quantification of abnormal spike events (between 300 and 1,000 μV) and delta-band power during 10–25 min after DCZ administration demonstrated a significant increase in the Gi-DREADD group, with no such increase observed in GFP controls (Figure 4G).

In contrast to the intravenous administration, direct hippocampal injection of AAV-GFP or AAV-hM4D(Gi) resulted in reduced specificity (63.9% ± 4.1% and 61.4% ± 0.9%, respectively; n = 5). These findings indicate that, while the inhibitory neuron specificity of the cmGAD67 promoter is largely independent of the transgene, it is influenced by the route of administration, with reduced specificity observed following direct parenchymal injection. This reduction may be attributable to high local vector concentrations.

AAV-GAD65 suppresses epileptiform activity in the pentylenetetrazol kindling model

We next examined the effects of AAV-cmGAD67-GAD65 (hereafter AAV-GAD65) on epileptiform activity in the pentylenetetrazol (PTZ) kindling model. Repeated intraperitoneal injections of PTZ (30 mg/kg) resulted in progressive increases in seizure severity as assessed by the Racine scale (Figures 5A and 5B), confirming successful induction of chronic seizure susceptibility.

ECoG recordings obtained 1 and 22 days after the final PTZ injection (Figure 5A) revealed persistent large discharges exceeding 300 μV in PTZ-treated control mice (Figure 5C), as observed in DCZ-treated mice expressing Gi-DREADD in hippocampal inhibitory neurons (Figures 4F and 4G). However, waveforms exceeding 1,000 μV were considered to be motion artifacts[21,22] and were therefore excluded from subsequent analyses. Spikes between 300 and 1,000 μV were rare in naive mice but markedly increased (>30-fold) in PTZ-treated mice and were therefore defined as seizure-like events. Although repeated PTZ administration increased seizure susceptibility, overt seizures detectable by the Racine scale were not observed after completion of the 11 PTZ injection sessions.

Intravenous administration of AAV-GAD65 (100 μL, 5.0 × 10^{11} vg/mouse) (Figure 5E), but not the same dose of AAV-cmGAD67-GFP (AAV-GFP) (Figure 5D), 2 days after the final PTZ injection markedly improved cortical network activity. Both the number of seizure-like events and delta power were greatly reduced by AAV-GAD65 at 22 days (Figure 5E (b and c)). Importantly, neither the proportion of inhibitory neurons nor the cell-type specificity of AAV-cmGAD67–mediated expression was altered by PTZ treatment, confirming that the observed effects are not attributable to changes in cellular composition or targeting specificity (Figure S6). Figures 5F and 5G summarize the number of seizure-like events and delta power at 1 and 22 days after the 11th PTZ injection in the control (PTZ only), PTZ + AAV-GFP, and PTZ + AAV-GAD65 groups. In the AAV-GAD65-injected group, both parameters were markedly decreased, and delta power was significantly reduced to the level observed in naive mice without PTZ treatment.

To further evaluate the relationship between seizure-like events and slow-wave activity, we performed a group-level analysis of seizure-like events versus delta power (Figure 5H). In PTZ-treated control mice and those receiving AAV-GFP, seizure-like events were prominent and delta power was elevated 1 day after the final PTZ injection, and both measures remained persistently abnormal at 22 days. No significant recovery was observed between the two time points, and both groups continued to differ significantly even at 22 days from naive mice (light blue circles in Figure 5H). In contrast, mice treated with AAV-GAD65 exhibited a distinct trajectory. Although seizure-like events and delta power were similarly elevated 1 day after PTZ treatment, both measures

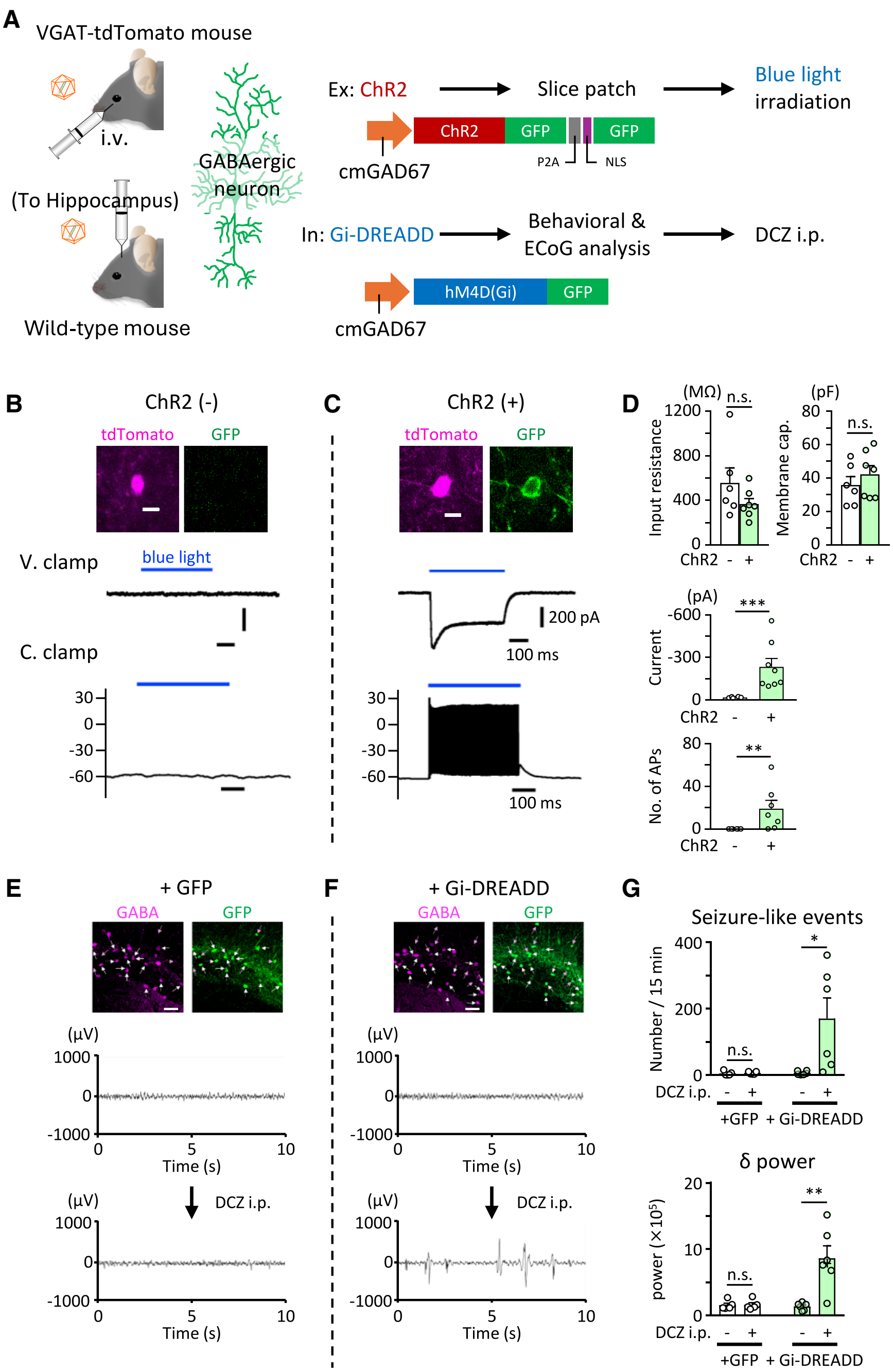

Figure 4. Application of the cmGAD67 promoter for optogenetic and chemogenetic manipulation of inhibitory neurons

(A) Schematic diagrams of AAV constructs expressing channelrhodopsin-2 (ChR2) or the inhibitory designer receptor hM4Di (Gi-DREADD) under the control of the cmGAD67 promoter. (B and C) Representative recordings from cortical tdTomato-positive inhibitory neurons without (B) or with (C) ChR2 expression under voltage-clamp (V. clamp) and current-clamp (C. clamp) conditions, showing robust light-evoked responses only in ChR2(+) inhibitory neurons. Scale bars, 10 μm. (D) Pooled data demonstrate no change in passive membrane properties but significant increases in light-evoked inward current and action potential (AP) firing in inhibitory neurons following optical stimulation (n = 6–8 mice; $^{**}p < 0.01$, $^{***}p < 0.001$, Mann-Whitney test; mean ± SEM). (E and F) Representative ECoG traces from mice injected with AAV-cmGAD67-GFP or AAV-cmGAD67-hM4Di-GFP, showing increased network activity only in Gi-DREADD-expressing mice following systemic administration of DCZ. Scale bars, 50 μm. (G) Quantification of ECoG traces indicate a significant increase in the number of abnormal spikes (epileptiform discharges) and in delta power after chemogenetic inhibition (n = 5–6 mice; $^{*}p < 0.05$, $^{**}p < 0.01$, paired t test; mean ± SEM).

showed significant recovery by 22 days. Importantly, values in the AAV-GAD65 group at 22 days were no longer significantly different from those of naive mice (p = 0.23, right graph in Figure 5H). These results indicate that, while control and AAV-GFP groups failed to recover from PTZ-induced cortical hyperexcitability, AAV-GAD65 treatment resulted in a normalization of network activity. Although seizure-related events alone did not show a statistically significant reduction (Figure 5F), a combined analysis of seizure-related events and delta power revealed a significant shift toward naive-like distributions using the energy distance test (Figure 5H).

We evaluated the inhibitory neuron specificity of intravenously delivered AAV-GAD65 in the M1/M2 motor cortex and hippocampus. AAV-GAD65 exhibited high specificity across regions, with 92.4% ± 0.3% in the cortex, and 93.2% ± 0.4% and 86.5% ± 1.9% in the hippocampal CA1 and CA3 regions, respectively (n = 5 for all groups). To directly assess whether AAV-GAD65 enhances GABA production, we measured tissue GABA levels in the cortex and hippocampus. Biochemical assays revealed that AAV-GAD65 significantly elevated GABA content compared with control groups (Figure S7), confirming that transgene expression increased inhibitory neurotransmitter availability. These results provide mechanistic support for the observed suppression of epileptiform activity.

AAV-GAD65 alleviates anxiety-like behavior in PTZ-kindled mice

We next investigated whether intravenous administration of AAV-GAD65 (100 μL, 5.0 × 10^{11} vg/mouse) could ameliorate anxiety-like behavior associated with PTZ kindling. In the open-field test,

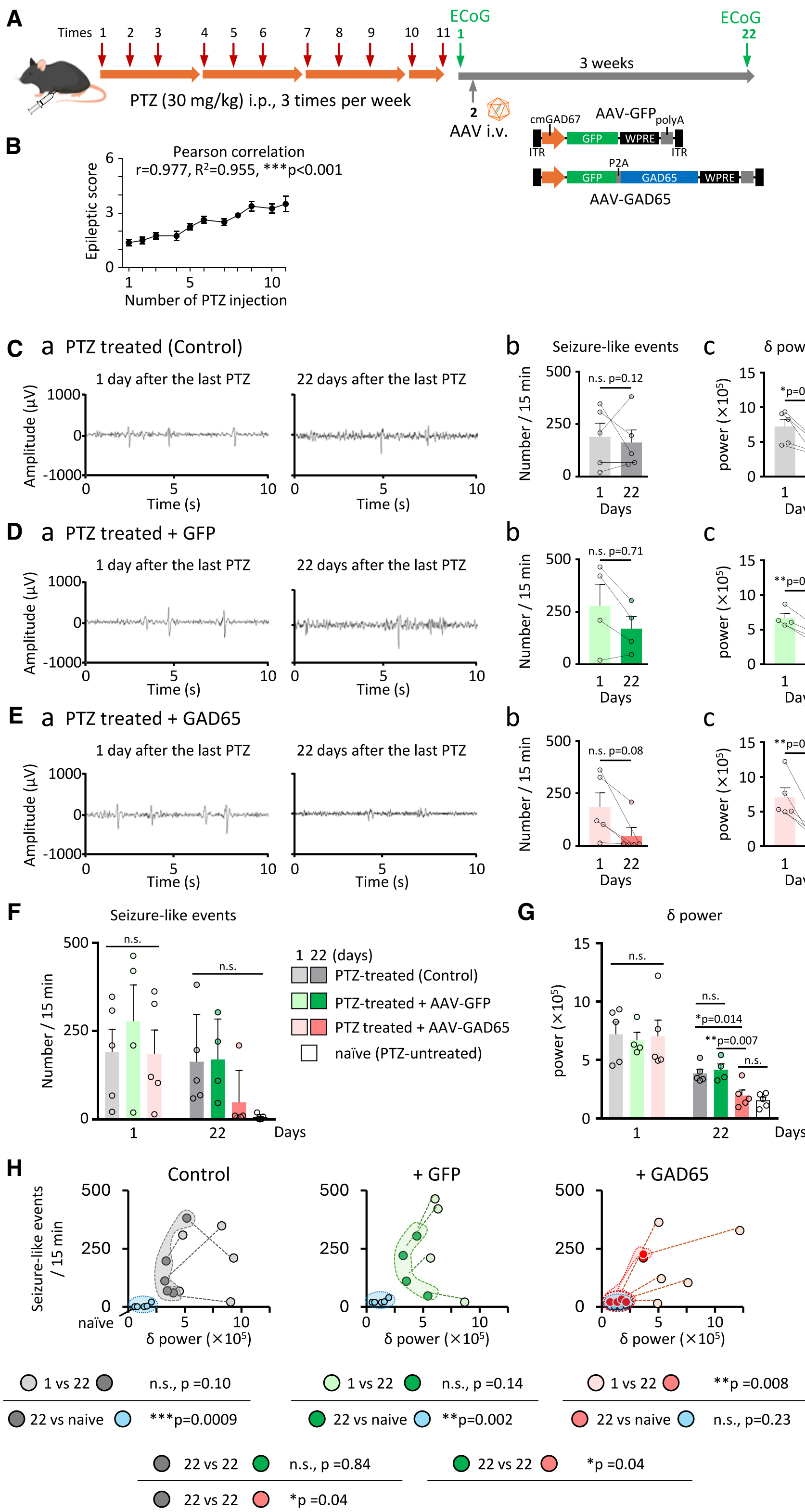

Figure 5. Electrophysiological assessment of PTZ-induced epilepsy and effects of AAV-cmGAD67-GAD65 (hereafter AAV-GAD65)

(A) Experimental timeline of PTZ kindling. Mice received intraperitoneal injections of PTZ (30 mg/kg) three times per week for a total of 11 injections. Electrocorticogram (ECoG) recordings were obtained 1 and 22 days after the final PTZ injection. Mice received an intravenous injection of AAV expressing GFP alone or GFP + GAD65 by the cmGAD67 promoter on the day following the first ECoG recording. The schematic illustrates the AAV-GFP and AAV-GAD65 constructs. (B) Racine scale scores progressively increased with repeated PTZ injections (Pearson correlation: r = 0.977, R^2 = 0.955, ***p < 0.001; n = 8 mice). (C) Representative ECoG traces (a), number of seizure-like events during 15-min epochs (b; n = 5 mice; n.s., p = 0.12), and delta power (c; n = 5 mice; *p = 0.02) from PTZ-treated control mice that did not receive AAV injection. (D and E) Representative ECoG traces (a), number of seizure-like events (b; AAV-GFP, n = 4, n.s., p = 0.71; AAV-GAD65, n = 5, n.s., p = 0.08), and delta power (c; **p = 0.008 for AAV-GFP, **p = 0.007 for AAV-GAD65). Statistical comparisons were made between values obtained 1 and 22 days after the final PTZ injection within each group. (F) Group comparison of seizure-like events at 1 and 22 days; no significant differences among PTZ-treated control, AAV-GFP, and AAV-GAD65 groups. (G) Delta power at 1 and 22 days. At 22 days, AAV-GAD65 significantly reduced delta power compared with PTZ-treated control (*p = 0.01) and AAV-GFP (**p = 0.007), reaching levels comparable to naive mice (AAV-GAD65 vs. naive, n.s., p = 0.46). (H) Scatterplots of seizure-like events versus delta power. Energy distance tests indicated a significant shift between day 1 and day 22 in the AAV-GAD65 group (**p = 0.008; n = 5 mice), with no difference compared to naive controls (p = 0.23). Data represent mean ± SEM. Statistical significance was determined using energy distance test for equality of distributions by R.

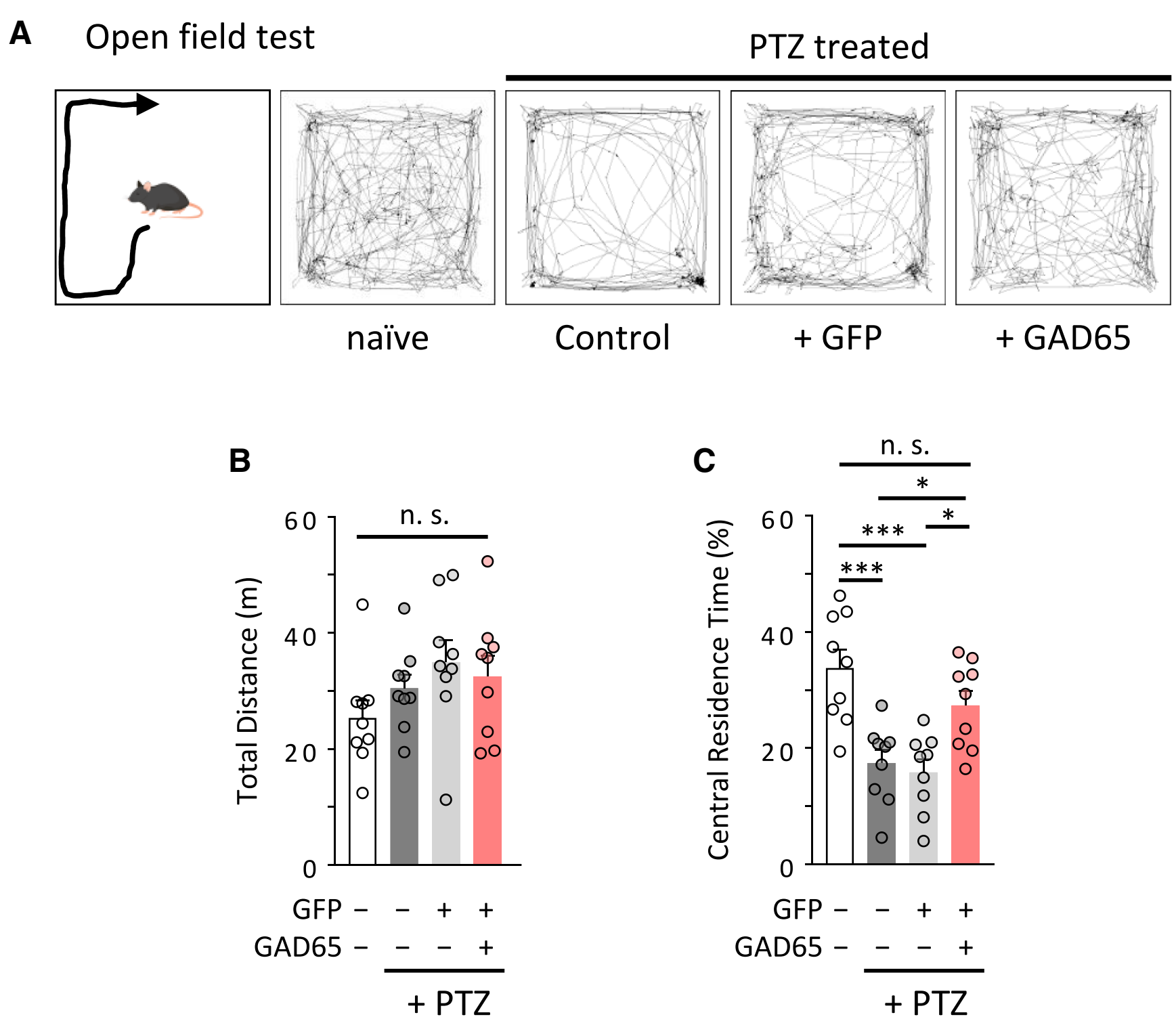

Figure 6. AAV-GAD65 normalizes anxiety-like behavior in PTZ-kindled mice

(A) Schematic of the open-field test (left) and representative movement trajectories of mice: naive, PTZ-treated control, PTZ-treated + AAV-GFP, and PTZ-treated + AAV-GAD65. (B) Total distance traveled did not differ significantly among groups (n = 9 mice per group; n.s.). (C) Percentage of time spent in the central zone was significantly reduced in PTZ-treated control mice compared with naive mice (***p < 0.001) and was restored by AAV-GAD65 treatment compared with PTZ-treated control mice (*p < 0.05). No significant difference was observed between naive and AAV-GAD65 groups. Data represent mean ± SEM (n = 9 mice per group). Statistical significance was determined using one-way ANOVA Tukey's *post hoc* test.

locomotor activity, assessed by total distance traveled, did not differ significantly among groups, indicating that AAV treatment did not alter general motor function (Figures 6A and 6B). Consistently, home-cage monitoring and rotarod/beam-walking testing confirmed that intravenous administration of AAV-GAD65 did not affect circadian activity or motor coordination (Figures S8 and S9). By contrast, the percentage of time spent in the central zone was markedly reduced in PTZ-treated control mice compared with naive mice, reflecting increased anxiety-like behavior (Figures 6A and 6C). Importantly, this behavioral deficit was significantly reversed in mice treated with AAV-GAD65, whereas no improvement was observed in the AAV-GFP group. Notably, the central zone occupancy of AAV-GAD65-treated mice was indistinguishable from that of naive controls (Figure 6C).

These findings are particularly relevant in the clinical context, as anxiety is a common and debilitating comorbidity in patients with epilepsy.[23,24] Thus, AAV-GAD65 not only suppresses epileptiform activity but also normalizes anxiety-like behavior while maintaining normal locomotor, circadian, and motor functions, underscoring its therapeutic potential beyond seizure control.

AAV-GAD65 attenuates seizure progression and improves survival in a severe PTZ model

To further evaluate the therapeutic efficacy of AAV-GAD65, we employed a higher-dose PTZ kindling paradigm (35 mg/kg). This paradigm induces more severe and persistent seizure activity than the standard 30-mg/kg protocol and is therefore used here as a model of severe and recurrent seizures. In this model, Racine-scale scores increased more rapidly, with significantly greater seizure severity across injection numbers compared with the 30-mg/kg group (Figures 7A and 7B). Survival analysis revealed that all mice in the 30-mg/kg group survived after 11 PTZ injections (n = 8), whereas mortality in the 35-mg/kg group began at the seventh injection, resulting in 60% lethality by the end of the protocol (6/10 mice; n = 10) (Figure 7C).

To test whether AAV-GAD65 could mitigate this severe phenotype, vectors (100 μL, 5.0 × 10^{11} vg/mouse) were intravenously delivered 3 weeks before PTZ administration (Figure 7D). In the 35-mg/kg model, seizure progression was significantly attenuated in mice treated with AAV-GAD65, whereas AAV-GFP had no effect (Figure 7E). Moreover, survival curves demonstrated that AAV-GAD65 conferred a marked survival benefit compared with PTZ-treated controls and AAV-GFP groups (Figure 7F). These results indicate that systemic administration of AAV-GAD65 not only reduces seizure severity but also improves survival in a stringent PTZ model, suggesting potential utility for seizure control in experimental models.

AAV-GAD65 suppresses focal hyperexcitability in a chemogenetic seizure model

To further evaluate the effect of AAV-GAD65 in a focal and behaviorally defined seizure model, we employed a chemogenetic approach using Gq-DREADD. An AAV9 vector expressing Gq-DREADD under the excitatory neuron-specific CaMKII promoter (1 μL, 1.0 × 10^{11} vg/mouse) was injected unilaterally into the M1/M2 motor cortex. Three weeks after injection, administration of DCZ (1.0 mg/kg, intraperitoneally [i.p.]) induced moderate seizures (Racine score 3.6 ± 0.2, n = 5), confirming successful establishment of a focal hyperexcitability model (Figures 8A and 8B).

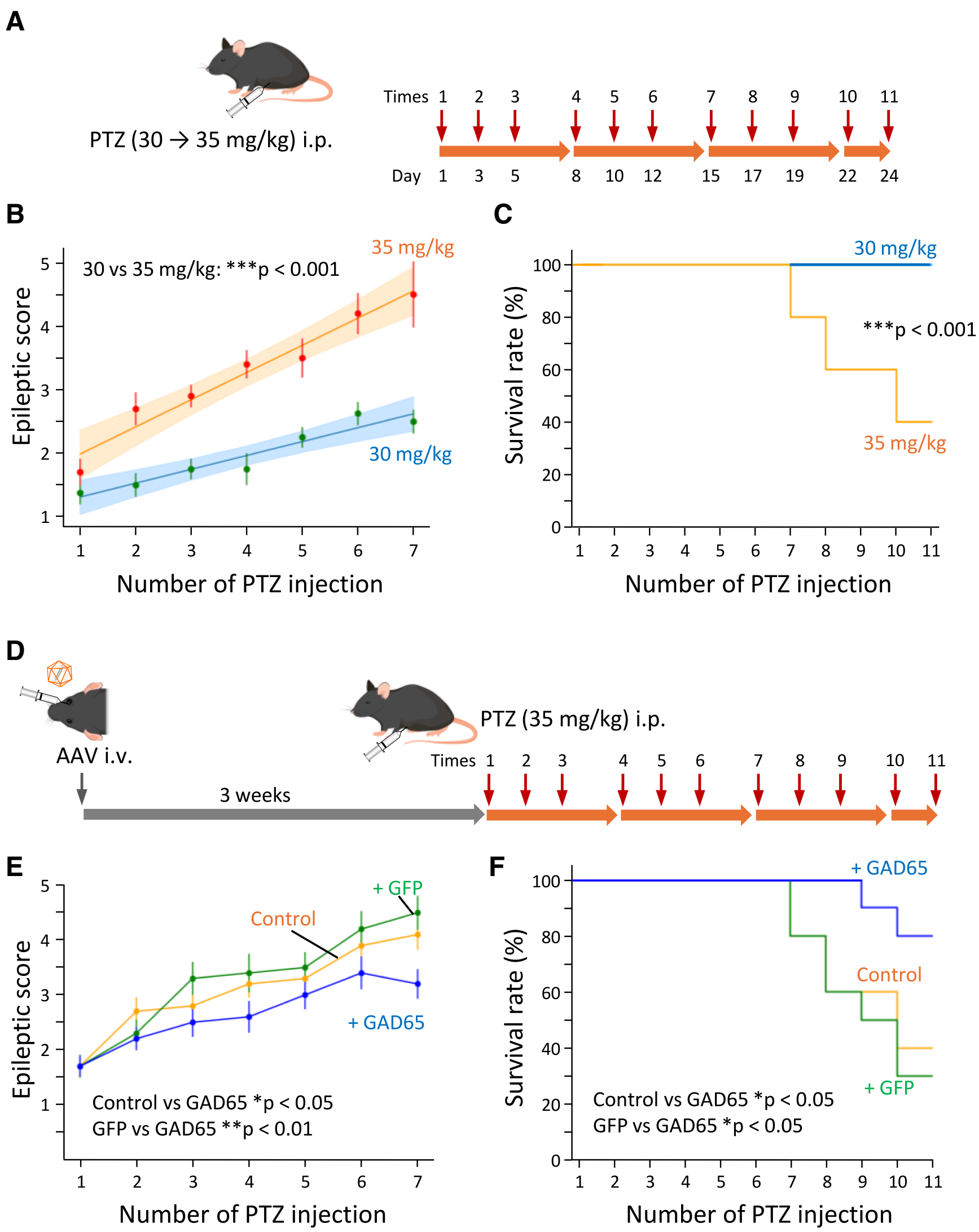

Figure 7. AAV-GAD65 improves seizure progression and survival in a severe PTZ model

(A) Experimental timeline for the PTZ (30 and 35 mg/kg) paradigm. (B) Racine scale scores increased in an injection number–dependent manner, with significantly higher scores in the 35-mg/kg group compared with the 30-mg/kg group (group effect, ***$p < 0.001$). Shaded areas represent 95% confidence intervals (CIs) of the model-derived predicted means, obtained from ordinary least squares (OLS) regression. (C) Comparison of survival curves between mice treated with PTZ at 30 and 35 mg/kg. All eight mice survived after 11 injections at 30 mg/kg, whereas mortality began at the seventh injection in the 35-mg/kg group, and 60% of mice (6 of 10) died (***$p = 0.0007$, Gehan-Breslow-Wilcoxon test). (D) Schematic representation of the experimental procedure. Three weeks before the initiation of PTZ administration, AAV-PHP.eB vectors expressing either GAD65 together with GFP (AAV-GAD65) or GFP alone (AAV-GFP) were delivered intravenously. (E) In PTZ (35 mg/kg)-treated mice, seizure progression was attenuated by AAV-GAD65 (+GAD65) (*$p < 0.05$), whereas AAV-GFP (+GFP) had no effect ($p = 0.53$). (F) Survival curves showed improved survival in the AAV-GAD65 group compared with both PTZ-treated controls and the AAV-GFP group (*$p < 0.05$, Gehan-Breslow-Wilcoxon test; $n = 10$ mice per group).

Mice were divided into DREADD-only, +GFP, and +GAD65 groups, and AAVs (1 µL, 1.0×10^{11} vg/mouse) were injected into the same cortical region. Three weeks later, DCZ was administered again to all groups. In both the DREADD-only and +GFP groups, moderate seizures comparable to those observed after the first DCZ administration were induced (Racine score: DREADD only, 3.2 ± 0.5, $n = 5$; -+GFP, 3.2 ± 0.4, $n = 5$), indicating no significant suppression of seizure severity (Figure 8B; see Videos S3 and S4). In contrast, mice in the +GAD65 group exhibited a marked reduction in seizure severity: three of five mice showed no observable seizures (Racine score = 0), while the remaining two displayed only mild seizures (Racine score = 2), resulting in a low mean seizure score (0.8 ± 0.5, $n = 5$) (Video S5). This was not significantly different from that of naive mice receiving DCZ alone (0.0 ± 0.0, $n = 4$; $p = 0.63$).

Consistent with these behavioral findings, electrophysiological analysis revealed that both seizure-like events (spike amplitude, 300–1,000 µV) and delta-band power were significantly reduced in the +GAD65 group compared with the DREADD-only group. In contrast, no such reductions were observed in the +GFP group (Figures 8C–8H). *Post hoc* histological analysis further demonstrated that DREADD (mCherry) and GAD65 (GFP) were expressed in distinct cell populations within the same motor cortex region, corresponding to excitatory and inhibitory neurons, respectively (Figure 8I). Taken together, these results demonstrate that AAV-GAD65 effectively suppresses focal, behaviorally defined hyperexcitability. This effect is likely mediated by enhanced GABA synthesis in inhibitory neurons, leading to restoration of E/I balance.

DISCUSSION

Advantages of the cmGAD67 promoter for inhibitory neuron-targeted gene therapy

The development of compact, cell-type-specific promoters is critical for maximizing the therapeutic potential of AAV vectors with limited packaging capacity. The cmGAD67 promoter, identified from the *Gad1* locus, is only 410 bp yet drives robust and selective expression in inhibitory neurons. Its compact size preserves space for therapeutic payloads or additional regulatory modules, while its activity overcomes the limitations of previously reported promoters, including mGAD65(delE1)[9] and the Dlx enhancer,[25] the latter of which exhibits comparable promoter strength to

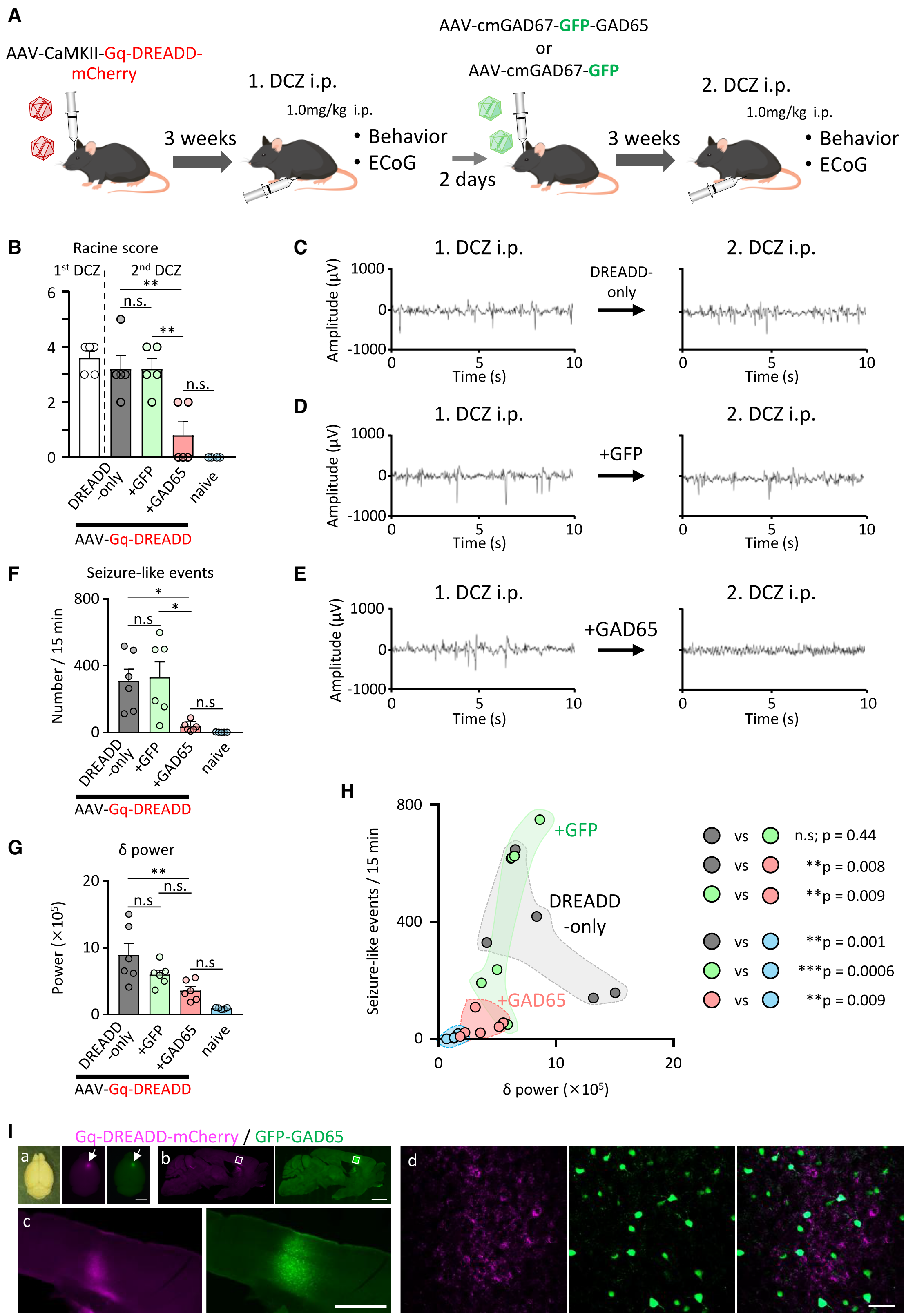

(legend on next page)

mGAD65(delE1) in the forebrain.[9] These features establish cmGAD67 as a practical platform for both experimental applications and translational gene therapy.

Preferential targeting of PV+ interneurons and implications for network control

A key property of the cmGAD67 promoter is its preferential targeting of PV+ interneurons, which provides strong perisomatic inhibition and play a central role in controlling cortical excitability.[17,18] Given their fast-spiking properties and their ability to synchronize and constrain pyramidal neuron output, PV+ interneurons are particularly effective in regulating pathological network activity. By biasing expression toward PV+ interneurons, the cmGAD67 promoter provides a unique advantage over enhancer-based strategies such as Dlx enhancer, which broadly target inhibitory neurons without subtype selectivity.[25]

In contrast to broadly active promoters, inhibitory neuron-enriched expression enables more precise modulation of network excitability while reducing off-target effects. Although low-level expression in excitatory neurons cannot be completely excluded, such expression is unlikely to be detrimental, as GAD65-mediated conversion of glutamate to GABA would be expected to reduce, rather than enhance, excitatory drive.

Notably, AAV-mediated expression of GAD67 under the cytomegalovirus (CMV) promoter has been reported to significantly suppress seizure generation in EL mice, despite non-selective neuronal expression.[26] However, because GAD67 is constitutively active and broadly elevates GABA levels, such non-specific expression, including in excitatory neurons, may perturb physiological neuronal function and network dynamics. In contrast, the cmGAD67 promoter enables inhibitory neuron-enriched expression, allowing more targeted modulation of network excitability while potentially minimizing off-target effects.

Our data further indicate that the inhibitory neuron specificity of the cmGAD67 promoter is largely preserved across different transgenes, as high specificity was maintained following systemic administration of both ChR2 and GAD65 constructs. Moreover, co-administration of AAV-cmGAD67 with an excitatory neuron-specific mCaMKII promoter-driven AAV enabled selective and largely non-overlapping expression of distinct transgenes in inhibitory and excitatory neuronal populations, respectively (Figure S10). However, specificity was reduced following direct parenchymal injection, suggesting that delivery route and local vector concentration influence targeting precision. Notably, even under conditions of reduced specificity, functional outcomes were dominated by inhibitory neuron modulation, indicating that a majority of correctly targeted inhibitory neurons is sufficient to exert network-level effects.

Therapeutic effects of AAV-GAD65 across complementary seizure models

AAV-GAD65 produced robust therapeutic effects across complementary seizure models. In the PTZ-kindled model, AAV-GAD65 reduced seizure-like events, suppressed abnormal oscillatory activity, and improved survival, indicating effective attenuation of global cortical hyperexcitability. In addition, anxiety-like behavior was normalized, a clinically relevant outcome given the high prevalence of psychiatric comorbidities in epilepsy.[23,24]

Importantly, in a chemogenetic focal seizure model, AAV-GAD65 significantly suppressed seizure severity induced by selective activation of excitatory neurons, demonstrating that inhibitory neuron-targeted GAD65 expression can effectively counteract locally driven hyperexcitability. Together, these findings indicate that AAV-GAD65 is effective across both global and circuit-defined contexts of pathological excitation.

Consistent with these functional outcomes, GABA levels were increased in both cortex and hippocampus (Figure S7), linking the therapeutic effects to enhanced inhibitory neurotransmission. Although the 35-mg/kg PTZ paradigm captures key features of severe and recurrent seizures, it does not fully recapitulate pharmacoresistant epilepsy or the chronic and heterogeneous nature of the human condition, and this limitation should be considered when interpreting translational relevance. In addition, seizure responses in chemically induced models such as PTZ are inherently

Figure 8. AAV-GAD65 suppresses seizures in a chemogenetic focal epilepsy model

(A) Schematic of the experimental design. An AAV expressing Gq-DREADD-mCherry under the control of the excitatory neuron-specific CaMKII promoter (1 μL, 1.0×10^9 vg/mouse) was injected into the unilateral motor cortex (M1/M2). Three weeks later, DCZ (1.0 mg/kg, i.p.) was administered, and seizure severity was assessed using the Racine scale and ECoG recordings. After the EcoG recordings, AAV-cmGAD67-GFP-GAD65 or AAV-cmGAD67-GFP (1 μL, 1.0×10^{10} vg/mouse) was injected into the same site. Three weeks after the second injection, DCZ was administered again to evaluate the effects of GAD65 expression on seizure suppression. (B) Racine scores following the first and second DCZ administrations. Comparable seizure severity was observed in the DREADD-only and AAV-GFP (+GFP) groups, whereas significant suppression was observed in the AAV-GAD65 group. The naive group received neither DREADD nor the second AAV injection and was administered DCZ alone. $^{**}p < 0.01$; n.s., not significant. (C–E) Representative ECoG traces following the first (left) and second (right) DCZ administrations. DREADD-only (C), AAV-GFP (D), and AAV-GAD65 (E) groups. (F and G) Quantification of seizure-like events (spikes with amplitudes of 300–1,000 μV) recorded during 15 min after the second DCZ administration (F), and corresponding delta-band power (G). $^{*}p < 0.05$; n.s., not significant. (H) Scatterplots of seizure-like events versus delta power. Energy distance testing showed no significant difference between the DREADD-only and AAV-GFP groups ($p = 0.44$), whereas a significant reduction was observed in the AAV-GAD65 group ($p = 0.009$). The distribution of the AAV-GAD65 group shifted toward that of the naive group ($n = 6$ per group). (I) Representative fluorescence images of brains from AAV-GAD65-treated mice after completion of the experiments. (a) Dorsal view of the whole brain (bright field, mCherry, GFP). Arrows indicate sites of transgene expression. (b) Sagittal sections containing the injection site (mCherry, GFP). (c) Higher-magnification view of the boxed region in (b). (d) Further magnified images (mCherry, GFP, merge). Scale bars, 5 mm (a), 2 mm (b), 1 mm (c), and 50 μm (d).

variable,[27] which may limit statistical power; however, consistent effects across independent measures support the robustness of the observed therapeutic effects. Future studies using optimized paradigms, such as dose-escalation protocols, may further enhance reproducibility.

Translational potential and limitations

The cmGAD67 promoter provides a strong foundation for inhibitory neuron-targeted gene therapy. Its compact size and high specificity make it well suited for AAV-based delivery, supporting the potential of AAV-cmGAD67-GAD65 as a gene-therapy approach applicable to both local and systemic delivery strategies for epilepsy. However, several limitations should be acknowledged. The present study primarily utilized acute seizure models, and validation in chronic epilepsy models and in larger brains will be necessary to establish broader translational relevance. In addition, further evaluation of long-term safety, off-target expression, and peripheral distribution will be required.

Importantly, systemic AAV-GAD65 did not disrupt motor or circadian behaviors (Figures S8 and S9) despite the increase in GABA levels observed in the cortex and hippocampus, suggesting a favorable safety profile. This may reflect an inhibitory neuron-enriched expression pattern, which enhances inhibitory capacity within existing circuits without broadly suppressing physiological brain activity. Consistent with this interpretation, the efficacy observed in the chemogenetic model further indicates that this approach can modulate defined pathological circuits while preserving normal network function. Furthermore, because GAD65 preferentially supports activity-dependent GABA synthesis, its effects may be more pronounced under hyperexcitable conditions than during baseline activity, providing a form of state-dependent suppression of pathological excitation.

The translational feasibility of GAD-based therapy is supported by a first-in-human clinical trial in Parkinson's disease, in which AAV-mediated GAD65 delivery demonstrated safety and therapeutic potential.[10] Although performed in a different disease context and brain region, this study supports the clinical applicability of GAD65-based strategies. Together, these findings highlight AAV-cmGAD67-GAD65 as a promising gene-therapy approach for epilepsy, including focal and circuit-driven forms of epilepsy.

Broader implications for disorders involving E/I imbalance

Disruption of E/I balance is implicated in a wide range of neurological and psychiatric disorders, including epilepsy, schizophrenia, autism spectrum disorders, and neurodegenerative diseases.[1–3,28–32] By enabling compact and selective targeting of inhibitory neurons, particularly PV^+ interneurons, the cmGAD67 promoter provides a versatile platform for both mechanistic studies and therapeutic development. This approach may therefore have broad applicability beyond epilepsy. These findings collectively demonstrate that cell-type-targeted enhancement of inhibitory neurotransmission represents a rational and effective strategy for restoring E/I balance across diverse pathological contexts.

Conclusion

We developed a compact 410-bp GAD67 promoter (cmGAD67) that enables strong and selective expression in inhibitory neurons. AAV-mediated delivery of cmGAD67-GAD65 effectively suppressed epileptiform activity and improved outcomes across complementary seizure models. These findings establish cmGAD67 as a versatile platform for inhibitory neuron-targeted gene therapy with strong translational potential.

MATERIALS AND METHODS

Animals

All animal experiments were approved by the Animal Care and Use Committee of Gunma University (approval nos. 24-053, 24-057, and 25-049) and conducted in accordance with the Japanese Act on the Welfare and Management of Animals and the Guidelines for Proper Conduct of Animal Experiments issued by the Science Council of Japan. Wild-type C57BL/6J mice and VGAT-tdTomato mice (Riken BRC no. RBRC10799) were used for experiments. Mice of both sexes, aged 6–10 weeks, were housed under a 12-h light/dark cycle with *ad libitum* access to food and water. Care was taken to avoid sex-related bias, and all efforts were made to minimize animal suffering and reduce the number of animals used.

AAV vector construction and production

Recombinant AAV vectors were generated by cloning transgene cassettes into AAV2 inverted terminal repeat (ITR)-containing plasmid backbones. Inhibitory neuron-specific promoters used in this study included the previously reported mGAD65 promoter (2,542 bp), the compact mGAD65 (cmGAD65, 486 bp), and the compact mGAD67 (cmGAD67, 410 bp), each driving the expression of reporter (GFP, tdTomato) or effector genes (ChR2(H134R)-EGFP,[33] hM4D(Gi) (Addgene plasmid #50461), or mGAD65). For cell-type-selective dual expression, a 0.4-kb mCaMKII promoter[34] was used for excitatory neuron targeting.

AAV vectors were produced in HEK293T cells by triple transfection of the expression plasmid (pAAV), adenoviral helper plasmid (pHelper, Agilent Technologies), and a Rep/Cap plasmid using polyethylenimine Max (Polysciences) as described previously.[35] Briefly, cells were cultured in DMEM supplemented with 8% fetal bovine serum. Six days after transfection, the culture medium was harvested, and viral particles were precipitated using 8% polyethylene glycol 8000 and 500 mM NaCl, followed by resuspension in D-phosphate-buffered saline (PBS). Viral preparations were purified by iodixanol (Optiprep; AXS-1114542-250ML, Alere Technologies, Oslo, Norway) density-gradient ultracentrifugation and concentrated using Vivaspin Turbo 15 MWCO 100000 PES (VS15T42; Sartorius, Göttingen, Germany). Final titers were measured by qPCR using Power SYBR Green PCR Master Mix (Thermo Fisher Scientific) with primers targeting the WPRE sequence: forward,

5′-CTGTTGGGCACTGACAATTC-3′; reverse, 5′-GAAGGGACGTAGCAGAAGGA-3′.

Intravenous and stereotactic AAV injection

Intravenous injection

Mice were anesthetized with intraperitoneal ketamine (100-mg/kg body weight [BW]) and xylazine (10 mg/kg). Anesthetic depth was verified via the toe-pinch reflex. A total of 100 μL of AAV solution was slowly injected into the retro-orbital venous sinus using a 30-gauge needle (Nipro, Osaka, Japan).

Stereotactic injection

Mice were anesthetized with intraperitoneal ketamine (70-mg/kg BW) and xylazine (7.0-mg/kg BW) and maintained under 0.7% isoflurane anesthesia using an anesthetic vaporizer (MK-AT210; Muromachi Kikai, Fukuoka, Japan). Anesthetic depth was monitored via the toe-pinch reflex. A hole was created over the injection site using a 30G needle. AAV vectors were bilaterally injected into the hippocampus or cerebral cortex. A 10-μL Hamilton syringe with a 33G needle was used with a stereotaxic micromanipulator (SMM-100; Narishige, Tokyo, Japan) mounted on a stereotactic frame (SRS-5-HT; Narishige). The stereotaxic coordinates relative to bregma were as follows: for the hippocampus, anteroposterior (AP), –2.0 mm; mediolateral (ML), ±0.75 mm; dorsoventral (DV), +1.7 mm (advanced to +1.9 mm and then retracted by 0.2 mm); and for the cerebral cortex, AP, +1.5 mm; ML, +1.0 mm; DV, +0.8 mm (advanced to +1.0 mm and then retracted by 0.2 mm).

Brain-tissue preparation and imaging

At 3 weeks post injection, mice used for histological analyses were transcardially perfused with PBS followed by 4% paraformaldehyde (PFA). Brains were post-fixed for 6 h; however, for the experiments shown in Figures 4E–4G and 8, brains were post-fixed overnight. The brains were then coronally sectioned at a thickness of 50 μm using a vibratome (VT1200S; Leica, Wetzlar, Germany). For electrophysiology, acute slices (250–300 μm) were prepared. Fluorescence images were acquired using a fluorescence microscope (VB-7010 or BZ-X800; Keyence, Osaka, Japan) or a confocal microscope (LSM 800; Zeiss, Oberkochen, Germany). Image analysis was performed using ZEISS ZEN and/or Fiji (https://fiji.sc/).

Quantification of GFP fluorescence and cell-type specificity

Whole-brain GFP fluorescence was quantified using Fiji software. Fluorescence intensity was quantified by measuring the brightness of regions of interest (ROIs) in cerebral cortex excluding the olfactory bulb, superior colliculus, and cerebellum. To assess cell-type specificity, GFP^{+} cells co-labeled with tdTomato (VGAT-tdTomato) or with marker antibodies (e.g., PV, SST) were manually counted using a BZ-X800 widefield microscope (Keyence). Co-localization rates were calculated for each section and then averaged per animal. Cell counting was performed in the M1–M2 region of the cerebral cortex.

Immunohistochemistry

Free-floating brain sections were incubated for 30 min at room temperature (RT) in a blocking solution containing 5% normal donkey serum, 0.5% Triton X-100, and 0.05% sodium azide in PBS. Primary antibodies in blocking solution were applied and incubated overnight at 4°C with gentle shaking.

For Figure 2, the following primary antibodies were used:

- Mouse anti-GFP (1:1,000; GTX21218, GeneTex, CA, USA).
- Goat anti-PV (1:200; ab11427, Abcam, Cambridge, UK).
- Rat anti-SST (1:100; MAB354, Millipore, MA, USA).

After two washes with PBS with 0.5% Triton X-100 and three washes with PBS with 0.1% Triton X-100, sections were incubated for 3 h at RT with gentle shaking with fluorophore-conjugated secondary antibodies diluted in the same blocking solution:

- Alexa Fluor 488-conjugated donkey anti-mouse IgG (1:2,000; A32766, Invitrogen, MA, USA).
- Alexa Fluor 555-conjugated donkey anti-goat IgG (1:2,000; A32816, Invitrogen).
- Alexa Fluor 647-conjugated donkey anti-rat IgG (1:2,000; A448272, Invitrogen).

After two washes with PBS with 0.5% Triton X-100, three washes with PBS with 0.1% Triton X-100, and two washes with PBS, sections were mounted using ProLong Diamond (Thermo Fisher Scientific, MA, USA). Co-localization of GFP with these markers was analyzed by fluorescence microscope BZ-X800 or a confocal microscope LSM 800.

For Figures 4E and 4F, the following primary and secondary antibodies were used:

- Rat anti-GFP (1:1,000; 04404–84, Nacalai Tesque, Kyoto, Japan).
- Rabbit anti-GABA (1:250; ABN131, Millipore).
- Alexa Fluor 488-conjugated donkey anti-rat IgG (1:2,000; A48269, Invitrogen).
- Alexa Fluor 555-conjugated donkey anti-rabbit IgG (1:2,000; A32794, Invitrogen).

RT-qPCR quantification

The amount of GFP mRNA in mice brain at 3 weeks after AAV vector administration was analyzed by RT-qPCR. Cortical (near M1–2) samples were homogenized in TRIzol Reagent (Thermo Fisher Scientific) on ice and cooled in liquid nitrogen. RNA was extracted by RNeasy Lipid Tissue Mini Kit (Qiagen, Hilden, Germany) and cDNA was prepared by ReverTra Ace reverse transcriptase (Toyobo, Osaka, Japan). The amount of GFP mRNA was determined using Power SYBR Green Master Mix (Thermo Fisher Scientific) with real-time PCR TP900 (TaKaRa Bio, Shiga, Japan). The relative expression levels were normalized to GAPDH.

The primers are as follows:

GFP: 5′-GGA CGA GCT GTA CAA GTA AAG-3’ (forward) and 5′-GGG AAG CAA TAG CAT GAT ACA AAG G-3’ (reverse).

GAPDH: 5′-GTG TTC CTA CCC CCA ATG TG-3’ (forward) and 5′-GGT GGA AGA GTG GGA GTT GCT G-3’ (reverse).

Electrophysiology

Patch-clamp recordings were performed in acute cerebral slices of the mouse motor cortex including primary and secondary motor areas, as described previously,[9,15,36] with some modifications. Three to 5 weeks after the intravenous injection of a BBB-permeable AAV vector expressing ChR2(H134R)-EGFP-P2A-NLS-EGFP[33] under the control of cmGAD67 promoter into the VGAT-tdTomato mice, as described elsewhere, coronal slices (250–300 μm in thickness) of the cerebral cortex containing the motor areas were prepared using a vibroslicer (VT1200S; Leica). After a two-step incubation of the slices, electrophysiological recordings were conducted.[36] The extracellular solution (artificial cerebrospinal fluid) contained (in mM) 125 NaCl, 2.5 KCl, 2 $CaCl_2$, 1 $MgCl_2$, 1.25 NaH_2PO_4, 26 $NaHCO_3$, and 10 D-glucose, and was continuously bubbled with 95% O_2 and 5% CO_2 gas mixture. Confocal fluorescence signals of the acute brain slices were acquired using a water-cooled EM-CCD camera (512 × 512 pixels; iXon3 DU-897E-CS0-#BV-500; Andor, Belfast, Northern Ireland), a 40× water immersion objective (LUMPLFLN 40XW; Olympus, Tokyo, Japan) and a high-speed spinning-disk confocal unit (CSU-X1; Yokogawa Electric, Tokyo, Japan) mounted to an upright microscope (BX51WI; Olympus, Tokyo, Japan). A 488-nm blue-light beam from a diode laser module (Stradus 488–50, VORTRAN, USA) and a 561-nm laser (LDSYS-561GH-SP43, Solution Systems, Japan) were used as excitation light for GFP and tdTomato, respectively. Emitted fluorescence was collected through a band-pass filter (500–550 nm for GFP and 580–660 nm for tdTomato). For ChR2 activation, the 488-nm blue light used for GFP excitation was applied to the slice for ∼400 ms via the confocal unit.

Whole-cell recordings were performed at RT from inhibitory neurons (i.e., tdTomato-positive cells) in the motor cortex using patch pipettes (1–4 M) pulled from borosilicate glass (Harvard Apparatus) or #0010 glass (PG10165-4, World Precision Instruments). ChR2-mediated inward photocurrents were recorded in voltage-clamp mode at a holding potential of −60 mV. Passive membrane properties of the recorded cells were assessed by applying voltage pulses from −60 to −65 mV for 500 ms. To record light-induced firing of action potentials, current-clamp recordings were performed, and the resting potential was adjusted to −60 mV by current injection. In some cases, cell-attached extracellular recordings (or loose patch recordings) were conducted prior to establishing whole-cell recordings (i.e., before rupturing the patch membrane) to examine ChR2-mediated light-induced spike discharges. All the electrical signals were low-pass filtered at 10 kHz and sampled at 50 kHz with Digidata 1440A (Molecular Devices) and the pCLAMP10 software. The liquid junction potentials were not corrected. Data analysis and statistical tests were performed using Igor Pro9 (Wavemetrics) with Neuromatic (http://www.neuromatic.thinkrandom.com)[37] and GraphPad Prism10 software.

PTZ injection and seizure induction

Seizures were induced with pentylenetetrazol (PTZ; Cayman Chemical, Michigan, USA) based on previous reports.[38,39] PTZ was freshly prepared on the day of each experiment and administered intraperitoneally to 7-week wild-type mice (30 or 35 mg/kg) 3×/week for 11 sessions.

Epilepsy score measurement

Following each PTZ injection, seizure behavior was monitored for 20 min. Seizure severity was assessed using a 7-point epilepsy scoring scale (Racine scale: 0–6), based on established criteria.[38,39] Survival was monitored daily.

ECoG signal analysis

For DREADD experiments in Figures 4E–4G, continuous ECoG was recorded for 30 min immediately before and 30 min after DCZ administration, whereas for the experiments shown in Figure 8, continuous ECoG was recorded only for 30 min after DCZ administration. Recordings were performed 3 weeks after AAV vector injection, with intraperitoneal administration of DCZ (1.0 mg/kg; Cosmo Bio, Tokyo, Japan) as previously described.[40] For PTZ experiments in Figure 5, 30-min recordings were acquired on days 1 and 22 after the final PTZ injection, with AAV administered on day 2 after the final PTZ injection. Delta power (0.5–4 Hz) was used as the primary spectral measure because it showed the most robust and consistent changes associated with epileptiform activity in this experimental setting.

Screw electrodes were placed on the cortical surface with the following coordinates: the recording electrode, AP, −2.00 mm and ML, +2.00 mm from bregma; the reference electrode, AP, −1.50 mm and ML, +1.50 mm from lambda; the ground electrode, AP, −1.50 mm and ML, −1.50 mm from lambda. Electrodes were secured and insulated using GC Unifast III (GC Corporation, Tokyo, Japan). ECoG signals were amplified ×2,000 using a HAS-4 headstage amplifier (Bio Research Center, Nagoya, Japan), then digitized using a PowerLab data-acquisition system (ADInstruments, Dunedin, New Zealand). The signals were recorded at a sampling rate of 1 kHz and high-pass filtered at 0.5 Hz and a low-pass filtered at 60 Hz.

ECoG analyses used the final 15 min of each 30-min recording. Spike counts (number/15 min) were obtained by detecting waveforms with absolute peak amplitude. Spike-like events exceeding 300 μV but less than 1,000 μV were operationally defined as seizure-like events based on amplitude and frequency criteria. Delta power was computed as the mean power within 0.5–4.0 Hz (μV^2). ECoG data were acquired and visualized using LabChart software (ADInstruments), and waveforms were further analyzed with MATLAB R2024b (MathWorks, MA, USA).

Open-field test

The TimeOFCR1E system (O'Hara & Co., Tokyo, Japan) was used. Mice were released in the center of a 40 × 40-cm field under 40 lux illumination, and their behavior was observed for 10 min. Prior to testing, mice were handled for 5 min per day for 1 week to habituate them to the experimenter.

Statistical analysis

Statistical analyses were performed using GraphPad Prism (version 7 or 10) or R (version 4.5.1).

Data are presented as mean ± SEM. For comparisons among multiple groups with a single independent variable, one-way ANOVA was used, followed by Tukey's or Dunnett's *post hoc* test as appropriate. Tukey's *post hoc* test was applied for all pairwise comparisons, whereas Dunnett's test was used when comparing multiple treatment groups against a single control. For comparisons between two independent groups, unpaired two-tailed Student's *t* tests were used. For paired comparisons (e.g., before and after chemogenetic inhibition in the same animals), paired two-tailed Student's *t* tests were applied. For survival analyses, the Gehan-Breslow-Wilcoxon test was applied, which gives greater weight to early events and is appropriate for detecting differences in early mortality.

For comparisons of distributions (e.g., variability or overall distributional differences), the energy-distance test was used. This nonparametric method quantifies differences between groups by comparing pairwise distances among observations, thereby capturing differences in distributional properties including location, dispersion, and shape. The test evaluates differences between distributions as a single global comparison rather than multiple pairwise tests; therefore, no correction for multiple comparisons was applied.

These statistical methods were selected based on the structure of the data, including the number of groups, distributional assumptions, and the nature of the measured variables. All measurements were treated as independent observations across animals unless otherwise specified.

DATA AND CODE AVAILABILITY

- The cmGAD67 promoter described in this study is the subject of patent applications. Plasmid of the cmGAD67 promoter has been deposited to Addgene (#245935) and is available to the research community.
- All data supporting the findings of this study are available within the article and its supplemental files.
- Custom MATLAB scripts used for electrocorticogram (ECoG) spectral analysis are available from the lead contact upon reasonable request.

ACKNOWLEDGMENTS

This work was supported by grants from the Program for Brain Mapping by Integrated Neurotechnologies for Disease Studies (Brain/MINDS; JP20dm0207057/JP21dm0207111 [to H.H.]) and Multidisciplinary Frontier Brain and Neuroscience Discoveries (Brain/MINDS 2.0; JP23wm025001/JP24wm0625103 [to H.H.]) from the Japan Agency for Medical Research and Development (AMED) and by MEXT/JSPS KAKENHI (20K06906/24K10022 [to N.H.], 22K06454/24H01221 [to A.K.], and 23H02791 [to H.H.]), Specific Research Grant by Takeda Science Foundation (2022088468 [to H.H.]) and Next-GIP (JPMJSP2146 [to Y.F.]). The authors thank Dr. Shigeo Miyata for the advice on PTZ-induced epilepsy models; Asako Ohnishi, Nobue McCullough, Chieko Miyazawa, Ayako Sugimoto, and Keiko Sato for AAV vector production; Junko Sugiyama for mouse care; Hiroyoshi Inaba for assistance with ECoG recordings; and Mamiko Suzuki for assistance with open-field testing.

AUTHOR CONTRIBUTIONS

Y.F., A.K., N.H., and H.H. designed the experiments. Y.F. performed most of the experiments. N.H. conducted the slice patch-clamp recordings. K.M. performed direct AAV injections into the mouse brain and assisted with ECoG experiments. R.K. provided the VGAT-tdTomato mice. A.K. supervised the production of various AAV vectors. H.H. supervised and finalized the entire study.

DECLARATION OF INTERESTS

Gunma University, with H.H., A.K., and Y.F. listed as inventors, has filed patent applications in the EU (23803614.9), China (202380040106.1), the US (18/865107), and Japan (2024-520489) for the inhibitory neuron-specific promoter described in this study.

SUPPLEMENTAL INFORMATION

Supplemental information can be found online at https://doi.org/10.1016/j.ymthe.2026.06.007.

REFERENCES

1. Gonzalez-Burgos, G., Cho, R.Y., and Lewis, D.A. (2015). Alterations in cortical network oscillations and parvalbumin neurons in schizophrenia. Biol. Psychiatry *77*, 1031–1040. https://doi.org/10.1016/j.biopsych.2015.03.010.
2. Hattori, R., Kuchibhotla, K.V., Froemke, R.C., and Komiyama, T. (2017). Functions and dysfunctions of neocortical inhibitory neuron subtypes. Nat. Neurosci. *20*, 1199–1208. https://doi.org/10.1038/nn.4619.
3. Marín, O. (2012). Interneuron dysfunction in psychiatric disorders. Nat. Rev. Neurosci. *13*, 107–120. https://doi.org/10.1038/nrn3155.
4. Soghomonian, J.J., and Martin, D.L. (1998). Two isoforms of glutamate decarboxylase: why? Trends Pharmacol. Sci. *19*, 500–505. https://doi.org/10.1016/s0165-6147(98)01270-x.
5. Battaglioli, G., Liu, H., and Martin, D.L. (2003). Kinetic differences between the isoforms of glutamate decarboxylase: implications for the regulation of GABA synthesis. J. Neurochem. *86*, 879–887. https://doi.org/10.1046/j.1471-4159.2003.01910.x.
6. Kanaani, J., Cianciaruso, C., Phelps, E.A., Pasquier, M., Brioudes, E., Billestrup, N., and Baekkeskov, S. (2015). Compartmentalization of GABA synthesis by GAD67 differs between pancreatic beta cells and neurons. PLoS One *10*, e0117130. https://doi.org/10.1371/journal.pone.0117130.
7. Miyata, S., Kakizaki, T., Fujihara, K., Obinata, H., Hirano, T., Nakai, J., Tanaka, M., Itohara, S., Watanabe, M., Tanaka, K.F., et al. (2021). Global knockdown of glutamate decarboxylase 67 elicits emotional abnormality in mice. Mol. Brain *14*, 5. https://doi.org/10.1186/s13041-020-00713-2.
8. Qi, J., Kim, M., Sanchez, R., Ziaee, S.M., Kohtz, J.D., and Koh, S. (2018). Enhanced susceptibility to stress and seizures in GAD65 deficient mice. PLoS One *13*, e0191794. https://doi.org/10.1371/journal.pone.0191794.
9. Hoshino, C., Konno, A., Hosoi, N., Kaneko, R., Mukai, R., Nakai, J., and Hirai, H. (2021). GABAergic neuron-specific whole-brain transduction by AAV-PHP.B incorporated with a new GAD65 promoter. Mol. Brain *14*, 33. https://doi.org/10.1186/s13041-021-00746-1.
10. LeWitt, P.A., Rezai, A.R., Leehey, M.A., Ojemann, S.G., Flaherty, A.W., Eskandar, E.N., Kostyk, S.K., Thomas, K., Sarkar, A., Siddiqui, M.S., et al. (2011). AAV2-GAD gene therapy for advanced Parkinson's disease: a double-blind, sham-surgery controlled, randomised trial. Lancet Neurol. *10*, 309–319. https://doi.org/10.1016/s1474-4422(11)70039-4.
11. Le, T.N., Zhou, Q.P., Cobos, I., Zhang, S., Zagozewski, J., Japoni, S., Vriend, J., Parkinson, T., Du, G., Rubenstein, J.L., and Eisenstat, D.D. (2017). GABAergic Interneuron Differentiation in the Basal Forebrain Is Mediated through Direct Regulation of Glutamic Acid Decarboxylase Isoforms by Dlx Homeobox Transcription Factors. J. Neurosci. *37*, 8816–8829. https://doi.org/10.1523/jneurosci.2125-16.2017.

12. Kaneko, R., Takatsuru, Y., Morita, A., Amano, I., Haijima, A., Imayoshi, I., Tamamaki, N., Koibuchi, N., Watanabe, M., and Yanagawa, Y. (2018). Inhibitory neuron-specific Cre-dependent red fluorescent labeling using VGAT BAC-based transgenic mouse lines with identified transgene integration sites. J. Comp. Neurol. *526*, 373–396. https://doi.org/10.1002/cne.24343.

13. Nitta, K., Matsuzaki, Y., Konno, A., and Hirai, H. (2017). Minimal Purkinje Cell-Specific PCP2/L7 Promoter Virally Available for Rodents and Non-human Primates. Mol. Ther. Methods Clin. Dev. *6*, 159–170. https://doi.org/10.1016/j.omtm.2017.07.006.

14. Aoki, R., Konno, A., Hosoi, N., Kawabata, H., and Hirai, H. (2025). AAV vectors for specific and efficient gene expression in microglia. Cell Rep. Methods *5*, 101116. https://doi.org/10.1016/j.crmeth.2025.101116.

15. Okada, Y., Hosoi, N., Matsuzaki, Y., Fukai, Y., Hiraga, A., Nakai, J., Nitta, K., Shinohara, Y., Konno, A., and Hirai, H. (2022). Development of microglia-targeting adeno-associated viral vectors as tools to study microglial behavior in vivo. Commun. Biol. *5*, 1224. https://doi.org/10.1038/s42003-022-04200-3.

16. Chan, K.Y., Jang, M.J., Yoo, B.B., Greenbaum, A., Ravi, N., Wu, W.L., Sánchez-Guardado, L., Lois, C., Mazmanian, S.K., Deverman, B.E., and Gradinaru, V. (2017). Engineered AAVs for efficient noninvasive gene delivery to the central and peripheral nervous systems. Nat. Neurosci. *20*, 1172–1179. https://doi.org/10.1038/nn.4593.

17. Jézéquel, J., Condomitti, G., Kroon, T., Hamid, F., Sanalidou, S., Garcés, T., Maeso, P., Balia, M., Hu, Z., Sahara, S., and Rico, B. (2025). Cadherins orchestrate specific patterns of perisomatic inhibition onto distinct pyramidal cell populations. Nat. Commun. *16*, 4481. https://doi.org/10.1038/s41467-025-59635-z.

18. Nagy-Pál, P., Veres, J.M., Fekete, Z., Karlócai, M.R., Weisz, F., Barabás, B., Reéb, Z., and Hájos, N. (2023). Structural Organization of Perisomatic Inhibition in the Mouse Medial Prefrontal Cortex. J. Neurosci. *43*, 6972–6987. https://doi.org/10.1523/jneurosci.0432-23.2023.

19. Buzsáki, G., and Draguhn, A. (2004). Neuronal oscillations in cortical networks. Science *304*, 1926–1929. https://doi.org/10.1126/science.1099745.

20. Jiruska, P., de Curtis, M., Jefferys, J.G.R., Schevon, C.A., Schiff, S.J., and Schindler, K. (2013). Synchronization and desynchronization in epilepsy: controversies and hypotheses. J. Physiol. *591*, 787–797. https://doi.org/10.1113/jphysiol.2012.239590.

21. Henning, L., Steinhäuser, C., and Bedner, P. (2021). Initiation of Experimental Temporal Lobe Epilepsy by Early Astrocyte Uncoupling Is Independent of TGFβR1/ALK5 Signaling. Front. Neurol. *12*, 660591. https://doi.org/10.3389/fneur.2021.660591.

22. Khan, D., Bedner, P., Müller, J., Lülsberg, F., Henning, L., Prinz, M., Steinhäuser, C., and Muhammad, S. (2023). TGF-β Activated Kinase 1 (TAK1) Is Activated in Microglia After Experimental Epilepsy and Contributes to Epileptogenesis. Mol. Neurobiol. *60*, 3413–3422. https://doi.org/10.1007/s12035-023-03290-2.

23. Lu, E., Pyatka, N., Burant, C.J., and Sajatovic, M. (2021). Systematic Literature Review of Psychiatric Comorbidities in Adults with Epilepsy. J. Clin. Neurol. *17*, 176–186. https://doi.org/10.3988/jcn.2021.17.2.176.

24. Niu, C., Li, P., Du, X., Zhao, M., Wang, H., Yang, D., Wu, M., and Jing, W. (2024). Risk factors for anxiety in patients with epilepsy: A meta-analysis. Epilepsy Behav. *153*, 109665. https://doi.org/10.1016/j.yebeh.2024.109665.

25. Dimidschstein, J., Chen, Q., Tremblay, R., Rogers, S.L., Saldi, G.A., Guo, L., Xu, Q., Liu, R., Lu, C., Chu, J., et al. (2016). A viral strategy for targeting and manipulating interneurons across vertebrate species. Nat. Neurosci. *19*, 1743–1749. https://doi.org/10.1038/nn.4430.

26. Shimazaki, K., Kobari, T., Oguro, K., Yokota, H., Kasahara, Y., Murashima, Y., Watanabe, E., Kawai, K., and Okada, T. (2019). Hippocampal GAD67 Transduction Using rAAV8 Regulates Epileptogenesis in EL Mice. Mol. Ther. Methods Clin. Dev. *13*, 180–186. https://doi.org/10.1016/j.omtm.2018.12.012.

27. Kandratavicius, L., Balista, P., Lopes-Aguiar, C., Ruggiero, R., Umeoka, E., Garcia-Cairasco, N., Bueno-Junior, L., and Leite, J. (2014). Animal models of epilepsy: use and limitations. Neuropsychiatr. Dis. Treat. *10*, 1693–1705. https://doi.org/10.2147/ndt.S50371.

28. Leitch, B. (2024). Parvalbumin Interneuron Dysfunction in Neurological Disorders: Focus on Epilepsy and Alzheimer's Disease. Int. J. Mol. Sci. *25*, 5549. https://doi.org/10.3390/ijms25105549.

29. Najm, R., Jones, E.A., and Huang, Y. (2019). Apolipoprotein E4, inhibitory network dysfunction, and Alzheimer's disease. Mol. Neurodegener. *14*, 24. https://doi.org/10.1186/s13024-019-0324-6.

30. Perez, C., Ziburkus, J., and Ullah, G. (2016). Analyzing and Modeling the Dysfunction of Inhibitory Neurons in Alzheimer's Disease. PLoS One *11*, e0168800. https://doi.org/10.1371/journal.pone.0168800.

31. Smeralda, C.L., Pandit, S., Turrini, S., Reilly, J., Palmisano, A., Sprugnoli, G., Hampel, H., Benussi, A., Borroni, B., Press, D., et al. (2024). The role of parvalbumin interneuron dysfunction across neurodegenerative dementias. Ageing Res. Rev. *101*, 102509. https://doi.org/10.1016/j.arr.2024.102509.

32. Xu, Y., Zhao, M., Han, Y., and Zhang, H. (2020). GABAergic Inhibitory Interneuron Deficits in Alzheimer's Disease: Implications for Treatment. Front. Neurosci. *14*, 660. https://doi.org/10.3389/fnins.2020.00660.

33. Vormstein-Schneider, D., Lin, J.D., Pelkey, K.A., Chittajallu, R., Guo, B., Arias-Garcia, M.A., Allaway, K., Sakopoulos, S., Schneider, G., Stevenson, O., et al. (2020). Viral manipulation of functionally distinct interneurons in mice, non-human primates and humans. Nat. Neurosci. *23*, 1629–1636. https://doi.org/10.1038/s41593-020-0692-9.

34. Scheyltjens, I., Laramée, M.E., Van den Haute, C., Gijsbers, R., Debyser, Z., Baekelandt, V., Vreysen, S., and Arckens, L. (2015). Evaluation of the expression pattern of rAAV2/1, 2/5, 2/7, 2/8, and 2/9 serotypes with different promoters in the mouse visual cortex. J. Comp. Neurol. *523*, 2019–2042. https://doi.org/10.1002/cne.23819.

35. Konno, A., and Hirai, H. (2020). Efficient whole brain transduction by systemic infusion of minimally purified AAV-PHP.eB. J. Neurosci. Methods *346*, 108914. https://doi.org/10.1016/j.jneumeth.2020.108914.

36. Hosoi, N., Shibasaki, K., Hosono, M., Konno, A., Shinoda, Y., Kiyonari, H., Inoue, K., Muramatsu, S.I., Ishizaki, Y., Hirai, H., et al. (2019). Deletion of Class II ADP-Ribosylation Factors in Mice Causes Tremor by the Nav1.6 Loss in Cerebellar Purkinje Cell Axon Initial Segments. J. Neurosci. *39*, 6339–6353. https://doi.org/10.1523/jneurosci.2002-18.2019.

37. Rothman, J.S., and Silver, R.A. (2018). NeuroMatic: An Integrated Open-Source Software Toolkit for Acquisition, Analysis and Simulation of Electrophysiological Data. Front. Neuroinform. *12*, 14. https://doi.org/10.3389/fninf.2018.00014.

38. Dhir, A. (2012). Pentylenetetrazol (PTZ) kindling model of epilepsy. Curr. Protoc. Neurosci. *Chapter 9*, Unit9.37. https://doi.org/10.1002/0471142301.ns0937s58.

39. Shimada, T., and Yamagata, K. (2018). Pentylenetetrazole-Induced Kindling Mouse Model. J. Vis. Exp. 56573. https://doi.org/10.3791/56573.

40. Nagai, Y., Miyakawa, N., Takuwa, H., Hori, Y., Oyama, K., Ji, B., Takahashi, M., Huang, X.P., Slocum, S.T., DiBerto, J.F., et al. (2020). Deschloroclozapine, a potent and selective chemogenetic actuator enables rapid neuronal and behavioral modulations in mice and monkeys. Nat. Neurosci. *23*, 1157–1167. https://doi.org/10.1038/s41593-020-0661-3.

YMTHE, Volume *34*

Supplemental Information

A compact GAD67 promoter enables inhibitory neuron-targeted AAV gene therapy for seizure suppression

Yuuki Fukai, Ayumu Konno, Nobutake Hosoi, Karin Miyakawa, Ryosuke Kaneko, and Hirokazu Hirai

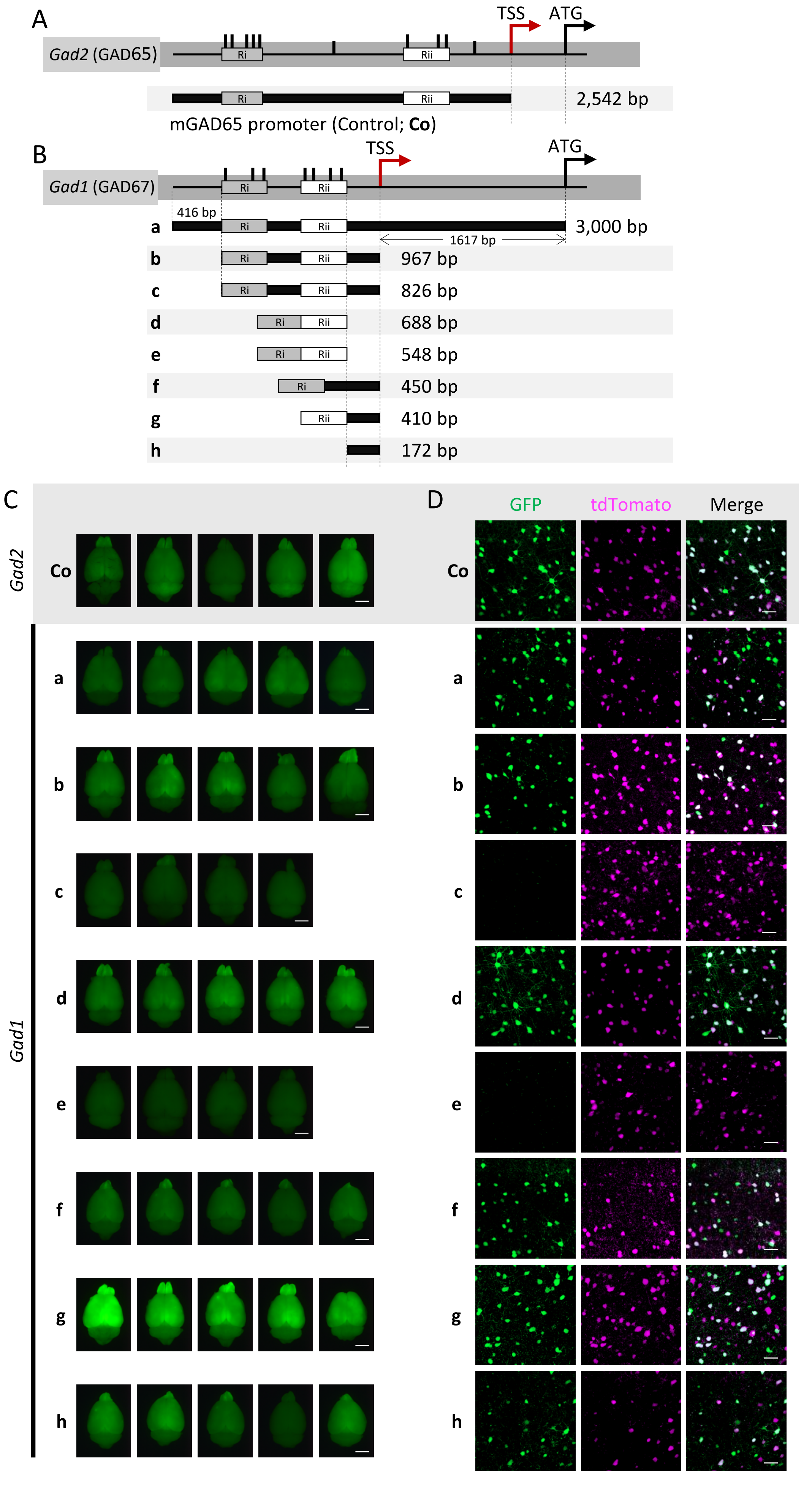
A
TSS
ATG
Gad2 (GAD65)
Ri
Rii
2,542 bp
mGAD65 promoter (Control; Co)
B
TSS
ATG
Gad1 (GAD67)
416 bp
a
3,000 bp
1617 bp
b
967 bp
c
826 bp
d
688 bp
e
548 bp
f
450 bp
g
410 bp
h
172 bp
C
Gad2
Co
Gad1
a
b
c
d
e
f
g
h
D
GFP
tdTomato
Merge
Co
a
b
c
d
e
f
g
h

Figure S1. Strategy for identifying a minimal promoter region specific to GABAergic neurons.

(A) Schematic representation of the *Gad2* locus. Vertical bars indicate binding motifs for the inhibitory neuron–specific transcription factor Dlx. Gray (Region i, Ri) and white (Region ii, Rii) boxes represent regions enriched in Dlx binding motifs. The 2,542 bp region upstream of the transcription start site (TSS) corresponds to the previously reported inhibitory neuron–specific mGAD65(delE1) promoter, which was used as a control (Co). (B) Schematic representation of the *Gad1* locus and promoter candidates. Promoter candidates were initially cloned from ~3 kb upstream of the ATG start codon and subsequently truncated based on the TSS, retaining either or both Ri and Rii regions. (C) Whole-brain GFP fluorescence images from mice intravenously injected with PHP.eB vectors (100 µL, 5.0×10^{11} vg/mouse) expressing GFP under the control of the control mGAD65(delE1) promoter (Co) or mGAD67 promoter candidates (a–h). Scale bars, 5 mm. (D) Representative native fluorescence images of GFP and tdTomato in the motor cortex, along with merged images. Scale bars, 50 µm.

Sequence of cmGAD67 promoter

Rii: -410 to -142
Putative core promoter: -143 to -1

ACAAAATTCTTCGTAGGAATTATATTTTCCCTTGCCCTCACCCAACATC
GCCTATCTCAAAAATAATTTAAAAACAAAAAACAAACAAACAAACAA
AAAACAGAGCGTGCTGAGTGCATTCTGGATTACTCATAGGACTTTGT
CACACACACCCTCCTTTCTGGTCGCAAACCCGTGAGCTGGATTTATA
ATCGCCCTACAAAGCTCCAGAGGCAGTCAGACACCTGCAAAGGAGC
CCCAGCGCTCCGCGGACGAGCTGCCCCCGCGAGCAACGGCCTCGT
GATTCCCCCCGCCGAGCGGGTCCCCGCCTCCCCACTCCGCCCCCGCC
TCCCCCAAGCCCAGCGGCCGCCTCTCCGGATCTCTCCCTTCTTCAGG
CTCTCCCGTGCCGGACCAGGGATCGTGCAAGCAAG

Underlined sequences indicate TAAT/ATTA motifs, which are putative DLX-binding sites.
*+1 = Transcription start site (TSS)

Figure S2. Nucleotide sequence and structural features of the cmGAD67 promoter.
The 410-bp cmGAD67 promoter sequence is shown with key regulatory elements annotated. The transcription start site (TSS) is defined as +1, and nucleotide positions are shown relative to the TSS. The core promoter region (light blue background) and the Rii enhancer region (yellow background) are highlighted. Putative Dlx binding motifs (TAAT/ATTA) within the Rii region are indicated by underlining. This sequence corresponds to the cmGAD67 promoter construct shown in Fig. 1C (g).

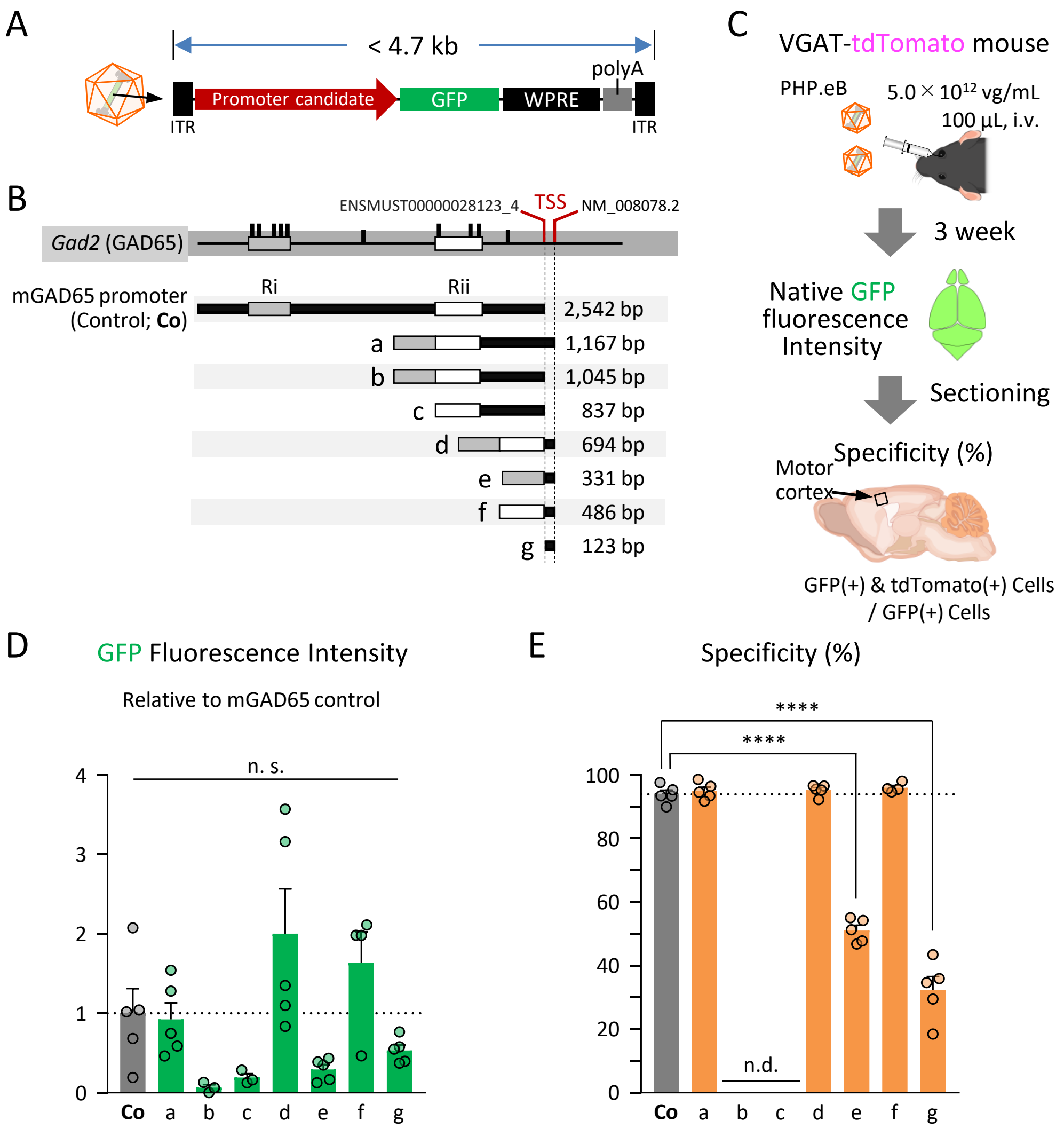

Figure S3. Characterization of the compact GAD65 promoter (cmGAD65).
(A) Schematic diagram of the AAV genome used in this study. When GFP, WPRE, and SV40 polyA are included between the ITRs, the promoter sequence must be shorter than 3 kb to remain within the AAV packaging limit (~4.7 kb). (B) Schematic representation of the *Gad2* locus and promoter candidates. Vertical bars indicate binding motifs for the inhibitory neuron–specific transcription factor Dlx. Gray (Region i, Ri) and white (Region ii, Rii) boxes represent regions enriched in Dlx binding motifs. The 2,542 bp region upstream of the transcription start site (TSS) (from genome database ID: ENSMUST00000028123_4) corresponds to the previously reported inhibitory neuron–specific mGAD65(delE1) promoter, which was used as a control (Co). In the present study, we used the NM_008078.2 TSS as a reference, and promoter candidates were produced based on the TSS, retaining either or both Ri and Rii regions (a – g). (C) Experimental design. AAV-PHP.eB vectors expressing GFP under the control of *Gad2*-derived promoter candidates (100 µL, 5.0 × 10^{11} vg/mouse) were intravenously administered via the retro-orbital sinus into VGAT-tdTomato mice, in which inhibitory neurons express the red fluorescent protein tdTomato. Three weeks after injection, mice were perfused, and GFP fluorescence on the dorsal surface of the whole brain was imaged to assess promoter activity. Sagittal sections were then prepared, and inhibitory neuron specificity of GFP expression was quantified in the M1/M2 motor cortex. (D) Quantification of dorsal whole-brain GFP fluorescence intensity. Fluorescence intensity in the mGAD65(delE1) control group was normalized to 1. (E) Quantification of the proportion of GFP-positive cells that were also tdTomato-positive (indicative of inhibitory neuron specificity). Each dot represents an individual mouse. ****p < 0.0001, n.s., not significant by one-way ANOVA with Dunnett's post hoc test. n.d., not determined.

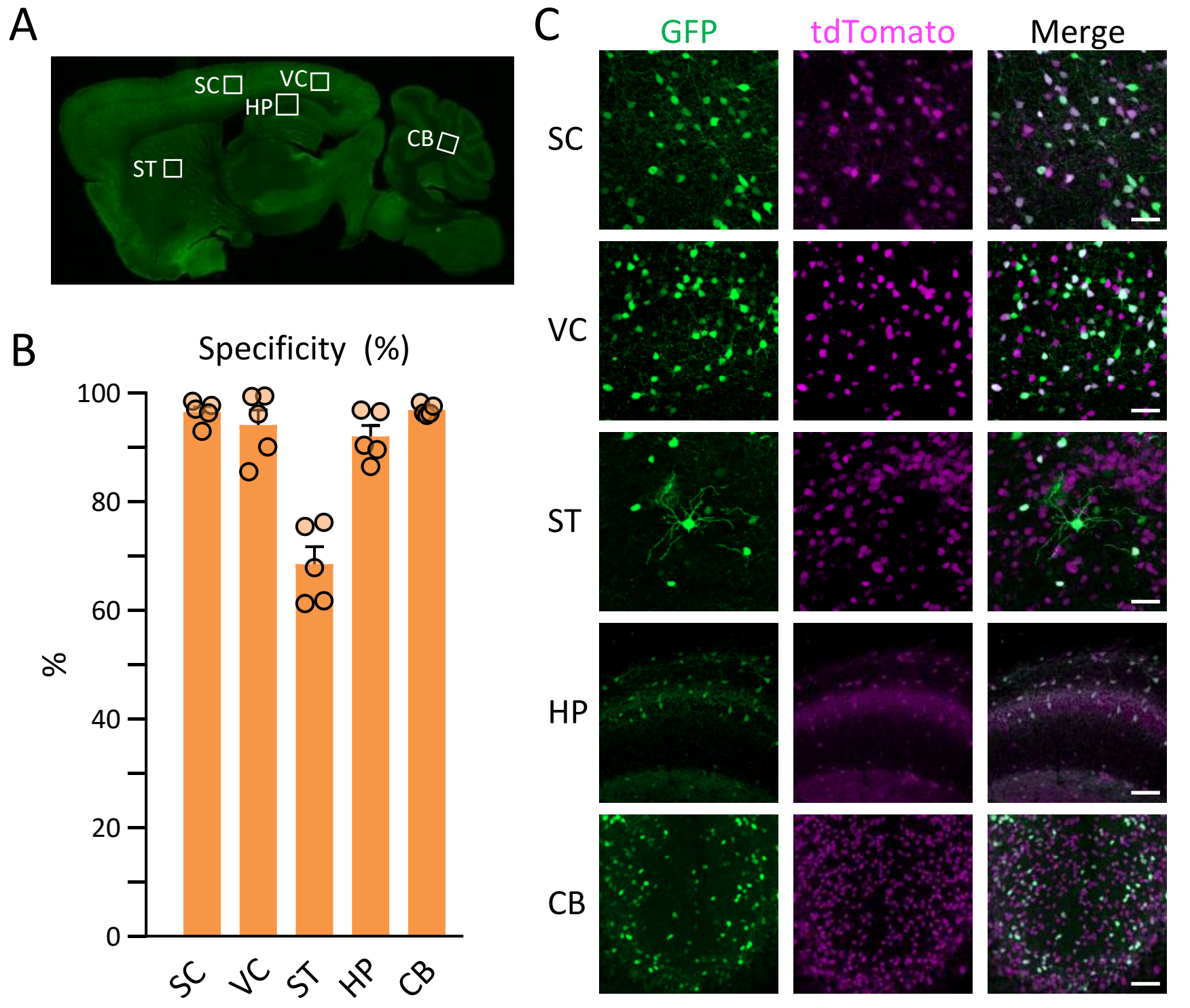

Figure S4. Inhibitory neuron specificity of the cmGAD67 promoter across multiple brain regions in mice

Inhibitory neuron specificity of the cmGAD67 promoter was evaluated across multiple brain regions beyond the motor cortex. VGAT-tdTomato mice were intravenously injected with AAV-PHP.eB-cmGAD67-GFP (100 µL, 5.0×10^{11} vg/mouse). Three weeks after injection, brains were perfusion-fixed, and sagittal sections were prepared for analysis. (A) Representative sagittal brain section showing GFP fluorescence. Major regions are indicated: SC, sensory cortex; VC, visual cortex; ST, striatum; HP, hippocampus; CB, cerebellum. (B) Quantification of inhibitory neuron specificity in each brain region, defined as the percentage of tdTomato-positive cells among GFP-expressing cells within a defined area (542.5 µm × 722.4 µm). Each dot represents one mouse (n = 5), and bars indicate mean ± SEM. High co-localization indicates robust inhibitory neuron specificity across all examined regions. (C) Higher-magnification images of the boxed regions in (A), showing GFP (green), tdTomato (magenta), and merged signals. Scale bars, 50 µm.

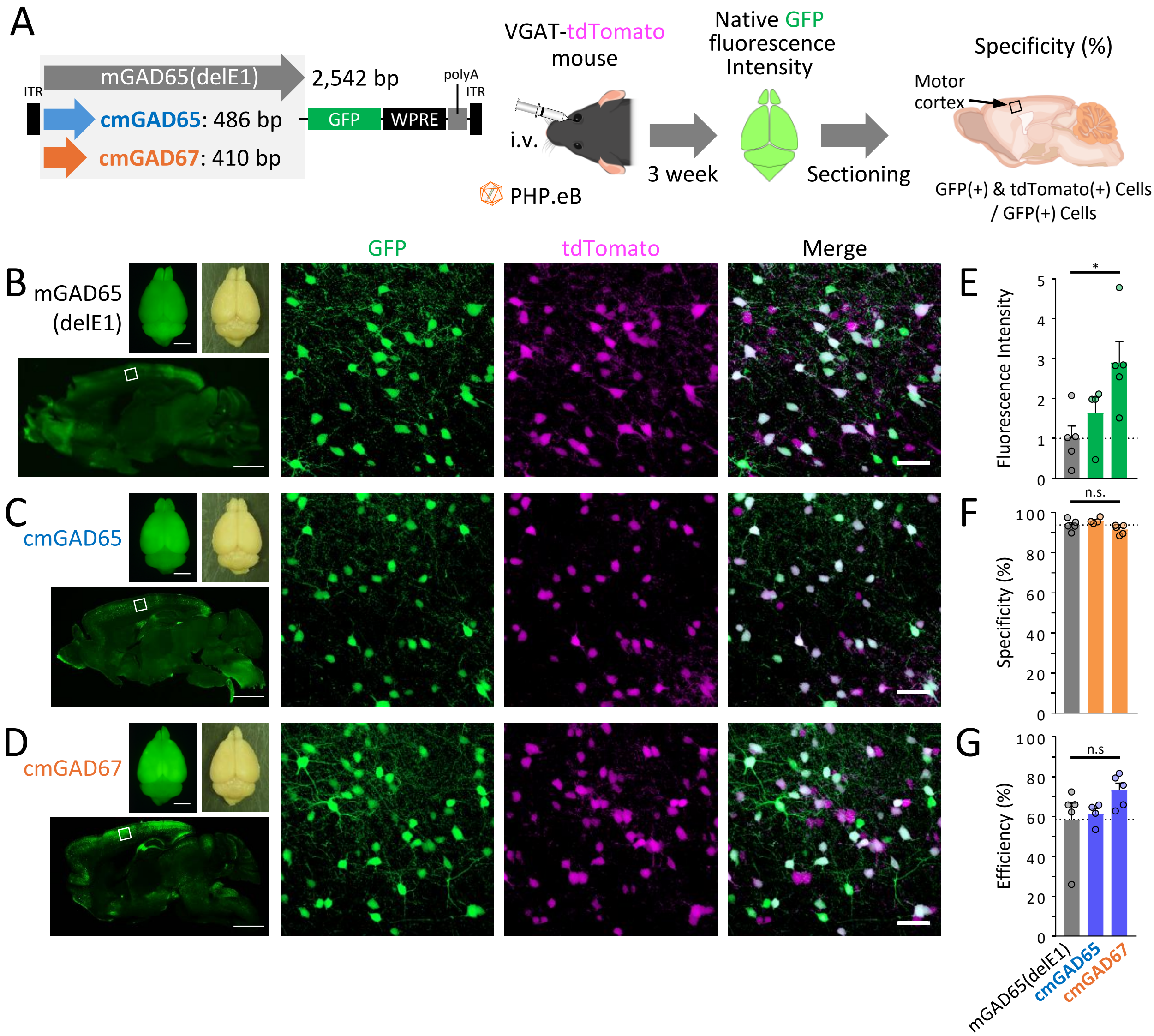

Figure S5. Comparative analysis of expression profile of mGAD(delE1) and cmGAD promoters after intravenous administration of AAV-PHP.be

(A) Schematic representation of the experimental procedure. AAV-PHP.eB expressing GFP under the control of the conventional mGAD65(delE1) promoter, cmGAD65 promoter, or cmGAD67 promoter was intravenously administered to VGAT-tdTomato mice. Three weeks post-injection, whole-brain GFP fluorescence intensity was measured. Sagittal brain sections were then prepared to assess the specificity of GFP expression in inhibitory neurons within the primary motor cortex. (B-D) Representative images of GFP fluorescence and bright-field views of the whole brain (top left paired images) and sagittal section GFP fluorescence images (bottom left) for the mGAD65(delE1) (B), cmGAD65 (C), and cmGAD67 (D) promoters. The three rightmost columns show magnified fluorescence images of the primary motor cortex (corresponding to the boxed region in the bottom left images). Scale bars, 5 mm (whole brains), 2 mm (sagittal sections) and 50 µm (right panels). (E) Quantification of whole-brain GFP fluorescence intensity except for the cerebellar area. Relative intensities are shown with the mGAD65(delE1) promoter set as 1. (F) Graph showing the specificity of GFP expression in inhibitory neurons. (G) Quantification of the proportion (efficiency) of inhibitory neurons in the primary motor cortex expressing GFP. Dots in the graphs represent individual mouse data points. *$p < 0.05$, n.s., not significant by one-way ANOVA with Dunnett's post hoc test.

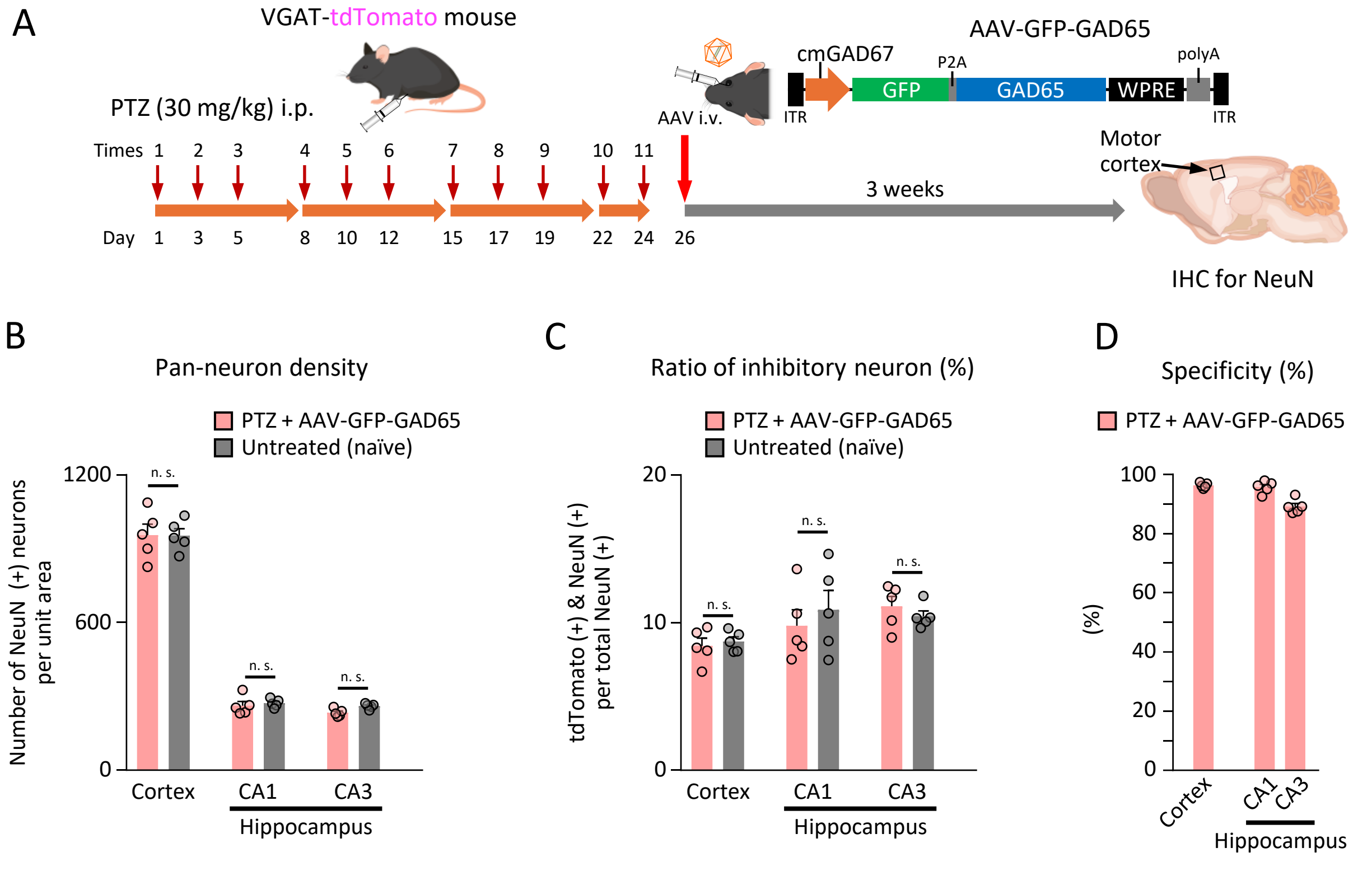

Figure S6. Inhibitory neuron proportion and AAV-cmGAD67-GAD65 expression specificity are preserved after PTZ treatment.

(A) Experimental design. VGAT-tdTomato mice, in which inhibitory neurons are labeled with the red fluorescent protein tdTomato, received 11 intraperitoneal injections of PTZ (30 mg/kg). Two days after the final injection, AAV-PHP.eB-cmGAD67-GFP-P2A-GAD65 was administered intravenously. Three weeks later, brains were perfusion-fixed, and sagittal sections were prepared for immunohistochemical analysis using an anti-NeuN antibody. Analyses were performed in the motor cortex (M1/M2) and hippocampal CA1 and CA3 regions. Untreated VGAT-tdTomato mice (no PTZ or AAV) were used as controls. (B) No significant difference in the number of NeuN-positive neurons was observed between PTZ-treated (pink bars) and untreated (gray bars) groups (n = 5 mice per group). Quantification was performed within images of a defined area (542.5 µm × 722.4 µm). (C) The proportion of tdTomato-positive inhibitory neurons among NeuN-positive neurons did not differ significantly between PTZ-treated and untreated groups (n = 5 mice per group), based on quantification within the same defined area (542.5 µm × 722.4 µm). (D) In mice that received 11 PTZ injections, inhibitory neuron specificity of AAV-cmGAD67-GAD65 expression remained well preserved (n = 5 mice). n.s., not significant by unpaired two-tailed Student's t-test.

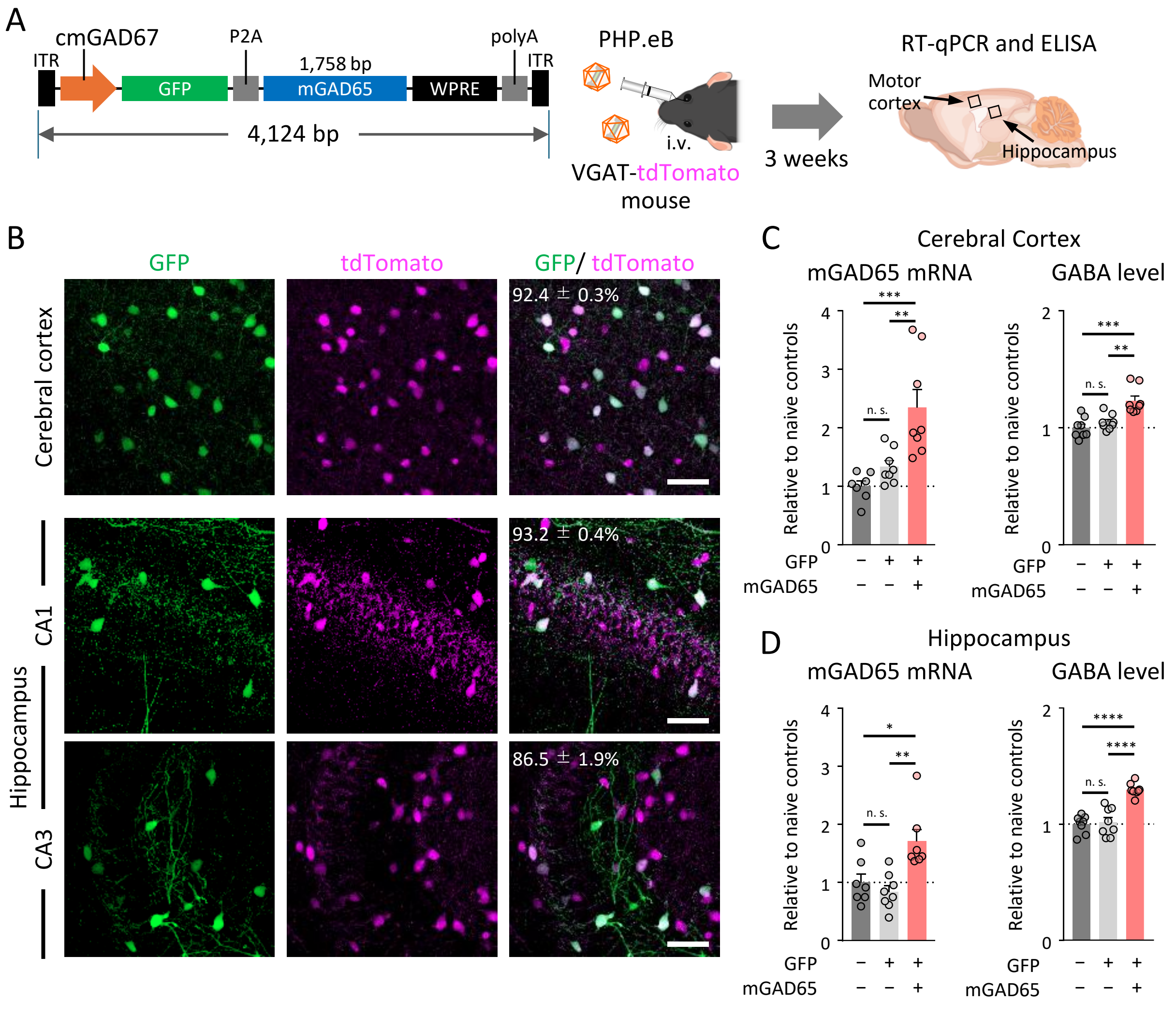

Figure S7. AAV-cmGAD67-GAD65 elevates cortical and hippocampal GABA levels.
(A) Schematic representation of the experimental procedure. AAV-PHP.eB expressing GFP and *mGAD65* under the control of the cmGAD67 promoter was intravenously administered to VGAT-tdTomato mice. Three weeks post-injection, tissue samples from the primary motor cortex and hippocampus were collected for quantification of *mGAD65* mRNA using RT-qPCR and measurement of GABA levels via ELISA. (B) Fluorescence images of native GFP and native tdTomato in the primary motor cortex and hippocampal CA1 and CA3 regions. The numbers in the upper left corner of the rightmost panel indicate the percentage of tdTomato-positive (inhibitory) neurons among GFP (*mGAD65*)-expressing cells (Average ± SEM., n = 5 mice). Scale bars, 50 µm. (C,D) Quantification of *mGAD65* mRNA (left) and GABA levels (right) in the primary motor cortex (C) and hippocampus (D). The values were normalized to those obtained from untreated mice (leftmost bar). Relative expression levels are shown for mice injected with AAV-PHP.eB expressing GFP alone and those expressing both GFP and *mGAD65*. Dots in the graphs represent individual mouse data points. *p < 0.05, **p < 0.01, ***p < 0.001, ****p < 0.0001, n.s., not significant; one-way ANOVA with Tukey's post hoc test.

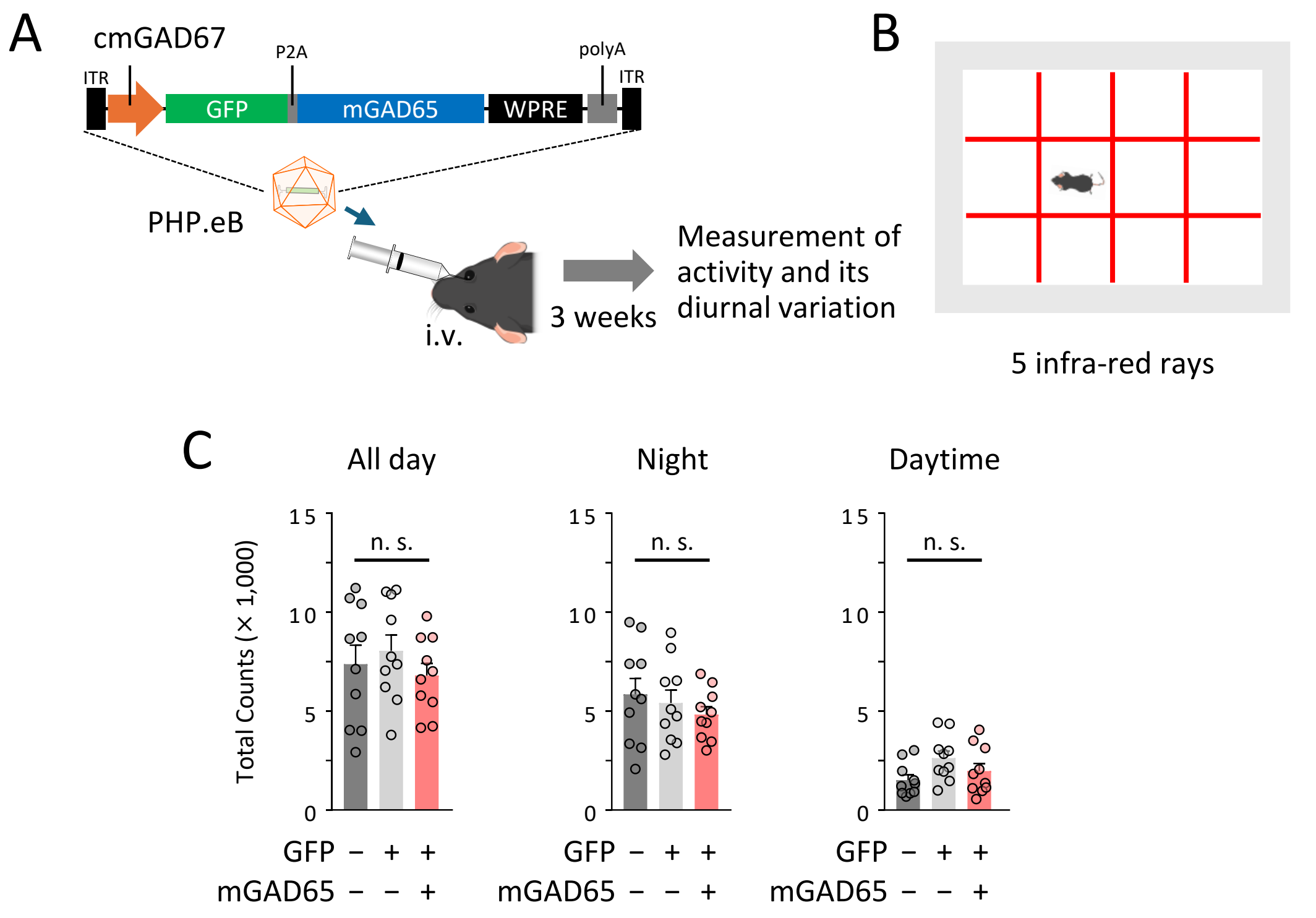

Figure S8. Safety assessment: AAV-cmGAD67-GAD65 does not alter circadian activity.
(A) Schematic representation of the experimental procedure. AAV-PHP.eB expressing GFP and GAD65 under the control of the cmGAD67 promoter was intravenously administered. Three weeks post-injection, locomotor activity and its circadian variations were measured. (B) Illustration of the activity monitoring apparatus. Mice were placed in a cage segmented by five horizontal and five vertical infrared beams, and locomotor activity was quantified by counting the number of infrared beam crossings. (C) Quantification of the number of infrared beam crossings over the entire 24-hour period, during the nocturnal phase, and during the diurnal phase. Dots in the graph represent individual mouse data points. n.s., not significant; one-way ANOVA with Tukey's post hoc test.

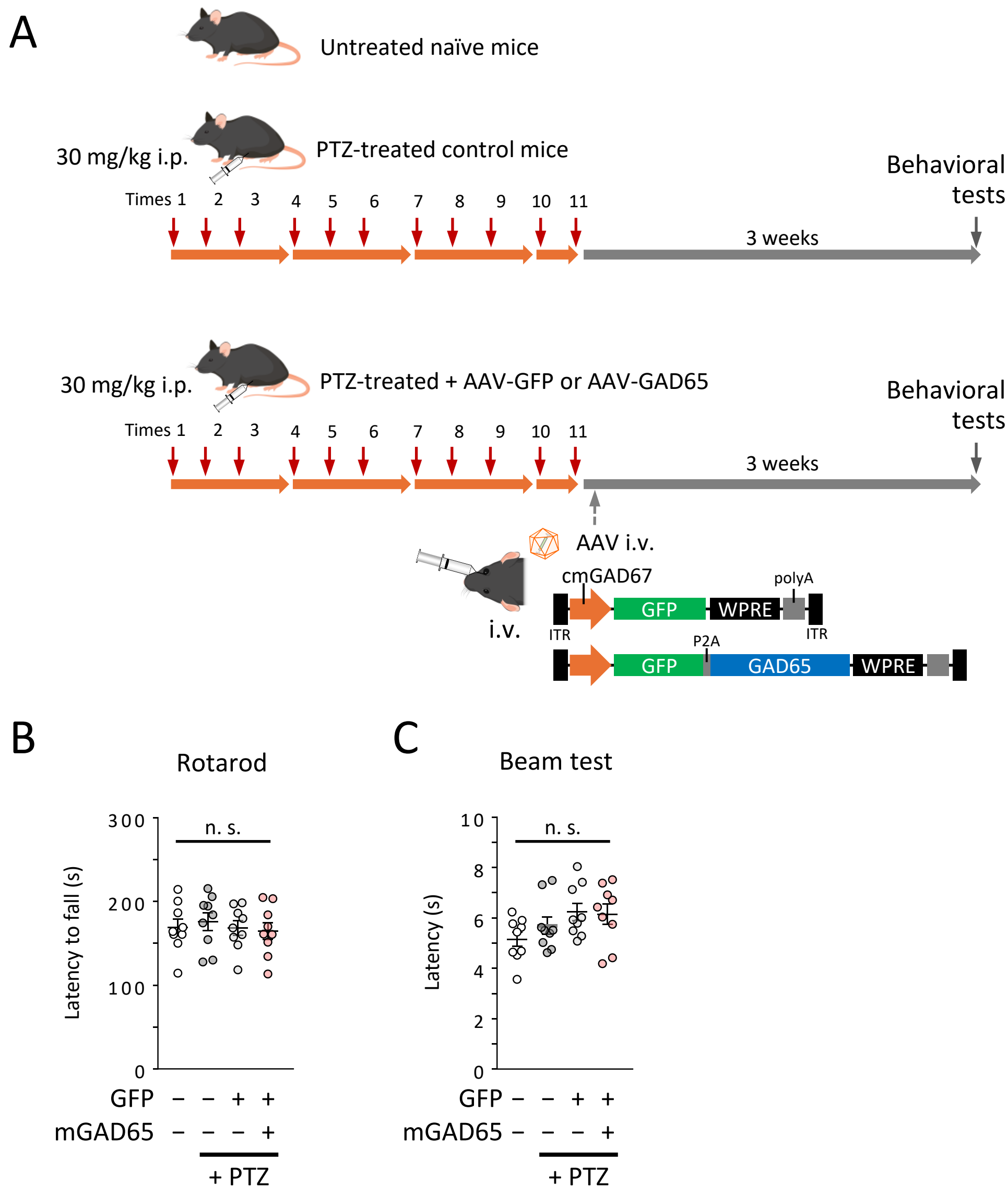

Figure S9. Safety assessment: AAV-cmGAD67-GAD65 does not impair motor coordination.
(A) Schematic representation of the experimental procedure. Naïve mice were used as controls. The rest of three groups received pentylenetetrazole (PTZ, 30 mg/kg) intraperitoneally three times per week (every other day) for a total of 11 sessions. One group were treated only with PTZ. Two other groups received intravenous injection of AAV-PHP.eB expressing GFP-P2A-GAD65 or GFP alone under the control of the cmGAD67 promoter. Behavioral experiments were conducted three weeks later. (B) Results of the rotarod test. The latency to fall from the rotating rod was measured. (C) Results of the beam test. Mice were placed at one end of a narrow metal beam, and the time taken to traverse the beam and enter a shelter at the opposite end was recorded. Dots in the graphs represent individual mouse data points. n.s., not significant; ANOVA with Tukey's post hoc test.

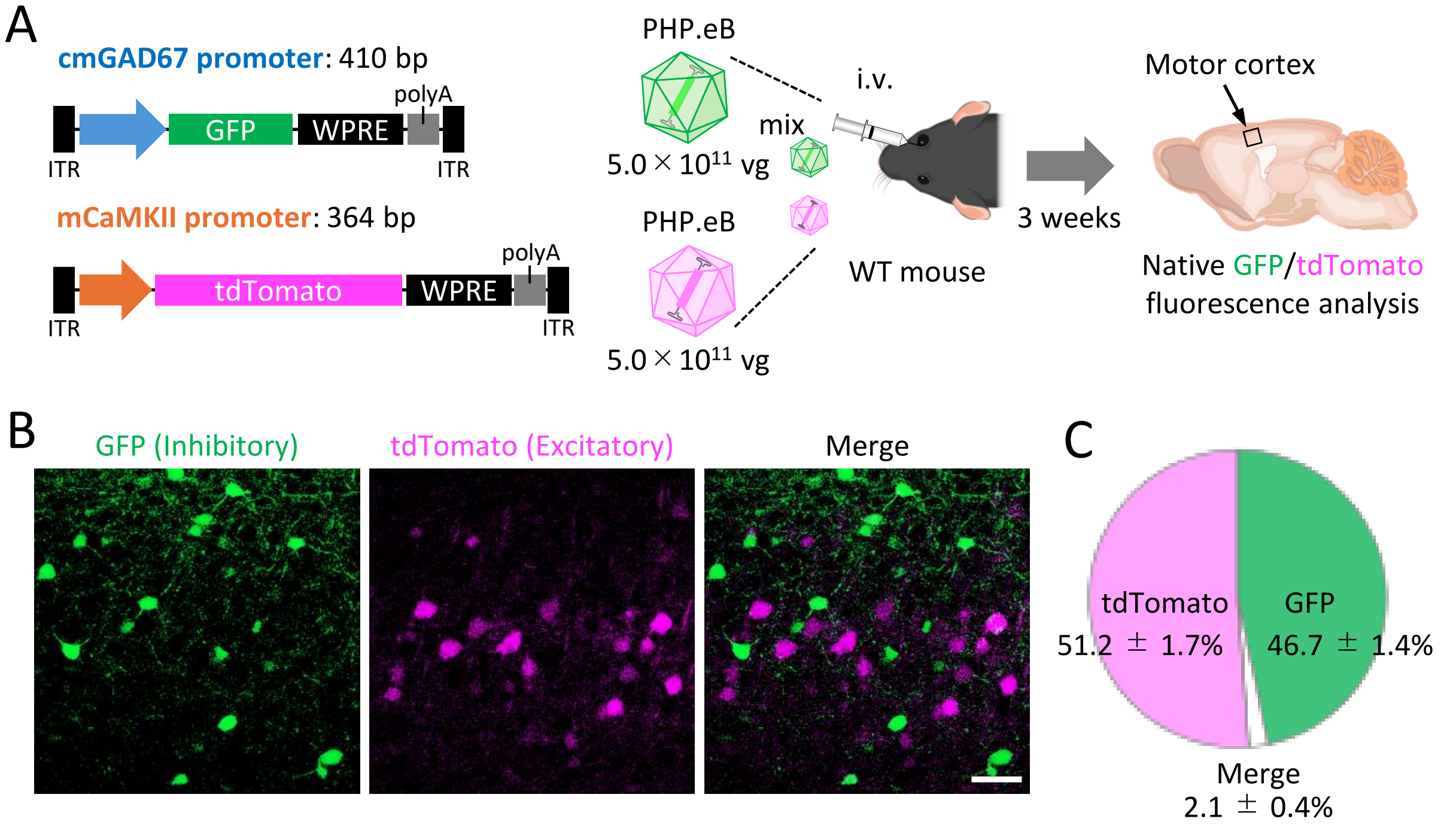

Figure S10. Complementary targeting of inhibitory and excitatory neurons using cmGAD67 and mCaMKII promoters.

(A) Schematic representation of the experimental procedure. AAV-PHP.eB expressing GFP under the control of the cmGAD67 promoter and AAV-PHP.eB expressing tdTomato under the control of the mCaMKII promoter were mixed in equal volumes (50 μL each, 1.0×10^{13} vg/mL) and administered via the orbital venous sinus. Three weeks post-injection, sagittal brain sections were prepared, and fluorescence microscopy was used to observe the primary motor cortex. (B) Fluorescence images of the motor cortex. Scale bar, 50 μm. (C) Pie chart showing the proportion of cells labeled with GFP alone, tdTomato alone, or both. Labeled cells were counted in the M1 region (542.5 μm × 722.4 μm) (n = 5 mice, 1 section/mouse). A total of 2,349 fluorescently labeled cells were counted, among which 1,094 were GFP-positive only, 1,207 were tdTomato-positive only, and 48 were co-labeled with both GFP and tdTomato.

Statistics for Main Figures

Figure 1	F, H: one-way ANOVA Dunnett's post hoc test
Figure 3	B: one-way ANOVA Turkey's post hoc test
Figure 4	D: Mann-Whitney tests<br>G: Paired t-test
Figure 5	B: Pearson correlation<br>C (b, c), D (b,c), E (b,c): Paired t-test<br>F, G: one-way ANOVA Turkey's post hoc test<br>H: Multisample E-statistic (Energy) Test of Equal Distributions
Figure 6	B, C: one-way ANOVA Turkey's post hoc test
Figure 7	B, E: two-way ANOVA repeated measures (Group effect)<br>C, F: Gehan-Breslow-Wilcoxon test
Figure 8	B, F, G: one-way ANOVA Turkey's post hoc test<br>H: Multisample E-statistic (Energy) Test of Equal Distributions

Statistics for Supplemental Figures

Figure S3	D, E: one-way ANOVA Dunnett's post hoc test
Figure S5	E, F, G: one-way ANOVA Dunnett's post hoc test
Figure S6	B, C: Unpaired t-test
Figure S7	C, D: one-way ANOVA Turkey's post hoc test
Figure S8	C: one-way ANOVA Turkey's post hoc test
Figure S9	B, C: one-way ANOVA Turkey's post hoc test

Supplemental Video legends

Video S1. Behavior in GFP control mice after DCZ administration.
Representative behavior of a control mouse bilaterally injected into the hippocampus with AAV-PHP.eB-cmGAD67-GFP following systemic administration of DCZ. No obvious seizure-like behavior was observed.

Video S2. Seizure-like behavior induced by chemogenetic inhibition of hippocampal inhibitory neurons.
Representative behavior of a mouse bilaterally injected into the hippocampus with AAV-PHP.eB-cmGAD67-hM4D(Gi) following systemic administration of DCZ. Marked behavioral seizures were observed.

Video S3. Behavior in the DREADD-only group after DCZ administration.
Representative behavior of a mouse receiving excitatory neuron-specific Gq-DREADD in the motor cortex after administration of DCZ. Moderate seizures were induced in the absence of additional AAV treatment.

Video S4. Behavior in the GFP control group after DCZ administration.
Representative behavior of a mouse co-injected with excitatory neuron-specific Gq-DREADD and control AAV-GFP in the motor cortex after administration of DCZ. Moderate seizures comparable to those in the DREADD-only group were observed.

Video S5. Behavior in the GAD65 treatment group after DCZ administration.
Representative behavior of a mouse co-injected with excitatory neuron-specific Gq-DREADD and AAV-cmGAD67-GAD65 in the motor cortex after administration of DCZ. Seizure behavior was markedly reduced compared with the DREADD-only and GFP control groups.

Supplemental methods

Promoter construct generation
Stepwise truncations and deletions of the *Gad1* locus were performed to identify regulatory regions including Ri, Rii, and putative core promoter. Constructs were cloned into AAV-PHP.eB backbones driving GFP expression for in vivo analysis (Supplemental figure S1, S3).

RT-qPCR quantification
The mRNA measurements in supplemental figure S7C and S7D related to figure 3 were done by quantitative RT-PCR. Cortical or hippocampal samples were homogenized in TRIzol Reagent (Thermo Fisher Scientific, MA, USA) and frozen in liquid nitrogen. The total RNA was extracted by RNeasy Lipid Tissue Mini Kit (Qiagen, Hilden, Germany) and cDNA was prepared by ReverTra Ace reverse transcriptase (Toyobo, Osaka, Japan). The amount of mGAD65 mRNA was determined using THUNDERBIRD SYBR qPCR Mix (Toyobo) with real-time PCR TP970 (TaKaRa Bio, Shiga, Japan). Relative expression levels were normalized to β-actin.
The primers are as follows.
mGAD65: 5'-ACT ACT GGG TTT GAG GCA CA-3' (Forward) and 5'-TGC GCA AAC TAG GAG GTA CA-3' (Reverse)
β-actin: 5'-ACC AAC TGG GAC GAT ATG GAG AAG A-3' (Forward) and 5'-TAC GAC CAG AGG CAT ACA GGG ACA A-3' (Reverse)

Immunohistochemistry
The immunostaining shown in supplemental figure S6 were performed using the same procedures as those described for Figure 3.
The following primary and secondary antibodies were used:
・Mouse anti-NeuN (1:1,000; MAB377, Millipore, MA, USA)
・Alexa Fluor 647-conjugated donkey anti-mouse IgG (1:2,000; A32787, Invitrogen)

GABA quantification
Three weeks after injection of AAV vector or PBS, cortical (near M1) and hippocampal tissues were collected. GABA concentrations were quantified using a Mouse GABA enzyme-linked immuno sorbent assay (ELISA) Kit (ab287793; Abcam) according to the manufacturer's instructions. Brain tissue was homogenized in PBS, and total protein concentrations were measured using the Micro BCA™ Protein Assay Kit (Thermo Fisher Scientific). Samples were diluted to equal protein concentrations prior to ELISA. Absorbance values were measured using an Infinite M Nano microplate reader (Tecan, Männedorf, Switzerland). Values were normalized to protein concentration in each sample.

Behavioral paradigms
Locomotor Activity Monitoring:
Infrared beam breaks were recorded over a 24-hour period using the Locomotor Activity Measuring System (LS-5; MELQUEST, Tokyo, Japan) to assess circadian activity. Mouse activity was monitored continuously throughout the day, starting at 12:00 noon, using infrared light emitted from the device.

Rotarod and Beam Tests:
Motor coordination was evaluated using the rotarod latency to fall and beam traversal time based on previous report [2]. Three weeks after AAV or PBS administration, rotarod performance was assessed using a treadmill device (MK-610A/RKZ; Muromachi Kikai).) Each mouse was tested three times at 30-minute intervals. The rotational speed was gradually increased from 4 rpm to 50 rpm over 300 seconds, and the time to fall from the rod was recorded. For the beam test, mice were placed at one end of a narrow bar (800 mm long, 11 mm in diameter and allowed to walk toward a goal box located at the opposite end. This task was repeated three times, and the average time required to traverse the beam was recorded as a measure of motor coordination.

Supplemental References

1. Hoshino, C., Konno, A., Hosoi, N., Kaneko, R., Mukai, R., Nakai, J., and Hirai, H. (2021). GABAergic neuron-specific whole-brain transduction by AAV-PHP.B incorporated with a new GAD65 promoter. Mol Brain *14*, 33. 10.1186/s13041-021-00746-1.
2. Okada, Y., Hosoi, N., Matsuzaki, Y., Fukai, Y., Hiraga, A., Nakai, J., Nitta, K., Shinohara, Y., Konno, A., and Hirai, H. (2022). Development of microglia-targeting adeno-associated viral vectors as tools to study microglial behavior in vivo. Commun Biol *5*, 1224. 10.1038/s42003-022-04200-3.
